# Inequality, role reversal and cooperation in multiple group membership settings

**DOI:** 10.1007/s10683-021-09705-y

**Published:** 2021-03-10

**Authors:** Andreas Lange, Jan Schmitz, Claudia Schwirplies

**Affiliations:** 1 University of Hamburg, Von-Melle-Park 5, 20146 Hamburg, Germany; 2 Radboud University, Heyendaalseweg 141, 6525AJ Nijmegen, The Netherlands

**Keywords:** Voluntary contributions, Multiple public goods, Cooperation, Inequality, Role reversal, Perspective change, Lab experiment, C91, C92, D62, D63, D64, H91

## Abstract

We investigate the role of endowment inequality in a local and global public goods setting with multiple group membership and examine the effect of temporal role reversal on cooperation decisions. Subjects can contribute to a global public good which benefits all subjects and two local public goods which benefit only subjects of either their own group or the group of the other endowment type. Endowment inequality per-se decreases contributions of subjects with a high endowment to the global public good, but increases cooperation of subjects with a low endowment on their local public good, thereby aggravating income disparities. Exogenously induced role reversal for several periods affects cooperation behavior of subjects with a high endowment positively and induces them to contribute more to the global good. Cooperation in unequal environments thus appears to be more stable when all parties have experienced the public goods game from the disadvantageous perspective.

**Supplementary Information:**

The online version contains supplementary material available at 10.1007/s10683-021-09705-y.

## Introduction

Not least since Piketty (2014), problems arising from inequalities within and between societies are at the heart of many public debates. A main challenge is the impact of inequalities on the provision of public goods. For many global and societal challenges, economic actors differ both in the degree to which they are affected by such issues as well as in the capacity to address them. Compared with the poor, rich individuals within a society, for example, are better able to resolve inequalities and tackle global and societal problems by redistributing wealth or financing public goods (see, e.g., Alesina and Giuliano 2011; Cohn et al. 2019). Yet, rich individuals contribute proportionately less than poor in experiments with a single public good (see, e.g., Buckley and Croson 2006; Chan et al. 1999; Corazzini et al. 2010; Kroll et al. 2005; Kesternich et al. 2014; Reuben and Riedl 2013) and are generally more likely to oppose redistribution (Cohn et al. 2019). Inequality may thus negatively impact cooperation and overall public good provision (Anderson et al. 2008; Cardenas 2003, 2007).^1^

In addition, Blackwell and McKee (2003), Falk et al. (2013) and Fellner and Lünser (2014) show that—without inequalities—individuals tend to give to the public goods that yield a direct benefit to their own (local) sub-group even though cooperation on the broader (global) level may be more efficient. Indeed, cooperation on the global level oftentimes yields higher social returns compared with local cooperation (to combat climate change and reduce CO2 emissions or to tackle inequalities within societies, e.g., global cooperation efforts are needed to obtain large scale social benefits whereas local cooperation such as supporting schools in the own neighborhood only have limited social impact).^2^ Thus, while intentions may be good, individuals may not always contribute to the most efficient public good but contribute to public goods closer to themselves (Smith 1976), where benefits from cooperation are directly observable (Fellner and Lünser 2014) and beneficiaries from cooperation share common characteristics (see, e.g., Balliet et al. 2014; Fu et al. 2012; Goette et al. 2006, 2012; Lane 2016).

The tendency of individuals to cooperate with their peers and on a local level may exacerbate the negative effects of inequality on global cooperation and public goods provision. Since it is impossible and undesired to eliminate inequality per-se, it is important to investigate new and creative measures that foster cooperation between rich and poor and promote efficient public good provision. Raising the awareness about the situation of others and give people the opportunity to (at least partially) experience the situation of the opposite party is an often promoted instrument aiming at improving cooperation between different actors. Study admissions and employers around the world, for example, value volunteering of applicants in poor communities.^3^ Moreover, several governmental programs exist that build on using experience to foster cooperation between individuals from different cultures.^4^ Further, some governments offer individuals to volunteer and perform social work as an alternative to mandatory military service (see, e.g., alternative civilian service in countries like Austria, Finland, Greece Israel, Switzerland, South Korea). In addition, experimental research on bargaining games (Brosig et al. 2003; Charness and Rabin 2002), trust games (Burks et al. 2003) and dictator games (Diekmann 2004) documents that experiencing the other position, i.e., role reversals may affect outcomes and increase trust and pro-social behavior.

The idea that experiencing “the other side” and “walking in someone else’s shoes” may change the perspective and lead to different actions, seems to be a deeply rooted belief within societies.^5^ Numerous fairy tales and fictional stories build on the potential power of temporal role reversal on cooperation and generosity. “King Thrushbeard”, “The Mouse, the Bird and the Sausage” (both by the Brother’s Grimm), “The Life of Henry the Fifth” (by William Shakespeare), “The Prince and the Pauper” (by Mark Twain), as well as the movie “Trading Places”^6^ and the TV show “Rich House, Poor House”^7^ are just some examples of many that show the deep rooted belief that experience and role reversal shapes understanding and cooperation.^8^ However, such new experiences and role reversals are not confined to fiction or individual choice.

Because of exogenous events like job loss, health shocks or economic downturns, individuals may be forced to temporarily experience relative changes in their (income) position. Assuming that the great emphasis and value government programs and admissions put on this experience and that findings from other experimental games on role reversal transfer to public good games, it seems plausible that role reversal may affect cooperation between rich and poor positively.

In this paper, we provide (to our knowledge) first experimental evidence on the effect of income heterogeneity between groups on contributions to multiple (local and global) public goods. In addition, we provide empirical evidence on the causal effect of role reversal on cooperation and public good contributions.

To study how income inequality affects cooperation and public good provision in a multiple public goods setting and to identify the causal effect of role reversal on contributions we implement a linear public goods game consisting of three parts. Participants can allocate their endowment to a private account that only benefits themselves, a global public good that benefits all subjects (high and low endowment types), a local public good that benefits only subjects of their own endowment type (high or low endowment) and another local public good that benefits subjects of the other endowment type (low or high endowment). In the *Equal Endowment* treatment (EE), subjects receive the same endowment in all three parts of the experiment. Two *Inequality* conditions (IE) explore role reversal: in our *Constant Role* treatment (CR) subjects receive a high endowment or a low endowment in the first part of the experiment and remain in their endowment positions throughout the three parts of the experiment. In our *Temporary Role Reversal* treatment (TRR), subjects receive a high or a low endowment in the first part of the experiment. In the second part of the experiment, roles are temporally reversed, i.e., endowment positions change. The role reversal is revoked and subjects change back to their initial endowments in the third part of the experiment. Thus, subjects who have a high (low) endowment in the first part of the experiment, experience a low (high) endowment in the second part of the experiment and have again a high (low) endowment in the third part of the experiment.

Comparing decisions under equal endowment (EE) and inequality (IE) in the first part of our experiment allows to identify the effects of inequality on voluntary contributions when cooperation is possible at different levels. We find that, compared to subjects who have low endowments, subjects with a high endowment contribute lower shares of their endowment to both the local and the global public good. Moreover, subjects with a high endowment in the IE treatment contribute substantially less to the more efficient global public good compared with subjects in the EE treatment. In addition, inequality leads to higher contributions to the own local public good by subjects with a low endowment. These decisions result in a decline of average income in the presence of inequality.

Comparing behavior in the treatments with inequality (CR vs. TRR) allows to assess the impact of role reversal on contribution rates and cooperation. Consistent with the evidence presented in the extant public goods literature, contributions to public goods decrease over time (see Chaudhuri 2011; Ledyard 1995, for reviews on the literature). The negative time trend, however, is less pronounced in the treatment in which subjects temporarily change endowment positions. Having experienced a role reversal in part two, has a positive effect on the contributions of subjects with a high endowment to the global public good in part three. Consequently, role reversal also decreases the income gap between high and low endowment players.

Our paper contributes to the literature in three ways: first, we complement the existing literature on public goods games and inequality which thus far studied cooperation and contributions under inequality in single public good games (see, e.g., Buckley and Croson 2006; Chan et al. 1999; Corazzini et al. 2010; Kroll et al. 2005; Kesternich et al. 2014; Reuben and Riedl 2013). Second, we add to the literature on cooperation and multiple group membership (Blackwell and McKee 2003; Falk et al. 2013; Fellner and Lünser 2014). We contribute to these literatures by studying endowment inequality in multiple group membership settings. We show that rich subjects contribute less than subjects with a low endowment and subjects who experience the situation without inequality in the EE treatments. Our setting allows us to quantify the effect of inequality on contributions to public goods. Heterogeneity in endowment between groups exacerbates income disparities and leads to lower average incomes compared with a situation of endowment equality. Third, we add to the experimental literature by providing first evidence on the effects of (temporal) role reversal in a public good setting. Studies on such role reversals in bargaining experiments (Brosig et al. 2003; Charness and Rabin 2002) and trust games (Burks et al. 2003) show that behavior may be influenced when roles are exchanged. Diekmann (2004) investigate pro-social behavior in dictators games when dictators also experience the role of a receiver. Cassar and Klein (2019) shows that redistribution behavior of subjects is affected by experiencing good and bad luck. We show that role reversal increases cooperation between heterogeneous groups of subjects. Mechanisms that increase trust or pro-social behavior may therefore also be used to foster cooperation in public goods games. Consequently, interventions studied in other experimental games may thus be transferable to public good settings.

Our findings also have implications for policy makers and practitioners. Many cooperation problems exist in which some parties may temporarily be better able to cooperate than others. Within a given supply chain, for example, volatile business cycles may make some firms better able to cooperate and show leniency to business partners than others. Our positive effect of (temporary) role reversal suggests that cooperation may be more stable in volatile environments in which the relative ability of actors to cooperate changes. In case of economic shocks, this may inform whether or not (governmental) interventions may be needed (or should prioritize on other less volatile industries). Additionally, our findings may inform policymakers interested in reducing inequality on the societal level. Although such strong forms of role reversal may be impossible to impose in practice, fostering the interaction between rich and poor within a society might be an adequate tool to increase support for the provision of public goods benefiting society as a whole.^9^

The remainder of this paper is organized as follows. Section 2 presents our experimental design. Results are presented in Sect. 3. Section 4 provides a concluding discussion.

## Experimental design and procedure

We set up a local and global public goods game (see, e.g., Blackwell and McKee 2003; Fellner and Lünser 2014, for experiments using a similar set-up) to study how income inequality between actors and temporal role reversal, i.e. a temporal change in endowment positions affect cooperation on different group levels.

This design choice was inspired by frequent real life situations where individuals belong to several groups at the same time. Literature from in-group favoritism indeed shows that individuals have a tendency to cooperate with peers who share similar characteristics (see, e.g., Balliet et al. 2014; Fu et al. 2012; Goette et al. 2006, 2012; Lane 2016). Balliet et al. (2014) and Lane (2016) perform meta analysis on in-group favoritism and show that imposed differences between groups may increase in-group favoritism.^10^ Relying on a local and global public good game with endowment differences between the local groups, our design choice not only enables us to study cooperation in multiple group membership settings but also helps from a methodological point of view as the differentiation between low and high endowment groups becomes salient for subjects. The temporary role reversal thus specifically forces subjects to experience cooperation on the local and the global level when their endowment is low and vice versa.

Our experiment thus has three key features and allows us to estimate the causal effects of experiencing another endowment perspective on cooperation: (i) By comparing homogenous and heterogeneous endowment settings, we elicit the effect of income inequality on cooperation of rich and poor subjects separately. (ii) By exogenously forcing subjects to change roles, we rule out selection and subjects experience the benefits or disadvantages of the respective other income category. (iii) By letting subjects switch back into their initial role, we study whether experiencing a new role has an effect on behavior over time, i.e., when subjects are back to their initial role.

### Experimental design

**Fig. 1:**
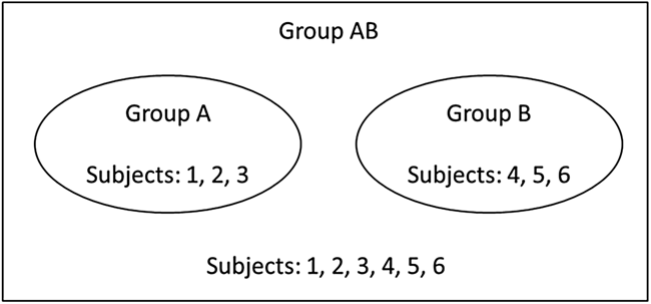
Constitution of the local and the global groups. A global group AB consists of six subjects. The numbers 1, 2, 3 represent three of the six subjects that belong to group A, and the numbers 4, 5, 6 represent three of the six subjects that belong to group B. The circles represent the two groups A and B and also highlight which subjects benefit from the respective accounts A and B, i.e., the local public goods. All subjects benefit from contributions to the account AB, i.e., the global public good

In all treatments, subjects are randomly divided into global groups of six (group AB). Within a global group of six, two local groups of three subjects are randomly formed: group A and group B. Figure 1 graphically demonstrates the group composition.

All six players of a global group face the same decision. They can either keep their endowment or contribute to three accounts: a local public good “A” that only benefits subjects in the local group A, a local public good “B” that only benefits subjects in the local group B, and a global public good “AB” that benefits all subjects in the global group of six. The contributions by individual *i* are denoted by $g_i^A$, $g_i^B$, and $g_i^{AB}$, respectively. The payoff of subject *i* that is a member of group $G\in \{A,B\}$ is given by:

$$\begin{aligned} \Pi _{i}^{G}=m^{G}-g_{i}^{A}-g_{i}^{B}-g_{i}^{AB}+\alpha \sum _{j=1}^{6}g_{j}^{G} +\beta \sum _{j=1}^{6}g_{j}^{AB}. \end{aligned}$$

Here, $m^{G}$ denotes the endowment of subjects in group *G* which may differ between group A and group B. $\alpha$ is the marginal per capita return (MPCR-Local) of contributions to the local public good of group *G*, and $\beta$ is the marginal per capita return for contributions to the global public good AB, with $\alpha =0.5$ and $\beta =0.35$. These parameters have been chosen because they have typical public good properties, $\alpha<1<3\alpha$ and $\beta<1<6\alpha$, such that contributions are beneficial to the respective group of 3 (local) or 6 (global) players, while individually freeriding is a dominant strategy. Additionally, our parameter choice was inspired by Fellner and Lünser (2014) who choose $\alpha$ and $\beta$ such that contributions to the global public good are more efficient in generating total payoff than contributions to the respective local public goods. Our parameter choice of $3\alpha <6\beta$ fulfills this criterion. Our payoff structure also implies that subjects do not benefit if they contribute to the local public good of the other group, i.e. if a subject in group A contributes to account B or a subject in group B contributes to account A. Since subjects do not materially (directly or indirectly) benefit from this contribution it resembles an (altruistic) selfless donation to the other group.

**Fig. 2:**
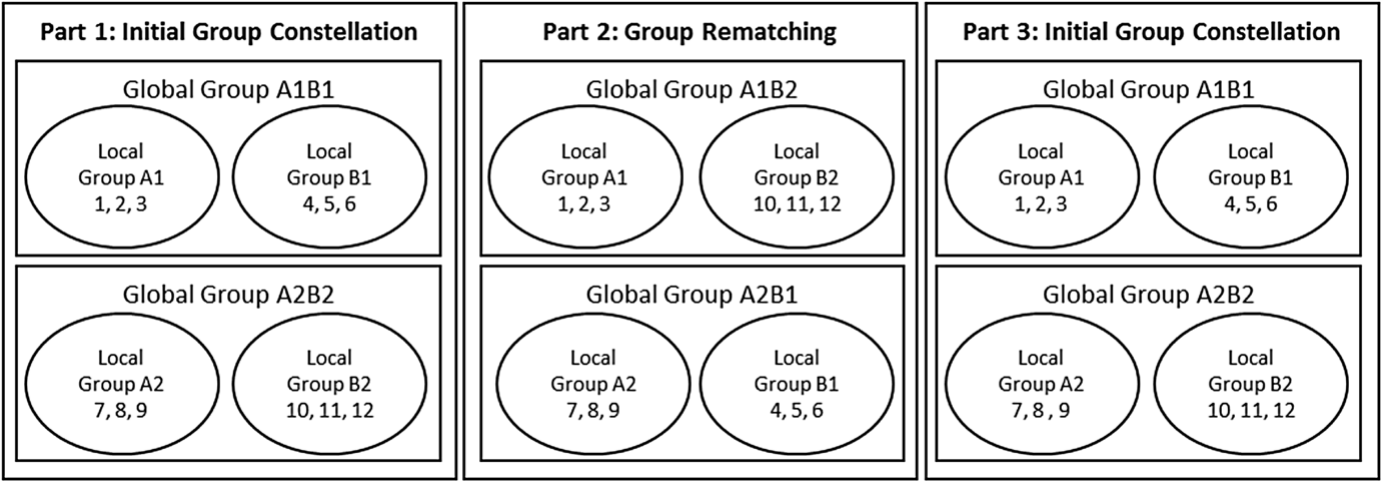
Constitution of the global and local groups in the three parts. In part 1, six subjects [denoted 1, 2, 3, 4, 5, 6 (for simplicity)] are in the same global group A1B1. Subject 1, 2, 3 form the local group A1 and subjects 4, 5, 6 constitute the local group B1. Six other subjects (denoted 7, 8, 9, 10, 11, 12) equivalently constitute the global group A2B2. In part 2, subjects in group A1 are matched with subjects from group B2. Subjects from group A2 are matched with subjects from group B1. Hence, subjects 1, 2, 3, 10, 11, and 12 form the global group A1B2 and subjects 4, 5, 6, 7, 8, and 9 form the global group B1A2. In part 3, subjects switch back in their roles and the initial group constellation from part 1 is reinstalled

In all our treatments, subjects play the local and global public goods game in three parts of five periods each. Thus, the experiment consists of 15 periods. At the beginning of the first part subjects learn whether they are in group A or group B. In part 2, we randomly rematch groups A and B in a new global group. Specifically, all subjects in group A(B) are matched with subjects from a different group B(A) than in part 1 and play the game for additional five periods. Figure 2 demonstrates this procedure graphically.^11^ After part 2, groups are rematched with the same group B(A) as in part 1 and play the game for five more periods in part 3 in the initial constellation from part 1. This matching procedure is the same in all treatments.

We implement three different treatments in order to study the effects of heterogeneous endowments and role reversal. In our *Equal Endowment Treatment (EE)* condition, subjects in group A and group B receive the same endowment ($m^{A}=m^{B}=30$) and play the local and global public goods game in three parts of five periods each. To have a clean comparison to the other treatments, we match the three subjects of each local group with three other subjects in part 2 as described in Fig. 2. In part 3, they are rematched with the same subjects as in part 1. Total wealth is equal within each global group in all treatments. Hence, we have equality of income in our EE treatment, while we establish income heterogeneity between groups in the other two treatments.

In the *Constant Role (CR)* treatment, we keep the total endowment as in *EE*, but implement endowment heterogeneity: Subjects in group A receive an endowment of $m^{A}=40$, while subjects in group B receive an endowment of $m^{B}=20$. We apply the same matching mechanism as described above and endowments remain the same throughout the three parts. Subjects in group A who receive a high endowment in part 1 also receive a high endowment in part 2 and part 3 and subjects in group B who receive a low endowment in part 1 also receive a low endowment in parts 2 and part 3. In other words, subjects stay in their role as being rich or poor throughout the experiment. The procedure, however, is similar to that in the *EE* (and *TRR*) treatment as the global groups consist of new subjects in part 2. In other words, subjects from each local group are matched with three other subjects from another local group in part 2 and rematched again with the same subjects as in part 1 in part 3.

Finally, we implement the *Temporal Role Reversal (TRR)* treatment. Subjects receive the same endowment as in the CR treatment in part 1 of the experiment. That is, subjects in group A are rich and subjects in group B are poor ($m_{{\textit{part}}1}^{A}=40$ and $m_{{\textit{part}}1}^{B}=20$). In part 2, subjects change roles and endowments. Specifically, we apply the same matching mechanism as in the other treatments but we also change the endowment assignment among subjects. Hence, we exogenously change the income and thus the roles of both rich and poor subjects.^12^ Subjects who have a high endowment of 40 points in part 1 have the low endowment of 20 points in part 2 and subjects who have a low endowment of 20 points in part 1 have a high endowment of 40 points in part 2. In part 3, subjects switch back into their initial role from part 1 ($m_{{\textit{part}}3}^{A}=40$ and $m_{{\textit{part}}3}^{B}=20$). The endowment of all subjects in all parts is common knowledge. Hence subjects in the different treatments know the endowment of others in their own local group, the other local group and the total endowment in the global group.

Part 1 of our experiment allows us to study the effect of income heterogeneity in the CR and TRR treatments compared to our baseline condition in which subjects have the same endowment (EE treatment). Table 1 summarizes the main treatment variation as well as the number of sessions conducted. The table also highlights the number of subjects who participated in each session and the number of independent observations on the part 1 global group (before two global groups interact) and the matching group level per treatment (two global groups constitute one matching group).

**Table 1:** Treatments and endowment parameters in the three parts of the experiment

Part	Treatment					
	Equal endowment (*EE*)		Constant roles (*CR*)		Temporary role reversal (*TRR*)	
	All Subjects		Group A	Group B	Group A	Group B
1	30		40	20	40	20
2	30		40	20	20	40
3	30		40	20	40	20
Sessions	5		7		7	
Subjects	120		168		168	
Global groups	20		28		28	
Matching groups	10		14		14	

The table presents the different treatments and corresponding endowments of subjects in the different parts of the experiment. Furthermore, the table presents the number of sessions conducted in each treatment, the number of subjects in a treatment and the resulting number of observations at the matching group level. In the EE treatment, subjects have an endowment of 30 in all three parts of the experiment. In the CR treatment, rich subjects have an endowment of 40 and poor subjects have an endowment of 20 in all parts. In the TRR treatment, rich subjects and poor subjects change endowment positions in part 2

### Experimental procedure

The experiment was programmed in z-Tree (Fischbacher 2007) and conducted at the University of Hamburg experimental laboratory with 456 students from various disciplines between July and November 2015. The University of Hamburg uses the software hroot for recruiting subjects (Bock et al. 2014). Each of our three treatments consists of three parts of five periods each (a total of 15 periods). In total, we conducted 19 sessions with 24 subjects in each session (see Table 1). 47% of the subjects were male, subjects were, on average, 25 years old and self reported to have an average net income of 615 Euro/month at their disposal.

During the experiment subjects earned points, which were exchanged in Euro after the experiment (20 points $=$ 1.50 Euro). One period of each part of the experiment was randomly chosen for payments. This random draw was made after all parts had been concluded. Payments of subjects averaged at 12.67 Euro. The sessions lasted about 1 h.

The experimenter was the same in all sessions and the experiment only started after all subjects answered a set of control questions. Following common practice in the literature (see, e.g., Abeler et al. 2011), instructions for each part of the experiment were handed out before the respective part started.^13^ Thus, subjects knew that the experiment consisted of three parts, but did not know the exact rules of parts 2 and 3 when the experiment started.^14^ This procedure was applied in all treatments and sessions.

After instructions for part 1 were publicly announced, subjects were randomly divided into matching groups of 12 by the computer software. Within a matching group of 12 subjects, two groups of six (global groups) were randomly formed as described above. In all treatments, subjects received information about the income from their private account, total contributions of all subjects in their group of six to the local public goods (A and B) and the global public good (AB), their income from the public goods and their total income at the end of each period. Between the three parts of the experiment, no information about average incomes and average contributions to public goods in the respective part was provided to subjects.

To ensure that all subjects understand the rules of the game and the differences in efficiency between the local and the global public good, we provided subjects with multiple contribution and payoff scenarios in the instructions. Furthermore, subjects needed to answer a set of control questions before the experiment. The experiment only started after all subjects successfully answered the control questions.

### Expected results

We discuss expected results based on existing findings from the literature. Our experiment allows to study two effects: first, we study how endowment inequality between groups affects cooperation and payoffs in a multiple group membership setting. Second, we study how a temporal change in endowment positions (temporal role reversal) affects cooperation and payoffs in this setting.

The literature on heterogenous endowments consistently reports that subjects with a low endowment contribute larger shares of their endowment to public goods (Buckley and Croson 2006; Chan et al. 1999; Corazzini et al. 2010; Kroll et al. 2005). We therefore expect within our multiple group membership setting that subjects with a low endowment are more cooperative than subjects with a high endowment when contributing to the global public good. The presence of multiple public goods may, however, limit the effects of endowment heterogeneity. As cooperation tends to be more likely to occur on the sub-group level rather than the global level when subjects have equal endowments (Falk et al. 2013; Fellner and Lünser 2014), introducing heterogeneous endowments does not imply heterogeneity regarding the public good that receives most contributions. As such, the impact of heterogeneity in our multiple public good setting remains an open question. This applies to both effect on indidividual contributions as well as on final payoffs. We empirically explore this by comparing Part 1 results between the EE treatment and the two treatments with endowment inequality, i.e. CR and TRR.

Consistent with the literature on single public goods, we expect contributions to decline in all parts and all treatments of the experiment (see, e.g., Chaudhuri 2011; Ledyard 1995, for reviews on the literature and a discussion of decreasing contributions over time). In spite of global cooperation being more efficient, the literature on local and global public goods (see, e.g., Blackwell and McKee 2003; Fellner and Lünser 2014) and in-group favoritism (see, e.g., Balliet et al. 2014; Lane 2016) suggest that in our multiple group membership setting, contribution rates on the local public goods are positive.

The temporal role reversal subjects undergo in the TRR treatment forces subjects to experience the game from the other endowment position. We expect that temporal role reversal may reduce in-group favoritism as subjects take both roles and, thus, the differentiation to the other endowment category may impact behavior less. In addition, when subjects change roles they may adjust to the behavior of the other endowment category. Thus, subjects who change from a high (low) endowment to a low (high) endowment may become more (less) cooperative compared with subjects who do not change roles. Additionally, role reversal may benefit the global public good provision if temporal role reversal indeed reduces in-group favoritism. While we expect a positive effect of TRR on cooperation and payoffs, the size of the effect also depends on whether subjects who change roles from a high endowment to a low endowment are affected more by the temporal role reversal than subjects who change from a low endowment to a high endowment. Because we expect contributions to decline between all parts of the experiment (as common in public goods games), we employ a difference-in-difference approach to evaluate the impact of the TRR on the overall expected decline in cooperation and payoffs across parts.

## Results

We concentrate on reporting the contributions relative to the endowment, i.e., the share of endowment contributed to the respective public goods. Specifically, we report *Share Own Local PG*, i.e. the share of the endowment contributed to local public good A for subjects in group A and local public good B for subjects in group B; the mean share of contributions to the public good of the other local group (*Share Other Local PG*—local public good B for subjects in group A and local public good A for subjects in group B) and the mean share of endowment contributed to the global public good (*Share Global PG*). Additionally, we state *Share Private* as the variable representing selfishness. All these shares add up to 100%. We are mainly interested in how inequality and role reversal affect cooperation behavior. These respective shares allow to derive meaningful inferences about cooperation behavior taking the different endowment levels in EE and the treatments with inequality in endowments (IE) into account. For completeness, however, we report summary statistics and regression results using absolute contributions to public goods as the dependent variable in the appendix. Our results are similar when using absolute contributions to public goods, which highlights that our treatments also influence overall public good provision. Additionally, we report payoff levels to obtain insights into the welfare and distributional effects of the respective treatment conditions.

### Local and global public goods and endowment heterogeneity

Table 2 presents summary statistics for the mean share of endowment contributed to the public goods and the average payoffs at the global group level for both the EE treatment and the inequality treatments in part 1. As our treatment intervention between the TRR and CR happens in part 2 of the experiment, all subjects in TRR and CR face the same task in part 1, such that we can pool the data to analyze the effects of income inequality on the provision of local and global public goods.^15^ Column 1 of table 2 presents part 1 global group averages in EE. Column 2 of the table shows summary statistics for all subjects (high and low endowment pooled) in the IE treatments. Columns 3 shows the mean of global group averages for subjects with a high endowment, while data for subjects with a low endowment is given in column 4.

**Fig. 3:**
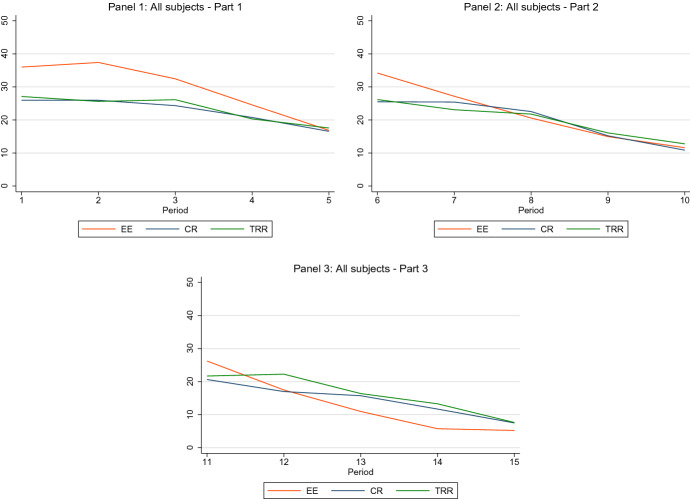
All subjects: share of endowment contributed to the global public good over time. Notes: Share of endowment contributed to the global public good by all subjects over time. *Red line*: contributions in the Equal Endowment treatment. *Blue line*: contributions in the CR treatment. *Green line*: contribution in the TRR treatment. **Panel 1**: share of endowment contributed in part 1. **Panel 2**: share of endowment contributed in part 2. **Panel 3**: share of endowment contributed in part 3

Table 3 presents results from linear regressions with cluster robust standard errors (at the global group level): we consider the share of endowment contributed to the own, other and global public good as well as the share kept in the private account and payoffs as dependent variables. Panel 1 of the table compares average differences between the EE and the IE treatment. Panel 2 of the table presents regressions comparing behavior in the EE treatment with contribution rates of subjects with a high endowment and subjects with a low endowment in the IE treatment. All regression include period dummies to control for the negative time trend within part 1 (compare panel 1 of Fig. 3 in Appendix A)^16^

We find that subjects in EE and in IE (all subjects pooled) contribute equal shares of their endowment to their own local public good (15% in EE and 16.3% in IE; $\hbox {N}=76$, $\hbox {p}=0.35$, two-sided ranksum test at the global group level) and the local public good of the other group (1.8% in EE and 1.4% in IE; $\hbox {N}=76$, $\hbox {p}=0.66$).^17^ Contribution rates weakly differ with regard to the share of endowment contributed to the global public good that benefits all subjects (29.5% in EE vs. 23% in IE; $\hbox {N}=76$, $\hbox {p}=0.099$).^18^ Note, however, that the weak effect on global public good provision turns insignificant in the regressions reported in panel 1 of Table 3.

**Table 2:** Summary statistics by treatment in part 1

	Equality (EE)	Inequality (IE)		
	All subjects	All subjects	High endowment	Low endowment
*Share Own Local PG*	15.09	16.30	12.21	20.39
	(12.12)	(9.08)	(11.19)	(13.55)
*Share Other Local PG*	1.81	1.40	1.20	1.60
	(2.34)	(2.13)	(2.96)	(3.28)
*Share Global PG*	29.45	23.03	20.57	25.49
	(17.24)	(15.76)	(19.85)	(17.89)
*Share Private*	53.64	59.27	66.03	52.52
	(15.91)	(16.35)	(20.42)	(21.98)
*Payoff*	42.25	39.77	48.21	31.33
	(5.05)	(5.24)	(4.22)	(8.71)

Mean share of contributions to the own local public good, the public good of the other local group, the global public good and mean profit in part 1. Mean of part 1 global group averages with standard deviations in parentheses. Column 1 reports global group averages in the equal endowments EE treatment. Column 2 presents summary statistics for all subjects (high and low endowment) in the treatments with endowment inequality (TRR and CR pooled). Column 3 and column 4 state the mean of variables for subjects with a high endowment and for subjects with a low endowment in the treatments with inequality in endowments (IE)

**Table 3:** Linear regressions: share of endowment contributed to the local and global public goods, private account and payoffs—EE and IE treatment in part 1

DV	Own Local PG	Other Local PG	Global PG	Share Private	Payoff
	(1)	(2)	(3)	(4)	(5)
Panel 1: IE (all subjects) and EE					
*IE*	1.205	$-$0.414	$-$6.419	5.628	$-$2.485*
	(2.926)	(0.589)	(4.333)	(4.121)	(1.312)
Constant	19.77***	2.288***	33.76***	44.18***	44.54***
	(2.727)	(0.639)	(3.745)	(3.505)	(1.094)
Observations	2280	2280	2280	2280	2280
Period controls	Yes	Yes	Yes	Yes	Yes
Cluster	76	76	76	76	76
F	6.186	3.162	12.25	22.68	21.40
$\hbox {R}^2$	0.0113	0.00542	0.0265	0.0397	0.0266
Panel 2: IE (high and low endowment subjects) and EE					
*IE High endowment*	$-$2.889	$-$0.612	$-$8.882*	12.38***	5.951***
	(3.054)	(0.650)	(4.623)	(4.433)	(1.245)
*IE Low endowment*	5.298	$-$0.216	$-$3.956	$-$1.127	$-$10.92***
	(3.220)	(0.676)	(4.479)	(4.564)	(1.608)
Constant	19.77***	2.288***	33.76***	44.18***	44.54***
	(1.501)	(0.588)	(2.062)	(2.156)	(0.700)
Observations	2280	2280	2280	2280	2280
Period controls	Yes	Yes	Yes	Yes	Yes
Cluster	76	76	76	76	76
F	8.133	2.728	10.65	21.30	65.63
$\hbox {R}^2$	0.0311	0.00592	0.0305	0.0621	0.297

Ordinary least squares (OLS) regressions with period controls and cluster robust standard errors in parentheses. Standard errors clustered at the global group level. *$p<0.1$, **$p<0.05$, ***$p<0.01$. Dependent variables: share of endowment contributed to the own local public good [column (1)]; share of endowment contributed to the local public good of the other group [column (2)]; share of endowment contributed to the global public good [column (3)]; share of endowment not contributed to any public good [column (4)]; payoffs [column (5)]. *Panel 1* Regressions comparing behavior of all subjects in the EE treatment (benchmark condition) with subjects in the IE treatment. *Panel 2* Regressions comparing behavior of all subjects in the EE treatment (benchmark condition) with subjects with a high endowment and subjects with a low endowment in the IE treatment. F-test for differences between *IE High endowment* and *IE Low endowment* in panel 2. F-test: Own Local PG: $\hbox {F}(1,75)=13.09$, $\hbox {p}<0.01$; Other Local PG: $\hbox {F}(1,75)=0.42$, $\hbox {p}=0.52$; Global PG: $\hbox {F}(1,75)=3.13$, $\hbox {p}=0.08$; Share Private: $\hbox {F}(1,75)=14.02$, $\hbox {p}<0.01$; Payoff: $\hbox {F}(1,75)=205.8$, $\hbox {p}<0.01$

We are particularly interested in the difference in cooperation by low and high endowment types. The regression results in panel 2 of Table 3 reveal that contribution rates differ depending on whether subjects have a high or a low endowment in the IE treatment.^19^ Subjects with a high endowment contribute significantly less (post estimation F-test: $\hbox {F}(1,75)=3.13$, $\hbox {p}=0.08$) to the global public good that benefits all subjects compared with subjects with a low endowment (High endowment: 20.6% vs. low endowment: 25.5%). Subjects with a high and a low endowment also differ with respect to the share of endowment contributed to their own local public good (High endowment: 12.2% vs. low endowment: 20.4%). This difference is significant (post estimation F-test: $\hbox {F}(1,75)=13.09$, $\hbox {p}<0.01$).^20^ Correspondingly, rich types keep significantly more in their private account ($\hbox {p}<0.01$).

Subjects with a high endowment thus behave relatively less cooperatively compared with subjects with a low endowment. This effect of endowment heterogeneity on contribution rates of subjects with a high and a low endowment appears to be consistent with the existing literature on heterogeneous endowments in standard public goods games (see, e.g., Buckley and Croson 2006; Chan et al. 1999). Our findings thus extend this pattern to multiple group membership settings: interestingly the differences in cooperativeness apply to both, the global as well as the local public good.

We additionally compare the endowment shares allocated to the respective public goods by low or high types with those in the EE treatment. While both types tend to contribute a smaller share to the global public good than in the EE treatment, the low endowment types partly move towards their own local public good such that the share kept in the private account is identical to subjects in the EE treatment. High types tend to contribute less to both local and global public good than in the EE treatment (even though not significant for the local public good) and thus are significantly more selfish relative to EE treatment (share private account EE vs. IE high, $\hbox {p}<0.01$).

We summarize these findings as Result 1:

#### Result 1

(Endowment heterogeneity and contributions to local and global public goods) Subjects with high endowment contribute significantly smaller shares of the endowment to both the global and the local public good than subjects with low endowment. High endowment types keep a larger share in their private account relative to the equal endowment condition. Low endowment types allocate the same share as their equal endowment counterparts, but reallocate contributions towards their local public good.

We finally discuss payoff differences arising from endowment heterogeneities. Averaged across all subjects, payoffs are significantly (at the 10% level in regression, $\hbox {p}=0.04$ in two sided ranksum test) higher in the EE treatment compared with the IE treatment. As such income heterogeneity appears to be detrimental to average payoffs due to the changed contribution decisions. By design, subjects with a high endowment in the IE treatments are richer than subjects with a low endowment in the IE treatments and also richer than subjects in the EE treatment. Table 3 shows that subjects with a high endowment in the IE earn significantly (at the 1% level) more than subjects in the EE treatment (IE high endowment: 48 points vs. EE: 42 points). Subjects with a low endowment in the IE (31 points) earn significantly less than subjects in the EE treatment (at the 1% level). Due to the cooperation decisions and the more cooperative behavior by low types, the resulting payoff gap of 17 points is only slightly smaller than the difference in initial endowments.

### Temporal role reversal and contributions to public goods

**Fig. 4:**
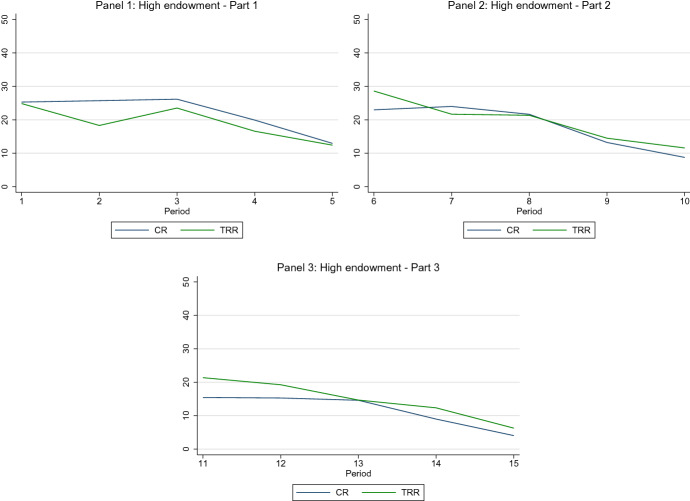
High endowment subjects: share of endowment contributed to the global public good over time. Notes: Share of endowment contributed to the global public good by subjects with a high endowment over time. *Blue line*: contributions in the CR treatment. *Green line*: contribution in the TRR treatment. **Panel 1**: share of endowment contributed in part 1. **Panel 2**: share of endowment contributed in part 2. **Panel 3**: share of endowment contributed in part 3

**Fig. 5:**
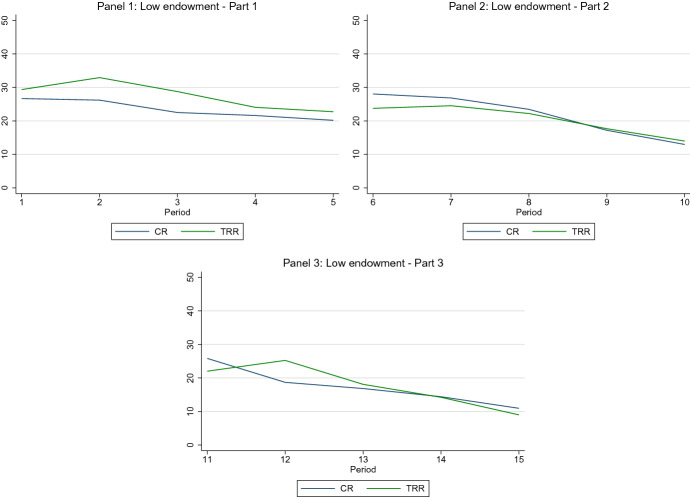
Low endowment subjects: share of endowment contributed to the global public good over time. Notes: Share of endowment contributed to the global public good by subjects with a low endowment over time. *Blue line*: contributions in the CR treatment. *Green line*: contribution in the TRR treatment. **Panel 1**: share of endowment contributed in part 1. **Panel 2**: share of endowment contributed in part 2. **Panel 3**: share of endowment contributed in part 3

**Fig. 6:**
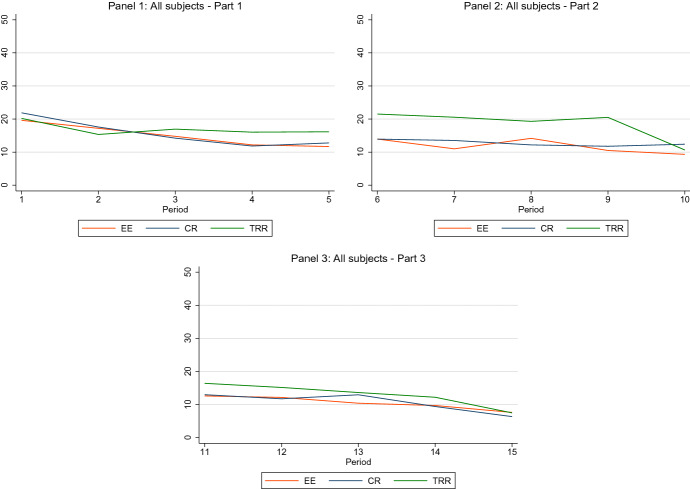
All subjects: share of endowment contributed to the own local public good over time. Notes: Share of endowment contributed to the own local public good by all subjects over time. *Red line*: contributions in the Equal Endowment treatment. *Blue line*: Contributions in the CR treatment. *Green line*: contribution in the TRR treatment. **Panel 1**: Share of endowment contributed in part 1. **Panel 2**: Share of endowment contributed in part 2. **Panel 3**: Share of endowment contributed in part 3

**Fig. 7:**
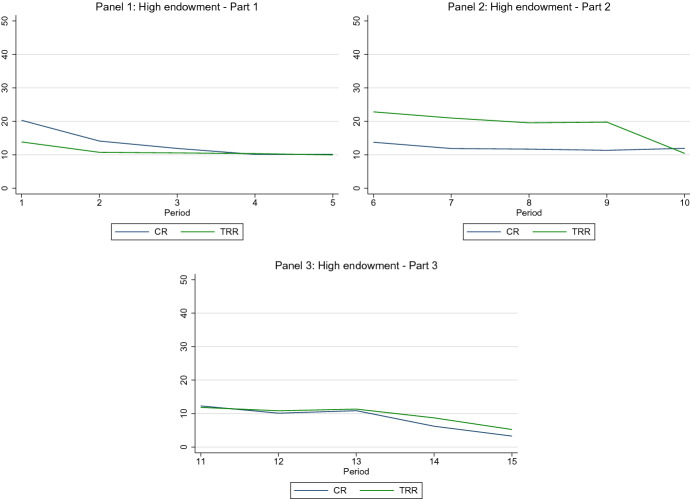
High endowment subjects: share of endowment contributed to the own local public good over time. Notes: Share of endowment contributed to the own local public good by subjects with a high endowment over time. *Blue line*: contributions in the CR treatment. *Green line*: contribution in the TRR treatment. **Panel 1**: share of endowment contributed in part 1. **Panel 2**: share of endowment contributed in part 2. **Panel 3**: Share of endowment contributed in part 3

**Fig. 8:**
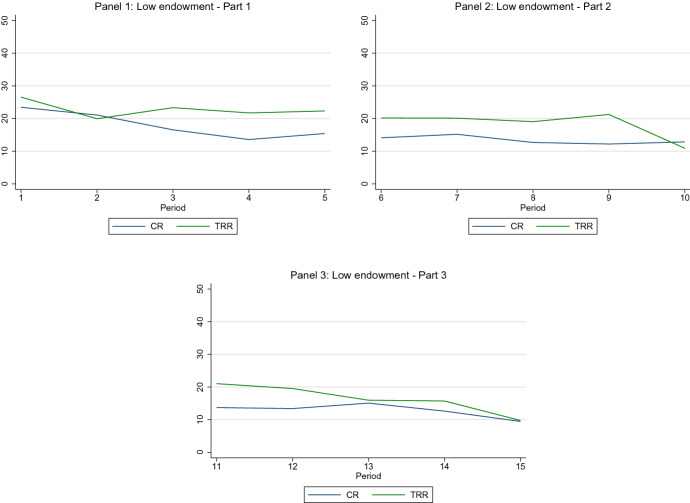
Low endowment subjects: Share of endowment contributed to the own local public good over time. Notes: Share of endowment contributed to the own local public good by subjects with a low endowment over time. *Blue line*: contributions in the CR treatment. *Green line*: contribution in the TRR treatment. **Panel 1**: share of endowment contributed in part 1. **Panel 2**: share of endowment contributed in part 2. **Panel 3**: Share of endowment contributed in part 3

We present the summary statistics of behavior in CR and TRR treaments in part 2 and 3 of the experiment in Table 4. For completeness, the table also presents the summary statistics for part 2 and part 3 in the EE treatment. In all panels, columns 1–3 present results aggregated for all subjects while columns 4–7 differentiate by endowment type. Figures 3, 4, 5, 6, 7 and 8 in Appendix A graphically illustrate the behavior over time. The figures display the share of endowment contributed to the global public good and the own local public good over parts and periods in the experiment. In line with the existing public goods literature, comparing contributions in part 1, 2 and 3 shows a negative time trend of contributions to the different public goods (see Tables 4 and 8 and the figures presented in the appendix).

**Table 4:** Summary statistics for EE, CR and TRR in part 2 and 3

	EE	All subjects		High endowment		Low endowment	
		CR	TRR	CR	TRR	CR	TRR
Panel 1: Part 2: role reversal							
*Share Own Local PG*	11.78	12.76	18.50	12.12	18.71	13.40	18.30
	(11.095)	(15.316)	(14.855)	(15.508)	(15.055)	(15.378)	(14.926)
*Share Other Local PG*	0.89	0.71	1.46	0.44	2.11	0.99	0.81
	(1.471)	(1.885)	(3.277)	(1.339)	(4.216)	(2.299)	(1.797)
*Share Global PG*	21.70	19.91	19.98	18.11	19.54	21.70	20.43
	(18.063)	(20.761)	(16.096)	(21.872)	(15.982)	(19.822)	(16.490)
*Share Private*	65.61	66.62	60.06	69.33	59.65	63.90	60.46
	(18.533)	(26.598)	(16.878)	(28.194)	(18.380)	(25.118)	(15.559)
*Payoff*	39.07	38.35	39.58	47.47	47.82	29.23	31.34
	(6.032)	(11.938)	(10.029)	(5.117)	(4.558)	(9.574)	(6.578)
Panel 2: Part 3: back to initial roles							
*Share Own Local PG*	10.51	10.70	12.99	8.554	9.595	12.85	16.39
	(10.193)	(14.634)	(13.736)	(14.507)	(9.218)	(14.704)	(16.595)
*Share Other Local PG*	0.78	0.56	1.19	0.42	0.67	0.69	1.71
	(1.556)	(1.560)	(2.872)	(1.250)	(1.754)	(1.832)	(3.627)
*Share Global PG*	13.13	14.51	16.25	11.68	14.79	17.35	17.71
	(10.750)	(20.073)	(16.959)	(19.979)	(16.234)	(20.123)	(17.828)
*Share Private*	75.58	74.23	69.57	79.35	74.95	69.12	64.18
	(11.569)	(26.347)	(20.873)	(24.593)	(17.275)	(27.477)	(22.998)
*Payoff*	36.03	36.05	37.13	45.62	46.18	26.48	28.08
	(3.265)	(11.914)	(10.744)	(5.166)	(4.142)	(8.509)	(6.940)

Mean share of contributions to the own local public good, the public good of the other local group, the global public good, and mean profit in part 2 and part 3. Mean of matching group averages with standard deviations in parentheses. Columns 1-3 present summary statistics for all subjects. Column 4 and 5 highlight the mean of variables for subjects with a high endowment in the CR and TRR, column 6 and 7 the mean of variables for subjects with a low endowment in the CR and TRR. *Panel 1* highlights mean of variables in part 2 in which low and high subjects change endowment positions in the TRR treatment. *Panel 2* depicts mean of variables in part 3 after subjects in the TRR treatment change endowment positions

**Table 5:** Difference-in-difference regressions: CR and TRR treatment—all subjects

DV	Own Local PG	Other Local PG	Global PG	Share Private	Payoff
	(1)	(2)	(3)	(4)	(5)
*TRR*	1.271	1.259**	0.628	$-$3.158	0.0243
	(2.427)	(0.545)	(4.219)	(4.357)	(1.404)
*Part 2*	$-$5.554***	0.278	$-$0.423	5.698**	$-$0.957*
	(2.036)	(0.493)	(1.922)	(2.242)	(0.562)
*Part 3*	$-$6.836***	0.126	$-$5.927***	12.64***	$-$3.389***
	(2.414)	(0.612)	(2.130)	(2.938)	(0.714)
*Part 2* $\times$ *TRR*	4.470*	$-$0.512	$-$0.554	$-$3.405	1.210
	(2.298)	(0.588)	(2.464)	(2.802)	(0.889)
*Part 3* $\times$ *TRR*	1.024	$-$0.625	1.110	$-$1.509	1.057
	(2.551)	(0.546)	(2.588)	(3.148)	(0.833)
*Constant*	20.40***	0.911**	26.23***	52.46***	41.77***
	(1.687)	(0.436)	(3.316)	(3.331)	(1.106)
Observations	5040	5040	5040	5040	5040
Period controls	Yes	Yes	Yes	Yes	Yes
Cluster	56	56	56	56	56
F	10.15	2.191	11.93	27.50	21.76
$\hbox {R}^2$	0.0221	0.00956	0.0339	0.0594	0.0283

Difference-in-difference regressions with cluster robust standard errors in parentheses. Standard errors clustered at the global group level. *$p<0.1$, **$p<0.05$, ***$p<0.01$. *Part 2* and *Part 3* are dummies capturing aggregate time trends in each part of the experiment. *TRR* is a dummy indicating that a subjects is in the TRR treatment. *TRR* captures differences between the TRR and CR treatment in part 1. *Part 2* $\times$ *TRR* and *Part 3* $\times$ *TRR* are interaction terms capturing the differential treatment effects for subjects in TRR compared to CR

In order to investigate the treatment effect of temporal role reversal on behavior, we analyze whether the decline in contributions to public goods is different in the TRR compared with the CR treatment. Table 5 presents difference-in-difference regressions with cluster robust standard errors at the global group level for all subjects (high and low endowment pooled) between the TRR and CR treatment.^21^*TRR* is a variable capturing the difference between CR and TRR in part 1. *Part 2* and *Part 3* are variables capturing the effect of part 2 and part 3 relative to part 1 in the CR treatment. The interaction terms *Part 2* $\times$ *TRR* and *Part 3* $\times$ *TRR* identify the differential treatment effect of TRR in part 2 and part 3. Our key variable of interest is the interaction effect *Part 3* $\times$ *TRR*. Table 6 presents the same regressions as Table 5 but separates subjects with a high endowment in part 1 and part 3 (panel 1) and subjects with a low endowment in part 1 and part 3 (panel 2).^22^

**Table 6:** Difference-in-difference regressions: CR and TRR treatment—high and low endowment subjects

DV	Own Local PG	Other Local PG	Global PG	Share Private	Payoff
	(1)	(2)	(3)	(4)	(5)
Panel 1: High endowment					
*TRR*	$-$2.220	1.208	$-$2.875	3.887	0.275
	(2.985)	(0.778)	(5.310)	(5.451)	(1.132)
*Part 2*	$-$3.185	$-$0.396	$-$1.435	5.015*	$-$0.0978
	(2.599)	(0.542)	(1.850)	(2.966)	(0.983)
*Part 3*	$-$6.631**	$-$0.396	$-$9.658***	16.68***	$-$1.628
	(3.178)	(0.594)	(2.785)	(3.411)	(0.996)
*Part 2* $\times$ *TRR*	8.810**	0.464	4.298	$-$13.57**	0.0807
	(4.005)	(1.148)	(4.554)	(5.456)	(1.120)
*Part 3* $\times$ *TRR*	3.262	$-$0.964	5.982*	$-$8.280**	0.282
	(2.979)	(0.789)	(3.278)	(3.446)	(1.030)
*Constant*	18.18***	0.958*	26.51***	54.35***	49.49***
	(2.439)	(0.543)	(4.454)	(4.532)	(1.086)
Observations	2520	2520	2520	2520	2520
Period controls	Yes	Yes	Yes	Yes	Yes
Cluster	56	56	56	56	56
F	6.629	2.017	6.399	17.55	13.51
$\hbox {R}^2$	0.0348	0.0166	0.0429	0.0772	0.0256
*Panel 2: Low endowment*					
*TRR*	4.762	1.310	4.131	$-$10.20*	$-$0.227
	(3.577)	(0.861)	(4.766)	(5.730)	(2.337)
*Part 2*	$-$7.923***	0.952	0.589	6.381*	$-$1.817**
	(2.896)	(0.841)	(3.305)	(3.221)	(0.841)
*Part 3*	$-$7.042**	0.649	$-$2.196	8.589**	$-$5.150***
	(3.097)	(1.039)	(3.266)	(3.956)	(1.231)
*Part 2* $\times$ *TRR*	0.131	$-$1.488	$-$5.405	6.762	2.340
	(4.556)	(0.927)	(4.227)	(6.025)	(2.057)
*Part 3* $\times$ *TRR*	$-$1.214	$-$0.286	$-$3.762	5.262	1.832
	(3.830)	(0.911)	(3.244)	(4.575)	(1.366)
*Constant*	22.62***	0.863	25.94***	50.58***	34.06***
	(2.384)	(0.711)	(4.435)	(4.369)	(1.947)
Observations	2520	2520	2520	2520	2520
Period controls	Yes	Yes	Yes	Yes	Yes
Cluster	56	56	56	56	56
F	9.570	2.012	5.844	12.28	10.09
$\hbox {R}^2$	0.0246	0.0129	0.0313	0.0616	0.0751

Difference-in-difference regressions with cluster robust standard errors in parentheses. Standard errors clustered at the global group level. *$p<0.1$, **$p<0.05$, ***$p<0.01$. *Part 2* and *Part 3* are dummies capturing aggregate time trends in each part of the experiment. *TRR* is a dummy indicating that a subjects is in the TRR treatment. *TRR* captures differences between the TRR and CR treatment in part 1. *Part 2* $\times$ *TRR* and *Part 3* $\times$ *TRR* are interaction terms capturing the differential treatment effects for subjects in TRR compared to CR. *Panel 1* Regression limited to subjects who have a high endowment in part 1. *Panel 2* regressions limited to subjects with a low endowment in part 2

Across all subjects, Table 5 shows no significant effect of temporal role reversal on contributions and payoffs. This absence of effects on aggregate behavior may not be surprising since role reversal may affect subjects who change from a high to a low endowment vs. subjects who change from a low to a high endowment differently. Separating by endowment types, Table 6 documents three interesting findings: Firstly, the variable capturing the differential effect on the share of endowment kept reveals that subjects with a high endowment in TRR are significantly (at the 5% level) more cooperative than in CR in part 3. The share kept for themselves increases (from 68% in part 1 to 74% in part 3) but the increase is substantially lower (by 8 percentage points) compared with the increase in selfishness in the CR (from 64% in part 1 to 79% in part 3). Secondly, this lower reduction in cooperation in TRR translates into higher contributions of subjects with a high endowment to the global public good (significant at the 10% level). Contributions decrease from 19% in TRR (22% in CR) of the endowment to about 15% in TRR (12% in CR). Thirdly, subjects with a low endowment do not seem to be impacted by temporal role reversal to the same extent as subjects with a high endowment. Although they reduce contributions to the global public good and keep more for themselves, the effect is moderate and statistically insignificant.^23^ We summarize these findings in Result 2:

#### Result 2

(Temporal role reversal and contributions to public goods) Temporal role reversal stabilizes cooperative behavior by subjects with a high endowment: after having temporarily experienced the low endowment in part 2, they reduce contributions to a lesser extent than subjects who do not change roles and have a high endowment throughout the experiment. This particularly benefits the global public good.

Panel 1 of Table 4 sheds some light on the mechanism through which role reversal in part 2 impacts behavior in part 3. When roles change, subjects adapt the behavior the endowment type showcases without role reversal. Poor subjects are more cooperative than rich. Thus, when rich become poor, they behave similar to others with a low endowment who do not change roles. Poor subjects who become rich also adopt to their new role and behave more selfishly. When changing back to their initial roles, part of the behavior the subjects showcased in part 2 spills over and the temporary experience appears to make high (low) endowment types more (less) inclined to cooperate on the global level in part 3. The effect of role reversal, however, seems to be stronger on rich subjects who become poor and experience the low endowment position compared with poor subjects who become rich and experience the high endowment position.

### Temporal role reversal and inequality

The changes in contribution decisions triggered by role reversal as identified in the previous section may also affect the relative payoff positions, i.e. the extent of inequality. In this section, we substantiate whether the higher cooperation of the rich by means of the global public good translates into reduced inequality between treatments. We therefore compare differential effects on payoffs between the TRR treatment and the CR treatment as well as between these treatments and the EE treatment.

**Table 7:** Difference-in-difference regressions: EE and IE (CR and TRR) treatment—payoffs

DV: Payoff	EE and CR			EE and TRR		
	Endowment CR			Endowment TRR		
	$\mathrm{High}+\mathrm{Low}$	High	Low	$\mathrm{High}+\mathrm{Low}$	High	Low
	(1)	(2)	(3)	(4)	(5)	(6)
*IE*	$-$2.497	5.813***	$-$10.81***	$-$2.473*	6.088***	$-$11.03***
	(1.630)	(1.423)	(2.223)	(1.345)	(1.323)	(1.739)
*Part 2*	$-$2.675***	$-$2.520***	$-$2.293**	$-$1.986**	$-$1.765*	$-$2.076**
	(0.841)	(0.786)	(0.896)	(0.797)	(0.886)	(0.841)
*Part 3*	$-$5.537***	$-$4.976***	$-$5.563***	$-$5.216***	$-$4.926***	$-$5.159***
	(0.701)	(0.722)	(0.788)	(0.693)	(0.747)	(0.746)
*IE* $\times$ *Part 2*	1.779*	2.588**	0.970	2.989***	2.669**	3.310
	(0.990)	(1.015)	(1.244)	(1.076)	(1.254)	(2.009)
*IE* $\times$ *Part 3*	2.523**	3.786***	1.260	3.580***	4.068***	3.092***
	(0.988)	(1.008)	(1.348)	(0.871)	(1.043)	(1.046)
*Constant*	44.79***	44.87***	45.01***	44.62***	44.40***	45.24***
	(1.100)	(1.110)	(1.108)	(1.096)	(1.145)	(1.099)
Observations	4320	3060	3060	4320	3060	3060
Period Controls	Yes	Yes	Yes	Yes	Yes	Yes
Cluster	48	48	48	48	48	48
F	23.14	27.70	28.75	26.79	30.43	24.41
$\hbox {R}^2$	0.0548	0.179	0.237	0.0546	0.180	0.228

Difference-in-difference regressions with cluster robust standard errors in parentheses. Standard errors clustered at the global group level. *$p<0.1$, **$p<0.05$, ***$p<0.01$. *Part 2* and *Part 3* are dummies capturing aggregate time trends in each part of the experiment. *IE* is a dummy indicating that a subjects is in the CR/TRR treatment. *IE* captures differences between the TRR/C and EE treatment in part 1. *Part 2* $\times$ *IE* and *Part 3* $\times$ *IE* are interaction terms capturing the differential treatment effects for subjects in TRR/CR compared to EE. *Columns 1–3*: regression limited to subjects in EE and CR (IE, Part 2 $\times$ IE, and Part 3 $\times$ IE describe the CR treatment). Column 1: regressions include all subjects in EE and CR. Column 2: observations include all subjects in EE but but only include subjects with a high endowment in CR. Column 3: Observations include all subjects in EE but only include subjects with a low endowment in CR. *Columns 4–6*: regression limited to subjects in EE and TRR (IE, Part 2 $\times$ IE, and Part 3 $\times$ IE describe the TRR treatment). Column 4: regressions include all subjects in EE and TRR. Column 5: observations include all subjects in EE but but only include subjects with a high endowment in TRR. Column 6: observations include all subjects in EE but only include subjects with a low endowment in TRR

The regression results presented in Tables 5 and 6 in the previous section show positive, but statistically insignificant coefficients for payoff of subjects with a low endowment. Thus, the changes in contributions induced by role reversal do not significantly affect income positions. Notwithstanding, payoff differences persist between treatments. Subjects with a low endowment in the TRR (28 points) still earn significantly less compared with subjects in the EE (36 points), although the gap is smaller than without role reversal (26 points).^24^

Table 7 shows the corresponding difference-in-difference regressions with cluster robust standard errors (at the global group level) and period fixed effects comparing EE and CR as well as EE and TRR. The variable *IE* represents the CR treatment in columns 1–3 and the TRR treatment in columns 4–6. The interaction terms *IE* $\times$ *Part 2* and *IE* $\times$ *Part 3* capture the differential treatment effects for EE vs. CR and TRR, respectively. Column 1 (4) includes all subjects from the EE and the CR (TRR) treatment as observations, while the other columns separate by endowment type.

The regressions show that on average, subjects in CR and TRR earned a little less in part 1 compared to EE, but significantly close this gap in part 2 and 3 (see column 1 and 4). The regressions in columns 2–3 and columns 5–6 reveal that the effect primarily benefits high endowment types in CR. Here, inequalities are further enhanced over time. Conversely, both high and low types benefit over time in the TRR treatment such that the aggregate gains in payout do not come at the expense of increased inequality. While we do not see significant differences between CR and TRR, role reversal if anything tends to moderate the income gap that would increasingly exist without a change in the endowment positions.

## Discussion and conclusion

In this paper, we provide first evidence on cooperation decisions in a public good game with multiple group memberships in the presence of income heterogeneities. Additionally, we explore the impact of temporal change in endowment positions on voluntary contributions. Subjects are simultaneously members of a local group as well as a global group. They may contribute to a local public good that benefits only subjects of their own endowment category (cooperation with peers), a local public good that benefits subjects of the other endowment category (altruistic contribution), or to a global public good that benefits all subjects (global cooperation).

The impact of inequality is established by comparing the *Equal Endowment* condition (EE) with a setting where subjects had either a high or low endowment (IE). Our experimental data suggest that inequality in multiple group membership settings leads to a lower level of contributions of subjects with a high endowment. When benchmarked against the behavior of subjects with a low endowment and against the behavior of subjects with equal endowments, these subjects particularly contribute less to the global public good which benefits all subjects. This, in turn, aggravates income disparities between subjects with a high endowment and subjects with a low endowment. Additionally, this behavior reduces aggregate payoffs between treatments with endowment heterogeneity and the equal endowment group. Existing literature documents that efficient public good provision on a global level may already be problematic without inequality between groups (see, e.g., Fellner and Lünser 2014). Inequality between groups seems to further reduce cooperation on a global level limiting the provision of efficient public goods which benefits all.

We investigated the impact of role reversal on cooperation and public good provision by comparing a *Constant Roles* condition (CR) with a *Temporary Role Reversal* condition (TRR). In TRR, high and low endowment subjects switched income positions in part 2 of the experiment, but the role reversal was revoked in the part 3 such that subjects switched back to their initial endowment position from part 1. We identify spillover effects from role reversal on behavior. Experiencing the other income position and being relatively worse/better off than the other group for a period of time affects behavior. The temporal role reversal increased contributions by subjects with a high endowment to their local but also to the global public good after initial roles are reinstalled. These additional contributions benefit subjects with a low endowment and thereby directionally moderate income inequality between groups. Subjects with a low endowment who experienced the high income position in part 2, however, were adversely affected as these subjects decreased their contributions. Yet, the average effect on subjects with a low endowment is substantially weaker. Our findings suggest that cooperation within and between heterogeneous groups is more stable if the groups also experience the situation from the viewpoint of the other group.

Our findings complement the literature on cooperation in public goods games by studying inequality in a multiple public goods setting. In addition, we contribute to the literature by showing that role reversal which affects behavior in other experimental economic games, e.g., trust, dictator and bargaining games also affects cooperation in public goods games. Our findings thus add a new mechanism which helps increasing cooperation in public goods settings with inequality to the experimental tool box.

While our results on the behavior of students in the laboratory are confined to relatively low stakes (and thus inequality levels),^25^ we may cautiously interpret our finding in light of real world cooperation problems. Many economic cooperation problems exist in which some parties may temporarily be better able to contribute than others. Our findings suggest that cooperation in volatile environments in which the relative ability of actors to cooperate changes and actors can associate with the disadvantageous position of others may be more stable. Although difficult to implement in practice, our findings may inform policymakers interested in reducing inequality at the societal level. We provide evidence that experience may (to some extent) foster cooperation. Thus, increasing the interaction between rich and poor within a society and raising awareness for the situation of the other side might be a tool to increase support for the provision of public goods benefiting society as a whole. Although forced participation in social programs that raise awareness of living conditions of others, however, may be difficult to implement some variations of such policy tools may be possible. Some countries, for instance, offer alternative social work (e.g., in poor communities) as an alternative to military service.^26^ Implementing programs with a mandatory period of social service for everyone graduating from school/in school may thus be realistic (e.g., a temporarily limited mandatory internship at a not for profit organization helping the poor). However, we identified only moderate effects from an exogenously imposed role reversal. As such the gains from encouraging people to “put themselves into the shoes of others” might be limited.

To fully assess the potential of role reversal, further research in the lab and in the field is needed. It appears important to identify determinants of changing attitudes towards cooperating at the local vs. global level, i.e. caring for people alike or people who differ.

### Electronic supplementary material

Below is the link to the electronic supplementary material.

Electronic supplementary material 1 (ZIP 3076 kb)

## Appendix

See Tables 8 and 9 and Figs. 3, 4, 5, 6, 7 and 8.

**Table 8:** Summary statistics for CR and TRR in part 1

	Part 1: Before role reversal					
	All subjects		High endowment		Low endowment	
	CR	TRR	CR	TRR	CR	TRR
*Share Own Local PG*	15.66	16.93	13.32	11.10	18.01	22.77
	(12.76)	(13.40)	(12.31)	(10.04)	(12.98)	(13.93)
*Share Other Local PG*	0.768	2.027	0.595	1.804	0.940	2.250
	(2.038)	(3.827)	(1.434)	(3.884)	(2.519)	(3.828)
*Share Global PG*	22.72	23.35	22.01	19.13	23.43	27.56
	(21.74)	(15.93)	(23.76)	(15.29)	(19.92)	(15.70)
*Share Private*	60.85	57.69	64.08	67.97	57.62	47.42
	(23.60)	(20.76)	(23.82)	(16.57)	(23.35)	(19.61)
*Payoff*	39.76	39.78	48.07	48.34	31.45	31.22
	(11.51)	(10.31)	(4.693)	(3.779)	(10.22)	(7.089)

Mean share of contributions to the own local public good, the public good of the other local group, the global public good, and mean profit in part 1. Mean of group 1 averages with standard deviations in parentheses. Columns 1 and 2 present summary statistics for all subjects. Column 1 highlights matching group averages in the CR treatment. Column 2 presents summary statistics for all subjects in the TRR. Column 3 and Column 4 highlight the mean of variables for subjects with a high endowment in the CR and TRR. Column 5 and Column 6 highlight the mean of variables for subjects with a low endowment in the CR and TRR

**Table 9:** Absolute contributions: summary statistics by treatment in part 1

	Equality (EE)	Inequality (IE)		
	All subjects	All subjects	High endowment	Low endowment
*Own Local PG*	4.528	4.480	4.882	4.079
	(3.638)	(2.697)	(4.475)	(2.711)
*Other Local PG*	0.543	0.399	0.480	0.319
	(0.704)	(0.657)	(1.186)	(0.656)
*Global PG*	8.835	6.663	8.227	5.099
	(5.174)	(4.955)	(7.941)	(3.579)
*Private*	16.09	18.46	26.41	10.50
	(4.775)	(4.990)	(8.168)	(4.395)

Mean of absolute contributions to the own local public good, the public good of the other local group, the global public good and mean profit in part 1. Mean of part 1 global group averages with standard deviations in parentheses. Column 1 reports global group averages in the equal endowments EE treatment. Column 2 presents summary statistics for all subjects (high and low endowment) in the treatments with endowment inequality (TRR and CR pooled). Column 3 and column 4 state the mean of variables for subjects with a high endowment and for subjects with a low endowment in the treatments with inequality in endowments (IE)

### Absolute contributions

See Tables 10, 11 and 12.

**Table 10:** Linear regressions: absolute contributions to the local and global public goods and private account—EE and IE treatment in part 1

DV	Own Local PG	Other Local PG	Global PG	Private
	(1)	(2)	(3)	(4)
Panel 1: IE (all subjects) and EE				
*IE*	$-0.0480$	$-0.144$	$-2.172$	2.364*
	(0.876)	(0.178)	(1.315)	(1.242)
Constant	5.941***	0.703***	10.20***	13.16***
	(0.816)	(0.191)	(1.114)	(1.054)
Observations	2280	2280	2280	2280
Period controls	Yes	Yes	Yes	Yes
F	5.820	3.242	14.36	23.15
$\hbox {R}^2$	0.0117	0.00539	0.0285	0.0334
Panel 2: IE (high and low endowment subjects) and EE				
*IE High endowment*	0.354	$-0.0636$	$-0.608$	10.32***
	(0.998)	(0.221)	(1.554)	(1.513)
*IE Low endowment*	$-0.450$	$-0.224$	$-3.736$***	$-5.590$***
	(0.877)	(0.178)	(1.233)	(1.202)
Constant	5.941***	0.703***	10.20***	13.16***
	(0.816)	(0.191)	(1.115)	(1.054)
Observations	2280	2280	2280	2280
Period Controls	Yes	Yes	Yes	Yes
F	5.227	2.701	13.58	59.08
$\hbox {R}^2$	0.0140	0.00635	0.0452	0.287

Linear regressions with period controls and cluster robust standard errors in parentheses. Standard errors are clustered at the global group level. *$p<0.1$, **$p<0.05$, ***$p<0.01$. Dependent variables: Endowment contributed to the own local public good [column (1)]; Endowment contributed to the local public good of the other group [column (2)]; Endowment contributed to the global public good [column (3)]; Endowment not contributed to any public good [column (4)]. *Panel 1*: Regressions comparing behavior of all subjects in the EE treatment (benchmark condition) with subjects in the EE treatment. *Panel 2*: Regressions comparing behavior of all subjects in the EE treatment (benchmark condition) with subjects with a high endowment and subjects with a low endowment in the IE treatment. F-test for differences between *IE High endowment* and *IE Low endowment* in panel 2. F-test: own Local PG: $\hbox {F}(1,75)=1.41$, $\hbox {p}=0.24$; Other Local PG: $\hbox {F}(1,75)=0.75$, $\hbox {p}= 0.39$; Global PG: $\hbox {F}(1,75)=10.26$, $\hbox {p}<0.01$; Private: $\hbox {F}(1,75)=195.94$, $\hbox {p}<0.01$

**Table 11:** All subjects: difference-in-difference regressions: absolute contributions to local and global public good and endowment not contributed

All subjects	Own Local PG	Global PG	Private
*TRR*	0.0321	$-0.162$	$-0.243$
	(0.722)	(1.326)	(1.336)
*Part 2*	$-1.429$**	$-0.228$	1.641**
	(0.619)	(0.505)	(0.689)
*Part 3*	$-2.030$***	$-2.151$***	4.196***
	(0.756)	(0.641)	(0.876)
*Part 2* $\times$ *TRR*	1.775**	0.319	$-2.038$**
	(0.732)	(0.849)	(0.927)
*Part 3* $\times$ *TRR*	0.531	0.820	$-1.130$
	(0.742)	(0.803)	(0.906)
*Constant*	5.898***	7.896***	15.93***
	(0.538)	(1.039)	(1.052)
Observations	5040	5040	5040
Cluster	28	28	28
Period controls	Yes	Yes	Yes
F	9.036	12.12	27.12
$\hbox {R}^2$	0.0228	0.0325	0.0402

Difference-in-difference regressions with cluster robust standard errors in parentheses. Standard errors clustered at the global group level. *$p<0.1$, **$p<0.05$, ***$p<0.01$. *Part 2* and *Part 3* are dummies capturing aggregate time trends in each part of the experiment. *TRR* is a dummy indicating that a subjects is in the temporal role reversal treatment. *Part 2* $\times$ *TRR* and *Part 3* $\times$ *TRR* are interaction terms capturing the treatment effects for subjects in TRR compared to CR. All regressions include period fixed effects (Period Controls)

**Table 12:** Rich and poor subjects: difference-in-difference regressions: absolute contributions to local and global public good and endowment not contributed

Panel 1: High endowment	Own Local PG	Global PG	Private
*TRR*	$-0.888$	$-1.150$	1.555
	(1.194)	(2.124)	(2.180)
*Part 2*	$-1.274$	$-0.574$	2.006*
	(1.040)	(0.740)	(1.187)
*Part 3*	$-2.652$**	$-3.863$***	6.674***
	(1.271)	(1.114)	(1.365)
*Part 2* $\times$ *TRR*	3.524**	1.719	$-5.429$**
	(1.602)	(1.821)	(2.182)
*Part 3* $\times$ *TRR*	1.305	2.393*	$-3.312$**
	(1.192)	(1.311)	(1.378)
*Constant*	7.271***	10.60***	21.74***
	(0.976)	(1.782)	(1.813)
Observations	2520	2520	2520
Cluster	28	28	28
Period controls	Yes	Yes	Yes
F	6.629	6.399	17.55
$\hbox {R}^2$	0.0348	0.0429	0.0772

Panel 2: Low endowment	Own Local PG	Global PG	Private
*TRR*	0.952	0.826	$-2.040$*
	(0.715)	(0.953)	(1.146)
*Part 2*	$-1.585$***	0.118	1.276*
	(0.579)	(0.661)	(0.644)
*Part 3*	$-1.408$**	$-0.439$	1.718**
	(0.619)	(0.653)	(0.791)
*Part 2* $\times$ *TRR*	0.0262	$-1.081$	1.352
	(0.911)	(0.845)	(1.205)
*Part 3* $\times$ *TRR*	$-0.243$	$-0.752$	1.052
	(0.766)	(0.649)	(0.915)
*Constant*	4.524***	5.188***	10.12***
	(0.477)	(0.887)	(0.874)
Observations	2520	2520	2520
Cluster	28	28	28
Period controls	Yes	Yes	Yes
F	9.570	5.844	12.28
$\hbox {R}^2$	0.0246	0.0313	0.0616

Difference-in-difference regressions with cluster robust standard errors in parentheses. Standard errors clustered at the global group level. *$p<0.1$, **$p<0.05$, ***$p<0.01$. *Part 2* and *Part 3* are dummies capturing aggregate time trends in each part of the experiment. *TRR* is a dummy indicating that a subjects is in the temporal role reversal treatment. *Part 2* $\times$ *TRR* and *Part 3* $\times$ *TRR* are interaction terms capturing the treatment effects for subjects in TRR compared to CR. Panel 1: Regression limited to subjects who have a high endowment in part 1. Panel 2: regressions limited to subjects with a low endowment in part 2. All regressions include period fixed effects (Period Controls)

### Tobit regressions

See Tables 13, 14 and 15.

**Table 13:** Tobit regressions: contributions to the local and global public goods and private account – EE and IE treatment in part 1

DV	Own Local PG	Other Local PG	Global PG	Private
	(1)	(2)	(3)	(4)
Panel 1: IE (all subjects) and EE				
*IE*	0.383	$-$1.999	$-$3.613***	4.858***
	(0.790)	(1.516)	(0.935)	(0.971)
Constant	0.249	$-$22.63***	6.354***	8.779***
	(0.961)	(2.612)	(1.139)	(1.206)
Observations	2280	2280	2280	2280
Period controls	Yes	Yes	Yes	Yes
Log likelihood	$-$4663.4	$-$911.4	$-$5662.8	$-$7291.6
Panel 2: IE (high and low endowment subjects) and EE				
*IE High endowment*	$-$0.0109	$-$1.266	$-$1.744*	15.41***
	(0.895)	(1.696)	(1.049)	(0.944)
*IE Low endowment*	0.752	$-$2.774	$-$5.418***	$-$6.212***
	(0.883)	(1.745)	(1.047)	(0.925)
Constant	0.244	$-$22.59***	6.388***	9.430***
	(0.963)	(2.608)	(1.130)	(1.026)
Observations	2280	2280	2280	2280
Period controls	Yes	Yes	Yes	Yes
Log likelihood	$-$4663.0	$-$911.0	$-$5655.8	$-$7005.6

Tobit regressions on censored data with period controls and upper limit on maximal contribution. *$p<0.1$, **$p<0.05$, ***$p<0.01$. Dependent variables: Endowment contributed to the own local public good [column (1)]; Endowment contributed to the local public good of the other group [column (2)]; Endowment contributed to the global public good [column (3)]; Endowment not contributed to any public good [column (4)]. *Panel 1*: regressions comparing behavior of all subjects in the EE treatment (benchmark condition) with subjects in the EE treatment. *Panel 2*: regressions comparing behavior of all subjects in the EE treatment (benchmark condition) with subjects with a high endowment and subjects with a low endowment in the IE treatment. F-test for differences between *IE High endowment* and *IE Low endowment* in panel 2. F-test: Own Local PG: $\hbox {F}(1,2274)=0.89$, $\hbox {p}=0.35$; Other Local PG: $\hbox {F}(1,2274)=0.85$, $\hbox {p}=0.36$; Global PG: $\hbox {F}(1,2274)=14.24$, $\hbox {p}<0.01$; Private: $\hbox {F}(1,2274)=615.07$, $\hbox {p}<0.01$

**Table 14:** Tobit Regressions: CR and TRR treatment—all subjects

DV	Own Local PG	Other Local PG	Global PG	Private
	(1)	(2)	(3)	(4)
*TRR*	0.931	4.848***	1.166	$-$0.146
	(0.878)	(1.458)	(1.054)	(1.071)
*Part 2*	$-$4.006***	1.359	$-$0.409	2.490
	(1.498)	(2.395)	(1.800)	(1.852)
*Part 3*	$-$5.647***	$-$0.269	$-$4.824***	6.953***
	(1.523)	(2.453)	(1.832)	(1.858)
*Part 2* $\times$ *TRR*	3.745***	$-$2.200	0.496	$-$3.358**
	(1.248)	(2.043)	(1.508)	(1.512)
*Part 3* $\times$ *TRR*	2.055	$-$1.353	1.666	$-$2.484
	(1.280)	(2.092)	(1.546)	(1.516)
Constant	$-$0.398	$-$24.06***	1.375	13.83***
	(1.048)	(2.186)	(1.277)	(1.315)
Observations	5040	5040	5040	5040
Period controls	Yes	Yes	Yes	Yes
Log likelihood	$-$9657.9	$-$1764.9	$-$10771.4	$-$15841.4

Tobit regressions on censored data. Upper limit maximum endowment. *$p<0.1$, **$p<0.05$, ***$p<0.01$. *Part 2* and *Part 3* are dummies capturing aggregate time trends in each part of the experiment. *TRR* is a dummy indicating that a subjects is in the temporal role reversal treatment. *Part 2* $\times$ *TRR* and *Part 3* $\times$ *TRR* are interaction terms capturing the treatment effects for subjects in TRR compared to CR. All regressions include period fixed effects (Period Controls)

**Table 15:** Tobit regressions: CR and TRR treatment—high and low endowment subjects

DV	Own Local PG	Other Local PG	Global PG	Private
	(1)	(2)	(3)	(4)
Panel 1: High endowment				
*TRR*	$-$0.926	6.547**	$-$0.538	2.737
	(1.705)	(2.548)	(2.153)	(2.081)
*Part 2*	$-$3.485	$-$3.208	$-$1.642	3.146
	(2.833)	(4.325)	(3.653)	(3.546)
*Part 3*	$-$8.016***	$-$2.866	$-$10.32***	14.51***
	(2.928)	(4.489)	(3.757)	(3.621)
*Part 2* $\times$ *TRR*	8.690***	3.498	5.699*	$-$10.59***
	(2.389)	(3.690)	(3.073)	(2.951)
*Part 3* $\times$ *TRR*	4.842*	$-$2.523	5.739*	$-$9.052***
	(2.493)	(3.837)	(3.178)	(3.027)
Constant	$-$1.648	$-$29.42***	2.669	22.30***
	(2.018)	(3.800)	(2.595)	(2.508)
Observations	2520	2520	2520	2520
Period controls	Yes	Yes	Yes	Yes
Log likelihood	$-$4667.5	$-$881.8	$-$5147.9	$-$6393.9
Panel 2: Low endowment				
*TRR*	2.232**	3.147**	2.551**	$-$4.332***
	(0.899)	(1.495)	(1.111)	(1.190)
*Part 2*	$-$4.365***	3.951	0.261	2.940
	(1.578)	(2.435)	(1.913)	(2.050)
*Part 3*	$-$3.951**	1.320	$-$1.458	3.934*
	(1.572)	(2.438)	(1.924)	(2.058)
*Part 2* $\times$ *TRR*	0.233	$-$5.912***	$-$3.049*	2.041
	(1.300)	(2.187)	(1.595)	(1.685)
*Part 3* $\times$ *TRR*	0.232	$-$0.313	$-$1.116	1.292
	(1.308)	(2.069)	(1.617)	(1.700)
Constant	0.485	$-$17.65***	0.737	9.947***
	(1.082)	(2.270)	(1.354)	(1.450)
Observations	2520	2520	2520	2520
Period controls	Yes	Yes	Yes	Yes
Log likelihood	$-$4635.3	$-$856.3	$-$4874.4	$-$5753.6

Tobit regressions on censored data. Upper limit maximum endowment. *$p<0.1$, **$p<0.05$, ***$p<0.01$. *Part 2* and *Part 3* are dummies capturing aggregate time trends in each part of the experiment. *TRR* is a dummy indicating that a subjects is in the temporal role reversal treatment. *Part 2* $\times$ *TRR* and *Part 3* $\times$ *TRR* are interaction terms capturing the treatment effects for subjects in TRR compared to CR. All regressions include period fixed effects (Period Controls). *Panel 1*: regression limited to subjects who have a high endowment in part 1 (upper limit 40). *Panel 2*: regressions limited to subjects with a low endowment in part 2 (upper limit 20)

### Regressions including controls for past behavior

See Tables 16, 17 and 18.

**Table 16:** Linear regressions: share of contributions to the local and global public goods and private account—EE and IE treatment in part 1

DV	Own Local PG	Other Local PG	Global PG	Private
	(1)	(2)	(3)	(4)
Panel 1: IE (all subjects) and EE				
*IE*	0.507	0.130	1.157	$-$1.932
	(1.107)	(0.354)	(1.278)	(1.247)
*Lag own cont.*	0.790***	0.399***	0.844***	0.835***
	(0.0427)	(0.0934)	(0.0383)	(0.0318)
*Lag others–own cont.*	0.339***	$-$0.0320	0.317***	0.230***
	(0.0465)	(0.0369)	(0.0479)	(0.0442)
Constant	$-$0.0445	1.388***	3.483*	13.30***
	(1.555)	(0.499)	(1.780)	(2.118)
Observations	1824	1824	1824	1824
Period controls	Yes	Yes	Yes	Yes
F	70.65	7.229	105.5	215.4
$\hbox {R}^2$	0.302	0.231	0.455	0.456
Panel 2: IE (high and low endowment subjects) and EE				
*IE High endowment*	$-$1.864	$-$0.0495	$-$0.517	1.515
	(1.252)	(0.377)	(1.401)	(1.481)
*IE Low endowment*	2.878**	0.309	2.831**	$-$5.380***
	(1.433)	(0.427)	(1.401)	(1.503)
*Lag own cont.*	0.790***	0.399***	0.844***	0.835***
	(0.0427)	(0.0934)	(0.0383)	(0.0318)
*Lag others–own cont.*	0.353***	$-$0.0316	0.320***	0.244***
	(0.0464)	(0.0366)	(0.0477)	(0.0438)
Constant	$-$0.0445	1.388***	3.483*	13.30***
	(1.555)	(0.499)	(1.780)	(2.119)
Observations	1824	1824	1824	1824
Period controls	Yes	Yes	Yes	Yes
F	66.45	6.498	99.54	205.2
$\hbox {R}^2$	0.309	0.231	0.456	0.462

Linear regressions with period controls and cluster robust standard errors in parentheses. Standard errors are clustered at the global group level. *$p<0.1$, **$p<0.05$, ***$p<0.01$. Dependent variables: Share of endowment contributed to the own local public good [column (1)]; Share of endowment contributed to the local public good of the other group [column (2)]; Share of endowment contributed to the global public good [column (3)]; Share of endowment not contributed to any public good [column (4)]. *Lag own cont.* is a variable capturing a subject’s share of own contributions in the previous period. *Lag others–own cont.* is a variable capturing the difference between a subject’s own contribution (in percent of endowment) and the average contributions (in percent of endowment) within a group in the previous period. A negative value indicates that subjects contribute more than the average in a group. A positive value indicates that subjects contribute less than the average in a group. All regressions include period fixed effects (Period Controls). *Panel 1*: regressions comparing behavior of all subjects in the EE treatment (benchmark condition) with subjects in the EE treatment. *Panel 2*: regressions comparing behavior of all subjects in the EE treatment (benchmark condition) with subjects with a high endowment and subjects with a low endowment in the IE treatment. F-test for differences between *IE High endowment* and *IE Low endowment* in panel 2. F-test: Own Local PG: $\hbox {F}(1,75)=9.63$, $\hbox {p}<0.01$; Other Local PG: $\hbox {F}(1,75)=0.87$, $\hbox {p}= 0.36$; Global PG: $\hbox {F}(1,75)=8.52$, $\hbox {p}<0.01$; Private: $\hbox {F}(1,75)=17.74$, $\hbox {p}<0.01$

**Table 17:** Linear regressions: CR and TRR treatment—all subjects

DV	Own Local PG	Other Local PG	Global PG	Private
	(1)	(2)	(3)	(4)
*TRR*	0.380	0.254***	$-$0.0455	$-$0.465
	(0.345)	(0.0775)	(0.322)	(0.402)
*Part 2*	2.429***	0.252***	2.947***	$-$5.742***
	(0.559)	(0.0920)	(0.564)	(0.870)
*Part 3*	1.559***	0.124*	2.407***	$-$4.184***
	(0.552)	(0.0724)	(0.660)	(0.704)
*Part 2* $\times$ *TRR*	$-$0.122	$-$0.0889	0.158	0.195
	(0.470)	(0.124)	(0.448)	(0.495)
*Part 3* $\times$ *TRR*	$-$0.104	$-$0.208***	0.0801	0.210
	(0.295)	(0.0655)	(0.255)	(0.365)
*Lag own cont.*	0.760***	0.504***	0.841***	0.841***
	(0.0447)	(0.0770)	(0.0298)	(0.0282)
*Lag others–own cont.*	0.273***	$-$0.0179	0.294***	0.126***
	(0.0475)	(0.0270)	(0.0389)	(0.0317)
Constant	$-$0.149	$-$0.0723	0.807*	4.520***
	(0.472)	(0.0767)	(0.428)	(0.804)
Observations	4704	4704	4704	4704
Period controls	Yes	Yes	Yes	Yes
F	47.92	14.56	138.8	386.0
$\hbox {R}^2$	0.350	0.300	0.482	0.568

Linear (OLS) regressions with cluster robust standard errors in parentheses. Standard errors in parentheses. *$p<0.1$, **$p<0.05$, ***$p<0.01$. *Part 2* and *Part 3* are dummies capturing aggregate time trends in each part of the experiment. *TRR* is a dummy indicating that a subjects is in the temporal role reversal treatment. *Part 2* $\times$ *TRR* and *Part 3* $\times$ *TRR* are interaction terms capturing the treatment effects for subjects in TRR compared to CR. *Lag own cont.* is a variable capturing a subject’s own contributions in the previous period. *Lag others*−*own cont.* is a variable capturing the difference between a subject’s own contribution and the average contributions within a group in the previous period (average group contribution minus own contribution). A negative value indicates that subjects contribute more than the average in a group. A positive value indicates that subjects contribute less than the average in a group. All regressions include period fixed effects (Period Controls)

**Table 18:** Linear regressions: CR and TRR treatment—high and low endowment subjects

DV	Own Local PG	Other Local PG	Global PG	Private
	(1)	(2)	(3)	(4)
Panel 1: High endowment				
*TRR*	0.0820	0.264**	$-$0.548	0.201
	(0.570)	(0.113)	(0.523)	(0.733)
*Part 2*	4.266***	0.172	5.073***	$-$5.646***
	(0.942)	(0.184)	(1.056)	(1.636)
*Part 3*	2.381**	0.00612	3.561***	$-$2.177
	(0.990)	(0.148)	(1.099)	(1.337)
*Part 2* $\times$ *TRR*	0.334	0.0764	0.978	1.171
	(0.859)	(0.200)	(0.793)	(0.966)
*Part 3* $\times$ *TRR*	0.274	$-$0.214**	0.994**	1.254*
	(0.582)	(0.103)	(0.432)	(0.672)
*Lag own cont.*	0.914***	0.589***	0.941***	0.975***
	(0.0675)	(0.136)	(0.0432)	(0.0538)
*Lag others–own cont.*	0.466***	$-$0.0352	0.400***	0.416***
	(0.0955)	(0.0469)	(0.0792)	(0.0738)
Constant	$-$0.882	0.0117	0.522	6.437***
	(0.701)	(0.151)	(0.686)	(1.492)
Observations	2352	2352	2352	2352
Period controls	Yes	Yes	Yes	Yes
F	30.24	10.82	145.5	138.0
$\hbox {R}^2$	0.372	0.381	0.511	0.525
Panel 2: Low endowment				
*TRR*	0.655	0.238*	0.552	$-$1.316**
	(0.463)	(0.142)	(0.389)	(0.565)
*Part 2*	0.591	$-$0.105	1.616**	$-$5.245***
	(0.524)	(0.136)	(0.642)	(0.840)
*Part 3*	0.742	$-$0.248*	2.021***	$-$5.559***
	(0.584)	(0.125)	(0.700)	(0.825)
*Part 2* $\times$ *TRR*	$-$0.558	$-$0.296*	$-$0.771*	$-$0.205
	(0.575)	(0.151)	(0.454)	(0.703)
*Part 3* $\times$ *TRR*	$-$0.480	$-$0.143	$-$0.916**	$-$0.383
	(0.412)	(0.142)	(0.367)	(0.513)
*Lag own cont.*	0.618***	0.338***	0.701***	0.667***
	(0.0323)	(0.0633)	(0.0311)	(0.0275)
*Lag others–own cont.*	0.146***	0.0133	0.251***	0.201***
	(0.0318)	(0.0462)	(0.0312)	(0.0302)
Constant	0.551	0.312***	0.473	2.693***
	(0.507)	(0.113)	(0.406)	(0.686)
Observations	2352	2352	2352	2352
Period controls	Yes	Yes	Yes	Yes
F	55.80	13.90	57.85	143.9
$\hbox {R}^2$	0.311	0.165	0.406	0.417

Linear (OLS) regressions with cluster robust standard errors in parentheses. Standard errors in parentheses. *$p<0.1$, **$p<0.05$, ***$p<0.01$. *Part 2* and *Part 3* are dummies capturing aggregate time trends in each part of the experiment. *TRR* is a dummy indicating that a subjects is in the temporal role reversal treatment. *Part 2* $\times$ *TRR* and *Part 3* $\times$ *TRR* are interaction terms capturing the treatment effects for subjects in TRR compared to CR. *Lag own cont.* is a variable capturing a subject’s own contributions in the previous period. *Lag others–own cont.* is a variable capturing the difference between a subject’s own contribution and the average contributions within a group in the previous period (average group contribution minus own contribution). A negative value indicates that subjects contribute more than the average in a group. A positive value indicates that subjects contribute less than the average in a group. All regressions include period fixed effects (Period Controls). *Panel 1*: regression limited to subjects who have a high endowment in part 1. *Panel 2*: regressions limited to subjects with a low endowment in part 2

## Footnotes

1 Note, however, that Chan et al. (1999) reports no negative effects of inequality on overall giving levels.

2 Global (nationwide) measures preventing the spread of the Corona virus and free trade are other examples for more efficient global instead of local cooperation. Global free trade agreements benefit a vast number of countries and their population whereas bilateral tariff agreements are usually less efficient.

3 Applicants may use volunteering to signal familiarity with the situation of the less fortunate, which is presumed to shape pro-social attitudes and cooperation. See, e.g., the website https://www.kaptest.com/, which provides tips for high school students on how to increase chances with college admissions in the US.

4 See, e.g., the European ERASMUS program which appears “to strengthen students’ positive feelings towards the culture of the host country and also towards the home country, but not towards Europe as a whole” see Llurda et al. (2016), p. 340. Literature from psychology and sociology additionally points to potential benefits from gaining new perspectives. Participation in student exchange and nursing programs, for example, may change attitudes and beliefs and may positively affect future career prospects and personal development (e.g., Cziko and Lambert 1976; Hansel and Grove 1986; Carlson and Widaman 1988; Stroebe et al. 1988; Sowa 2002; Nunan 2006; Messer and Wolter 2007; Atkinson 2010; Zimmermann and Neyer 2013). Additionally, individuals who experienced extreme weather events are shown to undergo a change in their climate change beliefs and their willingness to contribute to climate protection (e.g., Dai et al. 2015).

5 An online search of the words “role reversal and perspective change” gives 328 million hits on google. Among them a multitude of websites explaining how role reversal is beneficial to gain new perspectives and how beneficial new experiences may be for oneself. Moreover, being able to at least hypothetically change the perspective is, for example, promoted to be an important leadership tool (see https://www.london.edu/think/power-of-perspective-taking).

6 See https://www.imdb.com/title/tt0086465/.

7 https://www.imdb.com/title/tt6713768/.

8 The novels, movies and TV show have a similar story-line: a rich and/or somewhat egoistic character has (through some circumstances) to experience how it is to be poor and/or work hard and suffer by experiencing the less fortunate perspective. After some time, the main character switches back to his original role and is wiser, more cooperative and/or generous than before.

9 Numerous voluntary exchange programs exist aiming at raising awareness for disadvantageous groups or the poor within society. Such programs usually suffer from selection and the assessment of their success is therefore hardly possible. Our findings suggest that such programs may be powerful even when eliminating self-selection. However, the strong form of temporary role reversal implemented in our lab experiment, i.e., forcing people into another position, is typically not feasible in exchange programs. Forced participation in social programs that raise awareness of living conditions of others, however, may be possible (e.g., a mandatory period of social service for everyone graduating from school).

10 Cacault and Grieder (2019), for example, additionally show that simple manipulations affect beliefs about abilities of in-group members.

11 In order to rematch groups, subjects are randomly grouped in matching groups of twelve. Hence, we have two global groups of six and four local groups of three in one matching group.

12 In the instructions, we denoted subjects in group A as player A and subjects in group B as player B. To indicate the perspective change, we highlight that subjects switched roles in part 2, i.e., subjects who are player A in part 1 are player B in part 2 and player A in part 3 again and vice versa.

13 Instructions (translated into English) can be found in the Appendix.

14 This procedure of not informing subjects about the content of subsequent parts is common in public goods experiments (see, e.g., Andreoni 1995; Andreoni and Croson 2008; Croson 1996, for literature on surprise restart public goods). See also Drouvelis and Grosskopf (2016) for public goods experiments in which subjects know the number of parts but receive information about each part separately.

15 Table 8 in Appendix A presents summary statistics for behavior in part 1 separated by CR and TRR. Non-parametric tests and parametric regressions confirm that no behavioral differences exist. An exemption are the small contributions to the public good of the other group. Two sided ranksum tests at the group level between TRR and CR: Share Own Local PG: $\hbox {N}=56$, $\hbox {p}=0.47$; Share Other Local PG: $\hbox {N}=56$, $\hbox {p}=0.04$; Share Global PG: $\hbox {N}=56$, $\hbox {p}=0.5$; Share private: $\hbox {N}=56$, $\hbox {p}=0.35$; Payoff: $\hbox {N}=56$, $\hbox {p}=0.63$.

16 Table 10 in Appendix A.1 presents regression results using absolute contributions as the dependent variable. Table 13 in Appendix A.2 presents results from Tobit regressions additionally accounting for the censored data structure (i.e., the maximum contribution of subjects was limited). Regressions in Table 16 in Appendix A.3 include controls for past behavior, which is, however, potentially correlated with the error component of the regression and thus violates the assumption of exogeneity (see also discussion in footnote 22). Further, we performed panel GLS regressions and nested random effects models to additionally check and confirm the robustness of our results. These regressions yield similar results and are available upon request.

17 Note that if not otherwise stated we use two-sided ranksum tests throughout the paper. We use global group averages as observations for non-parametric tests. This accounts for the fact that individual observations are not independent over time.

18 Table 9 in the Appendix further documents that subjects in the IE also contribute less to the global public good in absolute terms (8.8 points in the EE vs. 6.6 points in the IE; $\hbox {N}=76$, $\hbox {p}=0.052$).

19 Two sided ranksum tests at the global group level largely confirm the results presented in Table 3. *Share Global PG*: EE vs. IE High endowment: $\hbox {N} = 76$, $\hbox {p} = 0.01$; IE low endowment vs. IE High endowment: $\hbox {N}=112$, $\hbox {p}=0.04$; EE vs. IE Low endowment: $\hbox {N} = 76$, $\hbox {p} = 0.27$. *Share Own Local PG*: IE low endowment vs. IE High endowment: $\hbox {N} = 112$, $\hbox {p} < 0.01$; EE vs. IE high endowment: $\hbox {N} = 76$, $\hbox {p} = 0.26$; EE vs. IE low endowment: $\hbox {N}=76$, $\hbox {p}=0.09$. *Payoffs*: EE vs. IE high endowment: $\hbox {N} = 76$, $\hbox {p}< 0.01$; IE low endowment vs. IE High endowment: $\hbox {N} = 112$, $\hbox {p} < 0.01$; EE vs. IE low endowment: $\hbox {N} = 76$, $\hbox {p} < 0.01$; EE vs. IE: $\hbox {N} = 76$, $\hbox {p} = 0.04$.

20 As a sidenote, this difference becomes even more pronounced in part 2 and especially part 3 when we compare EE with CR, as subjects with a low endowment in CR significantly increase the share of their endowment contributed to the global public good relative to EE, while we do not see any significant change in this difference for high endowment subjects. Estimation results are omitted for reasons of brevity, but are available upon request.

21 This approach also controls for the small yet insignificant differences in contribution decisions between CR and TRR that exist in part 1 (see, e.g., Fig. 4).

22 For robustness, we present regressions using absolute contributions as the dependent variable in Tables 11 and 12 in Appendix A.1. We further present Tobit regressions accounting for censored data (i.e., a subject’s contribution was limited to his/her endowment) in Tables 14 and 15 in Appendix A.2. Following (Chaudhuri et al. 2006), we additionally present regressions including controls for own past behavior (lagged own contributions) and the difference of between own and the average group contribution in the previous period (lag average group contribution − lag own contribution) in Tables 17 and 18 in Appendix A.3. The coefficients for past behavior are in line with results from previous studies and unsurprisingly positive and highly significant (see, e.g., Chaudhuri et al. 2006; Croson et al. 2005; Frey and Meier 2004; Sefton et al. 2007). Overall, regressions show similar results to the ones presented in the main body of the paper. We also ran panel GLS and nested random effects regressions to account for the panel structure. Results are robust and available upon request. Please note, that variables for past behavior are usually correlated with the error component and thus violate the assumption of strict exogeneity of the explanatory variables and may therefore result in biased and inconsistent estimators of the corresponding variables. For a consistent and unbiased estimation of the effects of own and others’ past behavior, approaches for dynamic models (Arellano and Bond 1991) need to be employed. In this case information about behavior should not be disclosed. Our design is unfortunately not suitable for these approaches as we considered three parts in which subjects changed between groups and information about contributions of others is a key element of the design in all treatments.

23 Two sided ranksum tests (at the global group level) for differences in part 3 behavior confirm our regression results. *Share Own Local PG:* High endowment: TRR (9.6%) vs. CR (8.6%) : $\hbox {N}=56$, $\hbox {p}=0.14$; Low endowment: TRR (16.4%) vs. CR (12.8%) : $\hbox {N}=56$, $\hbox {p}=0.28$; *Share Other Local PG:* High endowment: TRR (0.67%) vs. CR (0.4%) : $\hbox {N}=56$, $\hbox {p}=0.57$; Low endowment: TRR (1.7%) vs. CR (0.7%) : $\hbox {N}=56$, $\hbox {p}=0.03$; *Share Global PG:* High endowment: TRR (14.79%) vs. CR (11.68%): $\hbox {N}=56$, $\hbox {p}=0.1$; Low endowment: TRR (17.71%) vs. CR (17.35%): $\hbox {N}=56$, $\hbox {p}=0.66$. Note, however, when including controls for past behavior the effect of role reversal on subjects with a low endowment becomes marginally significant when considering contributions to the global public good (see Table 18 in Appendix A.3) Please note, however, the discussion in footnote 22, such that these estimators are potentially biased.

24 Two sided ranksum tests (at the global group level) for payoff differences between CR and TRR in part 3: All subjects CR (36.05 points) vs. TR (37.13 points): $\hbox {N}=56$, $\hbox {p}=0.2$; High endowment: CR (45.62 points) vs. TRR (46.18 points): $\hbox {N}=56$, $\hbox {p}=0.41$; Low endowment: CR (26.48 points) vs. TRR (28.08 points): $\hbox {N}=56$, $\hbox {p}=0.1$. Two sided ranksum tests (at the global group level) confirm the persistence of the endowment inequality between the CR/TRR and EE in part 3: All subjects: CR (36.05 points) vs. EE (36.03 points): $\hbox {N}= 48$, $\hbox {p}=0.35$: High endowment CR (45.62 points) vs. EE (36.03 points): $\hbox {N}=48$, $\hbox {p}<0.01$; Low endowment CR (26.48 points) vs. EE (36.03 points): $\hbox {N}=48$, $\hbox {p}<0.01$; All subjects: TRR (37.13 points) vs. EE (36.03 points): $\hbox {N}= 48$, $\hbox {p}=0.51$: High endowment TRR (46.18 points) vs. EE (36.03 points): $\hbox {N}=48$, $\hbox {p}<0.01$; Low endowment TRR (28.08 points) vs. EE (36.03 points): $\hbox {N}=48$, $\hbox {p}<0.01$.

25 Students subjects self report to have on average 615 Euro (after expenses) at their disposal outside the laboratory. Even with this income, economic consequences from having a high or a low endowment in the study are limited (3 Euro vs. 1.50 Euro). We find moderate effects even for the small stakes involved in the laboratory. Real world cooperation problems naturally involves more severe inequality levels.

26 In addition, numerous voluntary exchange programs exist aiming at raising awareness for disadvantageous groups or the poor within society. Such programs usually suffer from selection and the assessment of their success is therefore hardly possible. Our findings suggest that such programs may be powerful even when eliminating self-selection.
