# Supplementary material for: Inequality, role reversal and cooperation in multiple group membership settings
Source: Exp Econ. 2021 Mar 10;25(1):68–110. doi: 10.1007/s10683-021-09705-y (PMC7945615; doi:10.1007/s10683-021-09705-y)
Supplement: Supplementary file 1 — Electronic supplementary material 1 (ZIP 3076 kb) [file 10683_2021_9705_MOESM1_ESM.zip › appendix section 5/Instructions for online appendix/Instructions_General.pdf]

General explanations for participants

Welcome to the experimental laboratory!

Today, you will participate in an economic experiment. The amount of money you earn depends on your own decisions and the decisions of other participants. Therefore, it is important that you carefully read the following instructions.

The instructions you have received from us are for your private information. **During the experiment, communication is absolutely prohibited.** If you have any questions, please contact us by raising your hands. An experimenter will come to you and answer your questions. Failure to comply with the rules will result in exclusion from the experiment and all payments.

The decisions you make during the experiment are anonymous. Only the experimenter knows your identity but your decisions cannot be assigned to your identity.

For your participation in the experiment, you will receive a show up fee of 4 Euro. The additional payment depends on your decisions and the decisions of other participants. Your payment during the experiment will be calculated in points. The total number of points obtained will be converted into Euros and then paid to you in cash. For the conversion, the following exchange rate applies:

20 Points = 1.50 Euro.

The experiment consists of **3 parts** and each part is divided into 5 periods. In total, there are 15 periods. You will first receive a brief explanation for Part 1 (Periods 1 to 5) and make decisions that you enter in the computer. After Part 1, you will receive a brief explanation for Part 2 (Periods 6 to 10). After Part 2 you will receive a brief explanation for Part 3 (Periods 11 to 15) and make your decisions again. Finally, we ask you to answer some general questions.

At the end of the experiment you will receive your payment. **One period of each part will be randomly selected for your payment**. These 3 randomly selected periods are the same for all participants. The earned points from these 3 periods will be converted into Euros and added to your show up fee of 4 Euro. Your receive your payment in cash. All payouts will be made separately so that the other participants will not be able to obtain information about your earnings.

Do you have any questions?
