# Supplementary material for: Inequality, role reversal and cooperation in multiple group membership settings
Source: Exp Econ. 2021 Mar 10;25(1):68–110. doi: 10.1007/s10683-021-09705-y (PMC7945615; doi:10.1007/s10683-021-09705-y)
Supplement: Supplementary file 1 — Electronic supplementary material 1 (ZIP 3076 kb) [file 10683_2021_9705_MOESM1_ESM.zip › appendix section 5/Instructions for online appendix/Instructions_Part1_T3_page1.pdf]

Explanations for Part 1

Part 1 of the experiment consists of 5 periods, in which you always play the same game. All participants are divided in groups of six. Thus, you will interact with 5 other participants. Please note that all your decisions and the decisions of the other participants are anonymous. The other group members will not be able to observe your decisions in detail.

At the end of the experiment, you will receive **your payment in Euro from 3 out of the 15 periods of the experiment**. Therefore, one period of each part will be randomly selected for your payment. Hence, any period can be payoff relevant.

The following pages describe in detail the procedure of the first part of the experiment.

Each player is facing the same decision problem. Your task (and the task of the other participants) is to decide on the distribution of points between your own account and three other accounts (Account A, Account B, and Account AB).

At the beginning the experiment, **three players in a group will be randomly assigned as type 'A' and the remaining, three in a group are type 'B' players**. This type assignment will be the same in all 5 periods.

At the beginning of each period, all players A receive **30 points** and all players B receive **30 points** on their **personal accounts**.

Each player must decide, how many points she wants to transfer to the accounts A, B or AB. The sum of these transfers must not exceed the number of points on the personal account. Thus, the transfers of player A can range from 0 to 40 and of the transfers of player B from 0 to 20 (all transfers in integers).

All players can transfer points to the accounts A, B, and AB. Transfers to the accounts A, B, or AB do have different impacts on the earnings depending on the type of the player. Players A benefit from transfers to account A and account AB. Players B benefit from transfers to account B and account AB. Players A **do not** receive earnings from account B and players B **do not** receive earnings from account A.

The **payoff for a player A** is calculated as follows:
