# Supplementary material for: Inequality, role reversal and cooperation in multiple group membership settings
Source: Exp Econ. 2021 Mar 10;25(1):68–110. doi: 10.1007/s10683-021-09705-y (PMC7945615; doi:10.1007/s10683-021-09705-y)
Supplement: Supplementary file 1 — Electronic supplementary material 1 (ZIP 3076 kb) [file 10683_2021_9705_MOESM1_ESM.zip › appendix section 5/Instructions for online appendix/Instructions_Part2_T2-3.pdf]

Explanations for Part 2

Part 2 of the experiment also consists of **5 periods**, in which you play the same game as in Part 1. You will be randomly divided into new groups. You and 2 other players of the same type (A or B) from Part 1will be reassigned to 3 other players of the other type to form a new group.

If you were a player A in Part 1 you will stay together with the other former players A from Part 1. Your new group consists additionally of 3 players of another group who were players B before.

If you were a player B in Part 1 you will stay together with the other former players B from Part 1. Your new group additionally consists of 3 players of another group who were players A before.

Do you have any questions?
