# Supplementary material for: Inequality, role reversal and cooperation in multiple group membership settings
Source: Exp Econ. 2021 Mar 10;25(1):68–110. doi: 10.1007/s10683-021-09705-y (PMC7945615; doi:10.1007/s10683-021-09705-y)
Supplement: Supplementary file 1 — Electronic supplementary material 1 (ZIP 3076 kb) [file 10683_2021_9705_MOESM1_ESM.zip › appendix section 5/Instructions for online appendix/Instructions_Part3_T1.pdf]

Explanations for Part 3

Part 3 of the experiment also consists of 5 periods, in which you will play the same game as in Part 1 and Part 2. In this part of the game, you will play with the same group composition as in Part 1 of the game. You and all other players will be of the same type (Player A or Player B) as in Part 1 of the game.

Do you have any questions?
