# Supplementary material for: Inequality, role reversal and cooperation in multiple group membership settings
Source: Exp Econ. 2021 Mar 10;25(1):68–110. doi: 10.1007/s10683-021-09705-y (PMC7945615; doi:10.1007/s10683-021-09705-y)
Supplement: Supplementary file 1 — Electronic supplementary material 1 (ZIP 3076 kb) [file 10683_2021_9705_MOESM1_ESM.zip › appendix section 5/instructions.pdf]

A Appendix

A.5 Instructions

General instructions for all participants

General explanations for participants

Welcome to the experimental laboratory!

Today, you will participate in an economic experiment. The amount of money you earn depends on your own decisions and the decisions of other participants. Therefore, it is important that you carefully read the following instructions.

The instructions you have received from us are for your private information. **During the experiment, communication is absolutely prohibited.** If you have any questions, please contact us by raising your hands. An experimenter will come to you and answer your questions. Failure to comply with the rules will result in exclusion from the experiment and all payments.

The decisions you make during the experiment are anonymous. Only the experimenter knows your identity but your decisions cannot be assigned to your identity.

For your participation in the experiment, you will receive a show up fee of 4 Euro. The additional payment depends on your decisions and the decisions of other participants. Your payment during the experiment will be calculated in points. The total number of points obtained will be converted into Euros and then paid to you in cash. For the conversion, the following exchange rate applies:

20 Points = 1.50 Euro.

The experiment consists of **3 parts** and each part is divided into 5 periods. In total, there are 15 periods. You will first receive a brief explanation for Part 1 (Periods 1 to 5) and make decisions that you enter in the computer. After Part 1, you will receive a brief explanation for Part 2 (Periods 6 to 10). After Part 2 you will receive a brief explanation for Part 3 (Periods 11 to 15) and make your decisions again. Finally, we ask you to answer some general questions.

At the end of the experiment you will receive your payment. **One period of each part will be randomly selected for your payment**. These 3 randomly selected periods are the same for all participants. The earned points from these 3 periods will be converted into Euros and added to your show up fee of 4 Euro. Your receive your payment in cash. All payouts will be made separately so that the other participants will not be able to obtain information about your earnings.

Do you have any questions?

Part 1: Instructions for participants in the CR and TRR treatment

Explanations for Part 1

Part 1 of the experiment consists of 5 periods, in which you always play the same game. All participants are divided in groups of six. Thus, you will interact with 5 other participants. Please note that all your decisions and the decisions of the other participants are anonymous. The other group members will not be able to observe your decisions in detail.

At the end of the experiment, you will receive **your payment in Euro from 3 out of the 15 periods of the experiment**. Therefore, one period of each part will be randomly selected for your payment. Hence, any period can be payoff relevant.

The following pages describe in detail the procedure of the first part of the experiment.

Each player is facing the same decision problem. Your task (and the task of the other participants) is to decide on the distribution of points between your own account and three other accounts (Account A, Account B, and Account AB).

At the beginning the experiment, **three players in a group will be randomly assigned as type 'A' and the remaining, three in a group are type 'B' players**. This type assignment will be the same in all 5 periods.

At the beginning of each period, all players A receive **40 points** and all players B receive **20 points** on their **personal accounts**.

Each player must decide, how many points she wants to transfer to the accounts A, B or AB. The sum of these transfers must not exceed the number of points on the personal account. Thus, the transfers of player A can range from 0 to 40 and of the transfers of player B from 0 to 20 (all transfers in integers).

All players can transfer points to the accounts A, B, and AB. Transfers to the accounts A, B, or AB do have different impacts on the earnings depending on the type of the player. Players A benefit from transfers to account A and account AB. Players B benefit from transfers to account B and account AB. Players A **do not** receive earnings from account B and players B **do not** receive earnings from account A.

The **payoff for a player A** is calculated as follows:

(A1) the points the player keeps on her account,

(A2) plus the **income from account A**, which is calculated as follows:

Income of a player A from account A =   0,5 *times* total sum of points on account A

(A3) plus the **income from account AB**, which is calculated as follows:

Income of a player A from account AB =   0,35 *times* total sum of points on account AB

The **payoff for a player B** is calculated as follows:

(B1) the points the player keeps on her account

(B2) plus the **income from account B**, which is calculated as follows:

Income of a player B from account B =   0,5 *times* total sum of points on account A

(B3) plus the **income from account AB**, which is calculated as follows:

Income of a player B from account AB =   0,35 *times* total sum of points on account AB

The **income of player A** amounts to

40 – Sum of the player's transfers to the accounts A, B und AB
+ 0,5 × (total sum of points on account A)
+ 0 × (total sum of points on account B)
+ 0,35 × (total sum of points on account AB).

The **income of player B** amounts to

40 – Sum of the player's transfers to the accounts A, B und AB
+ 0 × (total sum of points on account A)
+ 0,5 × (total sum of points on account B)
+ 0,35 × (total sum of points on account AB).

If, for example, the total sum of points transferred to account AB amounts to 40, you and all other players in your group receive an income of 0,35*40=14 points from this account.

If, for example, the total sum of points transferred to account A amounts to 10, all **three players A** receive an income of 0,5*10=5 points from this account. Players B receive no income from account A.

If, for example, the total sum of points transferred to account B amounts to 14, all **three players B** receive in each case an income of 0,5*14=7 points from this account. Players A receive no income from account A.

Examples for the incomes of players A and players B

Example 1: The first player A transfers 0 points to the accounts A, B, and AB, the other players A each transfer 20 points to the account A and 20 points to the account AB. The first player B transfers 0 points to accounts A, B, and AB, the other players B transfer 10 points to the account B and 10 points to the account AB.

- Income of the first player A:
  40 – 0 + 0.5 x 40 (Account A) + 0 x 20 (Account B) + 0.35 x 60 (Account AB) = 81 points
- Income of the other players A:
  40 – 40 + 0.5 x 40 (Account A) + 0 x 20 (Account B) + 0.35 x 60 (Account AB) = 41 points
- Income of the first player B:
  20 – 0 + 0 x 40 (Account A) + 0.5 x 20 (Account B) + 0.35 x 60 (Account AB) = 51 points.
- Income of the other players B:
  20 – 20 + 0 x 40 (Account A) + 0.5 x 20 (Account B) + 0.35 x 60 (Account AB) = 31 points.

Example 2: 3 players A transfer 40 points to accounts AB and 3 players B transfer 20 points to account AB.

- Income of a player A:
  40 – 40 + 0.5 x 0 (Account A) + 0 x 0 (Account B) + 0.35 x 180 (Account AB) = 63 points
- Income of a player B:
  20 – 20 + 0 x 0 (Account A) + 0.5 x 0 (Account B) + 0.35 x 180 (Account AB) = 63 points

Example 3: 3 players A transfer 40 points to account A and 3 players B transfer 20 points to account B.

- Income of a player A:
  40 – 40 + 0.5 x 120 (Account A) + 0 x 60 (Account B) + 0.35 x 0 (Account AB) = 60 points
- Income of a player B:
  20 – 20 + 0 x 120 (Account A) + 0.5 x 60 (Account B) + 0.35 x 0 (Account AB) = 30 points

Example 4: 3 players A transfer 40 points to account B and 3 players B transfer 20 points to account A.

- Income of a player A:
  40 – 40 + 0.5 x 60 (Account A) + 0 x 120 (Account B) + 0.35 x 0 (Account AB) = 30 points
- Income of a player B:
  20 – 20 + 0 x 60 (Account A) + 0.5 x 120 (Account B) + 0.35 x 0 (Account AB) = 60 points

Example 5: 3 players A transfer 0 points to accounts A, B, or AB and 3 players B transfer 0 points to accounts A, B, or AB.

- Income of a player A:
  40 – 0 + 0.5 x 0 (Account A) + 0.5 x 0 (Account B) + 0.35 x 0 (Account AB) = 40 points
- Income of a player B:
  20 – 0 + 0.5 x 0 (Account A) + 0.5 x 0 (Account B) + 0.35 x 0 (Account AB) = 20 points

Information about the experimental procedure

At the beginning of each period an **entry screen** appears. At the top left of the screen you will see the **period number.** At the top right is a **time display in seconds**, which counts down the remaining time to make your decision.

Entry screen for player A:

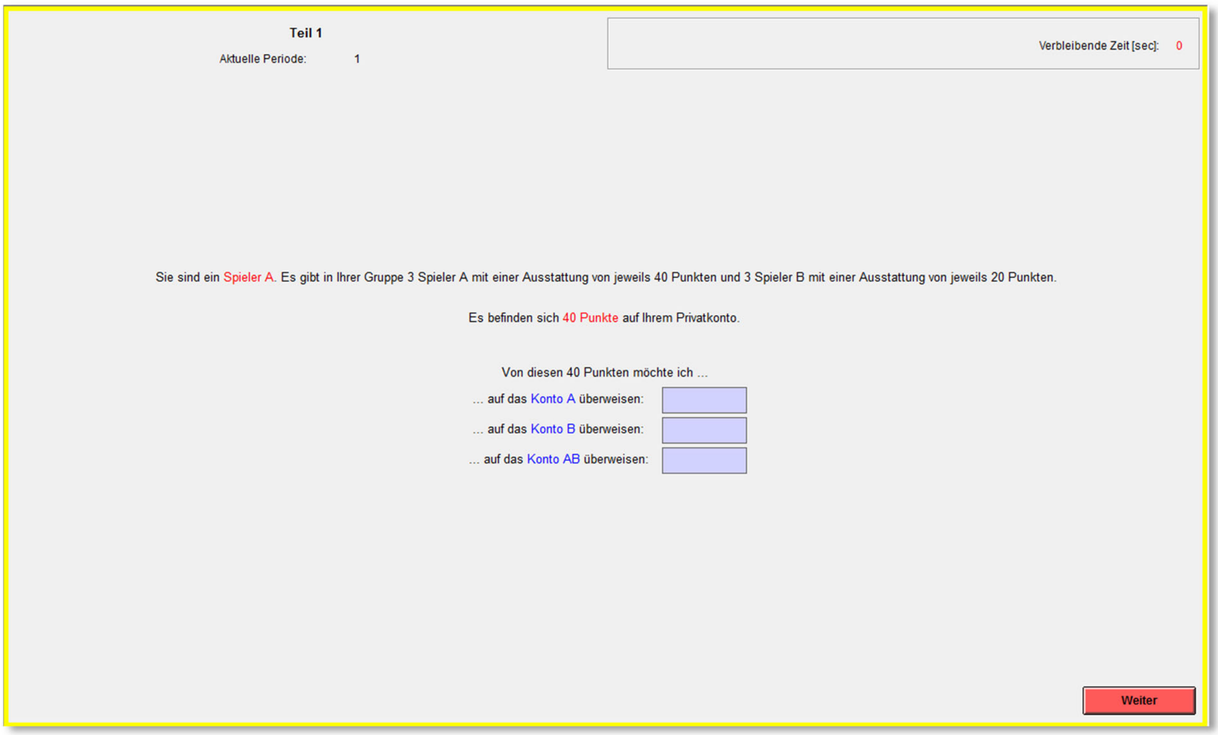

Entry screen for player B:

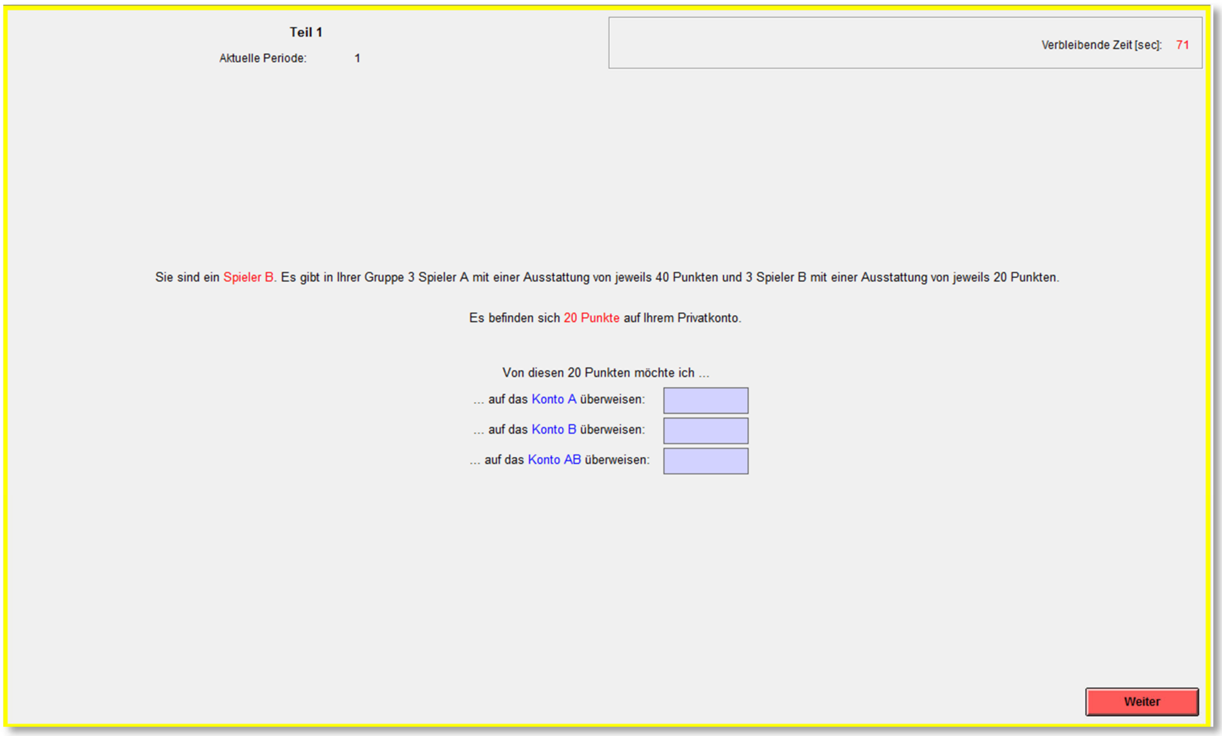

You decide on the transfers to accounts A, B, and AB by inserting an integer between 0 and 40 (Player A) or between 0 and 20 (Player B) in the entry fields. These fields can be activated with a mouse click. The sum of all transfers cannot exceed your endowment of 40 points (Player A) or 20 points (Player B).

After you have entered your decision, you proceed with the **continue button**. As soon as you clicked on "Continue" you cannot revise your decision for this period anymore.

After all group members have made their decisions, you will receive information in the account overview screen about the total points on the accounts A, B, and AB as well as on your personal account. Furthermore, you will receive information about your final income for that period.

The account overview screen:

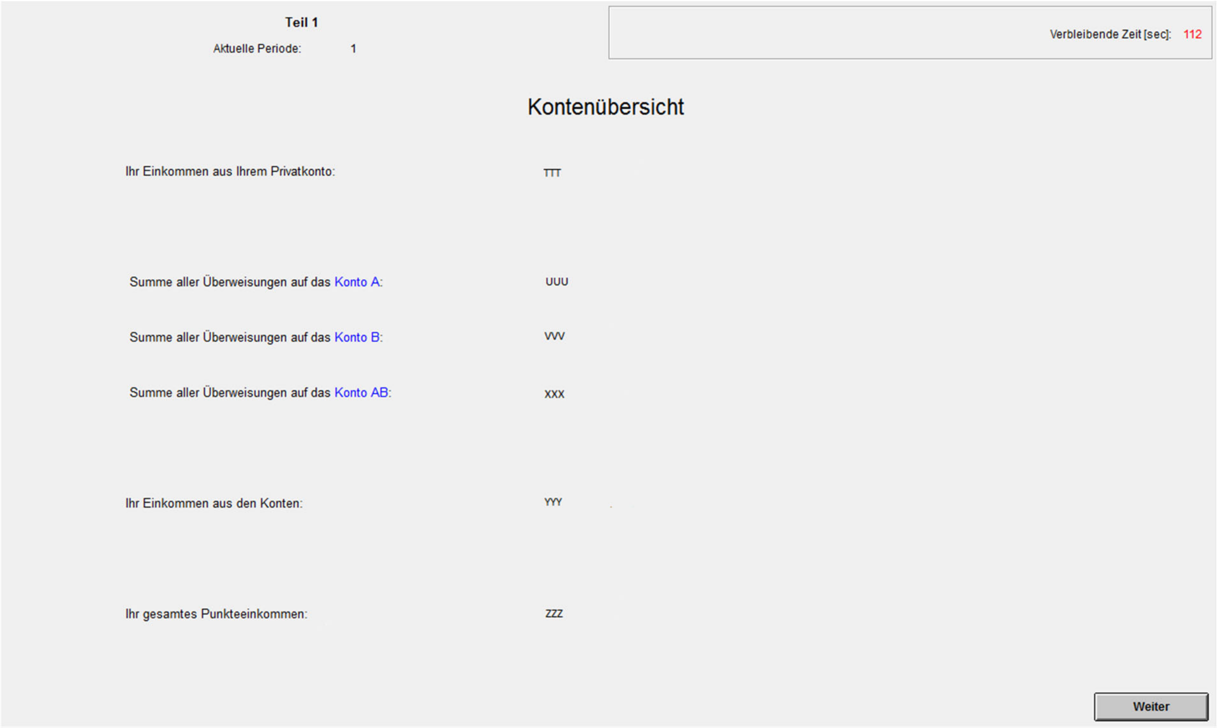

Before the experiment starts, all participants have to solve some test questions. These questions familiarise you with the rules of the experiment.

Do you have any questions about the experiment?

Part 1: Instructions for participants in the EE treatment

Explanations for Part 1

Part 1 of the experiment consists of 5 periods, in which you always play the same game. All participants are divided in groups of six. Thus, you will interact with 5 other participants. Please note that all your decisions and the decisions of the other participants are anonymous. The other group members will not be able to observe your decisions in detail.

At the end of the experiment, you will receive **your payment in Euro from 3 out of the 15 periods of the experiment**. Therefore, one period of each part will be randomly selected for your payment. Hence, any period can be payoff relevant.

The following pages describe in detail the procedure of the first part of the experiment.

Each player is facing the same decision problem. Your task (and the task of the other participants) is to decide on the distribution of points between your own account and three other accounts (Account A, Account B, and Account AB).

At the beginning the experiment, **three players in a group will be randomly assigned as type 'A' and the remaining, three in a group are type 'B' players**. This type assignment will be the same in all 5 periods.

At the beginning of each period, all players A receive **30 points** and all players B receive **30 points** on their **personal accounts**.

Each player must decide, how many points she wants to transfer to the accounts A, B or AB. The sum of these transfers must not exceed the number of points on the personal account. Thus, the transfers of player A can range from 0 to 40 and of the transfers of player B from 0 to 20 (all transfers in integers).

All players can transfer points to the accounts A, B, and AB. Transfers to the accounts A, B, or AB do have different impacts on the earnings depending on the type of the player. Players A benefit from transfers to account A and account AB. Players B benefit from transfers to account B and account AB. Players A **do not** receive earnings from account B and players B **do not** receive earnings from account A.

The **payoff for a player A** is calculated as follows:

(A1) the points the player keeps on her account,

(A2) plus the **income from account A**, which is calculated as follows:

Income of a player A from account A =<br>0,5 *times* total sum of points on account A

(A3) plus the **income from account AB**, which is calculated as follows:

Income of a player A from account AB =<br>0,35 *times* total sum of points on account AB

The **payoff for a player B** is calculated as follows:

(B1) the points the player keeps on her account

(B2) plus the **income from account B**, which is calculated as follows:

Income of a player B from account B =<br>0,5 *times* total sum of points on account A

(B3) plus the **income from account AB**, that is calculated as follows:

Income of a player B from account AB =<br>0,35 *times* total sum of points on account AB

The **income of player A** amounts to

30 – Sum of the player's transfers to the accounts A, B und AB
+ 0,5 × (total sum of points on account A)
+ 0 × (total sum of points on account B)
+ 0,35 × (total sum of points on account AB).

The **income of player B** amounts to

30 – Sum of the players transfer's to the accounts A, B und AB
+ 0 × (total sum of points on account A)
+ 0,5 × (total sum of points on account B)
+ 0,35 × (total sum of points on account AB).

If, for example, the total sum of points transferred to account AB amounts to 40, you and all other players in your group receive an income of 0,35*40=14 points from this account.

If, for example, the total sum of points transferred to account A amounts to 10, all **three players A** receive an income of 0,5*10=5 points from this account. Players B receive no income from account A.

If, for example, the total sum of points transferred to account B amounts to 14, all **three players B** receive in each case an income of 0,5*14=7 points from this account. Players A receive no income from account A.

Examples for the incomes of players A and players B

Example 1: The first player A transfers 0 points to the accounts A, B, and AB, the other players A each transfer 15 points to the account A and 15 points to the account AB. The first player B transfers 0 points to accounts A, B, and AB, the other players B transfer 15 points to the account B and 15 points to the account AB.

- Income of the first player A:
  30 – 0 + 0.5 x 30 (Account A) + 0 x 30 (Account B) + 0.35 x 60 (Account AB) = 66 points
- Income of the other players A:
  30 – 30 + 0.5 x 30 (Account A) + 0 x 20 (Account B) + 0.35 x 60 (Account AB) = 36 points
- Income of the first player B:
  30 – 0 + 0 x 30 (Account A) + 0.5 x 30 (Account B) + 0.35 x 60 (Account AB) = 66 points.
- Income of the other players B:
  30 – 30 + 0 x 30 (Account A) + 0.5 x 20 (Account B) + 0.35 x 60 (Account AB) = 36 points.

Example 2: 3 players A transfer 30 points to accounts AB and 3 players B transfer 30 points to account AB.

- Income of a player A:
  30 – 30 + 0.5 x 0 (Account A) + 0 x 0 (Account B) + 0.35 x 180 (Account AB) = 63 points
- Income of a player B:
  30 – 30 + 0 x 0 (Account A) + 0.5 x 0 (Account B) + 0.35 x 180 (Account AB) = 63 points

Example 3: 3 players A transfer 30 points to account A and 3 players B transfer 30 points to account B.

- Income of a player A:
  30 – 30 + 0.5 x 90 (Account A) + 0 x 90 (Account B) + 0.35 x 0 (Account AB) = 45 points
- Income of a player B:
  30 – 30 + 0 x 90 (Account A) + 0.5 x 90 (Account B) + 0.35 x 0 (Account AB) = 45 points

Example 4: 3 players A transfer 30 points to account B and 3 players B transfer 30 points to account A.

- Income of a player A:
  30 – 30 + 0.5 x 90 (Account A) + 0 x 90 (Account B) + 0.35 x 0 (Account AB) = 45 points
- Income of a player B:
  30 – 30 + 0 x 90 (Account A) + 0.5 x 90 (Account B) + 0.35 x 0 (Account AB) = 45 points

Example 5: 3 players A transfer 0 points to accounts A, B, or AB and 3 players B transfer 0 points to accounts A, B, or AB.

- Income of a player A:
  30 – 0 + 0.5 x 0 (Account A) + 0.5 x 0 (Account B) + 0.35 x 0 (Account AB) = 30 points
- Income of a player B:
  30 – 0 + 0.5 x 0 (Account A) + 0.5 x 0 (Account B) + 0.35 x 0 (Account AB) = 30 points

Information about the experimental procedure

At the beginning of each period an **entry screen** appears**.** At the top left of the screen you will see the **period number.** At the top right is a **time display in seconds**, which counts down the remaining time to make your decision.

Entry screen for player A:

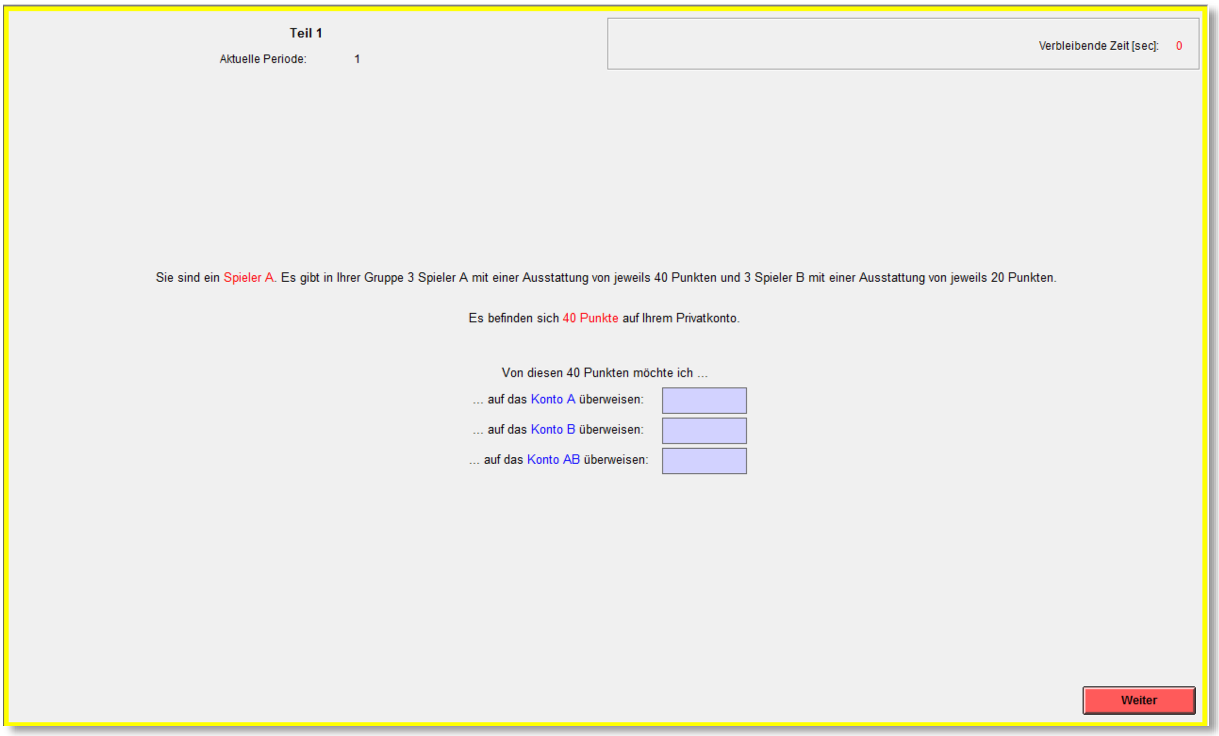

Entry screen for player B:

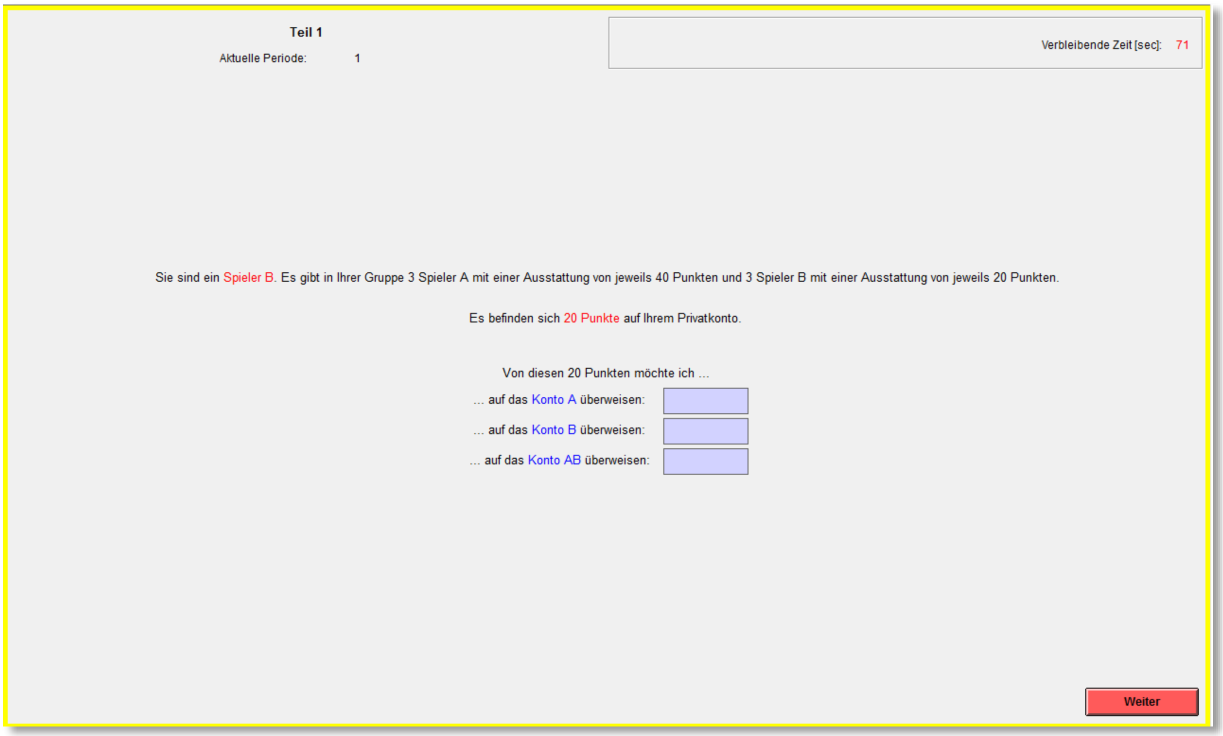

You decide on the transfers to accounts A, B, and AB by inserting an integer between 0 and 30 (Player A) or between 0 and 30 (Player B) in the entry fields. These fields can be activated with a mouse click. The sum of all transfers cannot exceed your endowment of 30 points (Player A) or 30 points (Player B).

After you have entered your decision, you proceed with the **continue button**. As soon as you clicked on "Continue" you cannot revise your decision for this period anymore.

After all group members have made their decisions, you will receive information in the account overview screen about the total points on the accounts A, B, and AB as well as on your personal account. Furthermore, you will receive information about your final income for that period.

The account overview screen:

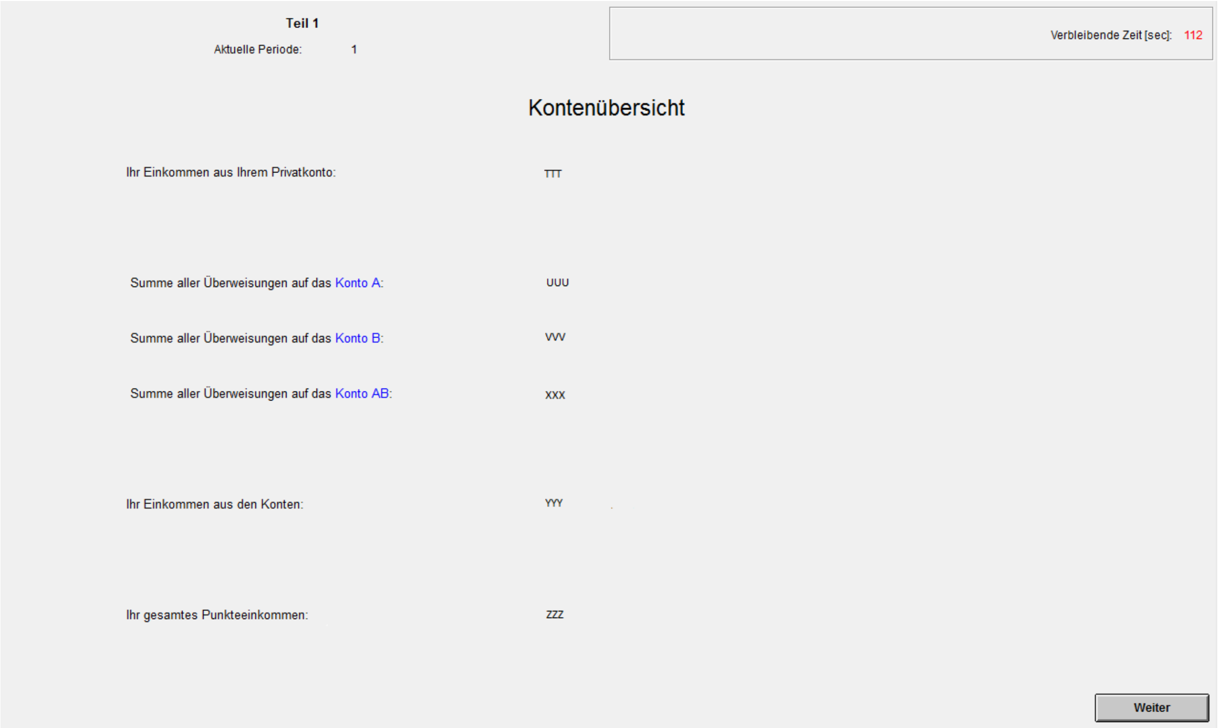

Before the experiment starts, all participants have to solve some test questions. These questions familiarise you with the rules of the experiment.

Do you have any questions about the experiment?

Part 2: Instructions for participants in the CR and EE treatment

Explanations for Part 2

Part 2 of the experiment also consists of **5 periods**, in which you play the same game as in Part 1. You will be randomly divided into new groups. You and 2 other players of the same type (A or B) from Part 1will be reassigned to 3 other players of the other type to form a new group.

If you were a player A in Part 1 you will stay together with the other former players A from Part 1. Your new group consists additionally of 3 players of another group who were players B before.

If you were a player B in Part 1 you will stay together with the other former players B from Part 1. Your new group additionally consists of 3 players of another group who were players A before.

Do you have any questions?

Part 2: Instructions for participants in the TRR treatment

Explanations for Part 2

Part 2 of the experiment also consists of **5 periods**, in which you play the same game as in Part 1. You will be randomly divided into new groups. You and 2 other players of the same type (A or B) from Part 1will be reassigned to 3 other players of the other type to form a new group.

If you were a player A in Part 1 you will stay together with the other former players A from Part 1. Your new group consists additionally of 3 players of another group who were players B before.

If you were a player B in Part 1 you will stay together with the other former players B from Part 1. Your new group additionally consists of 3 players of another group who were players A before.

Additionally, you are a player of a different type in this part of the game. If you were a player A in Part 1, you are now a player B in Part 2. If you were a player B in Part 1, you are now a player A in Part 2. The same applies for all other participants of the game.

This means that if you as player A received 40 points on your personal account, you will now receive 20 points as player B. If you as player B received 20 points on your personal account, you will now receive 40 points as player A. The same applies for all other participants.

Do you have any questions?

Part 3: Instructions for all participants

Explanations for Part 3

Part 3 of the experiment also consists of 5 periods, in which you will play the same game as in Part 1 and Part 2. In this part of the game, you will play with the same group composition as in Part 1 of the game. You and all other players will be of the same type (Player A or Player B) as in Part 1 of the game.

Do you have any questions?
