# Supplementary figures and images for: Inequality, role reversal and cooperation in multiple group membership settings
Source: Exp Econ. 2021 Mar 10;25(1):68–110. doi: 10.1007/s10683-021-09705-y (PMC7945615; doi:10.1007/s10683-021-09705-y)

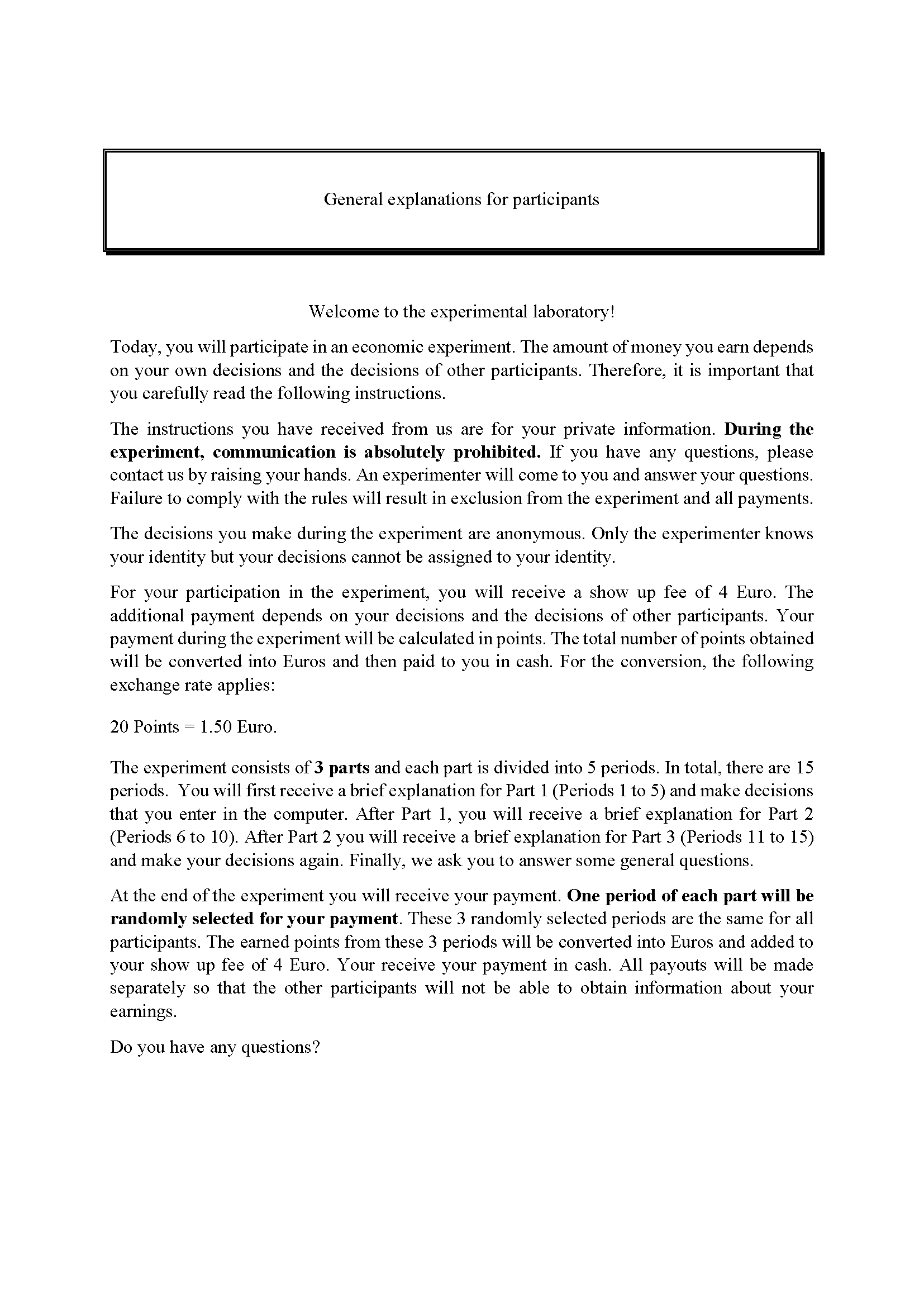

Supplement: Supplementary file 1 — Electronic supplementary material 1 (ZIP 3076 kb) [file 10683_2021_9705_MOESM1_ESM.zip › appendix section 5/Instructions for online appendix/Instructions_General.png]

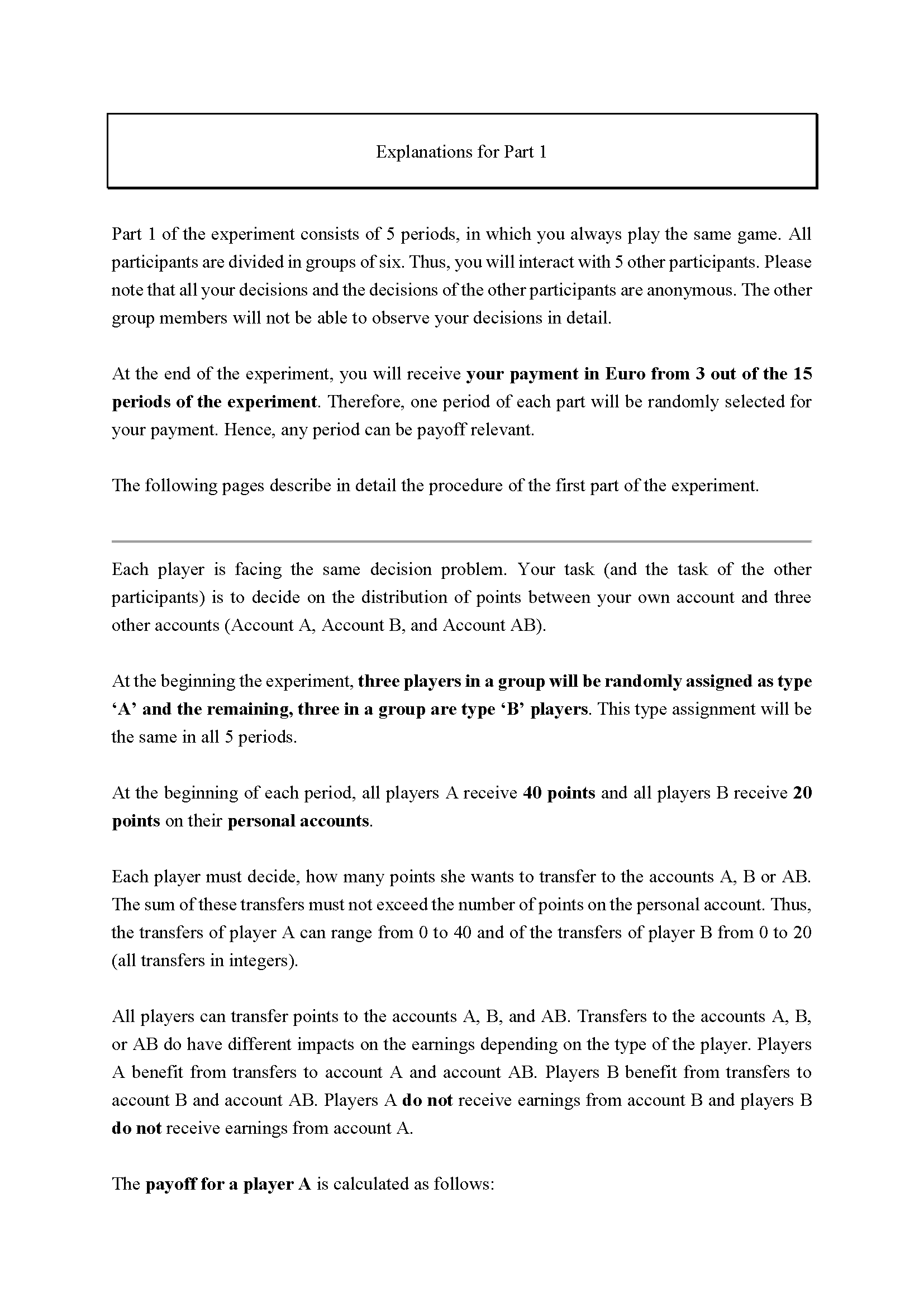

Supplement: Supplementary file 1 — Electronic supplementary material 1 (ZIP 3076 kb) [file 10683_2021_9705_MOESM1_ESM.zip › appendix section 5/Instructions for online appendix/Instructions_Part1_T1-2-4_Page_1.png]

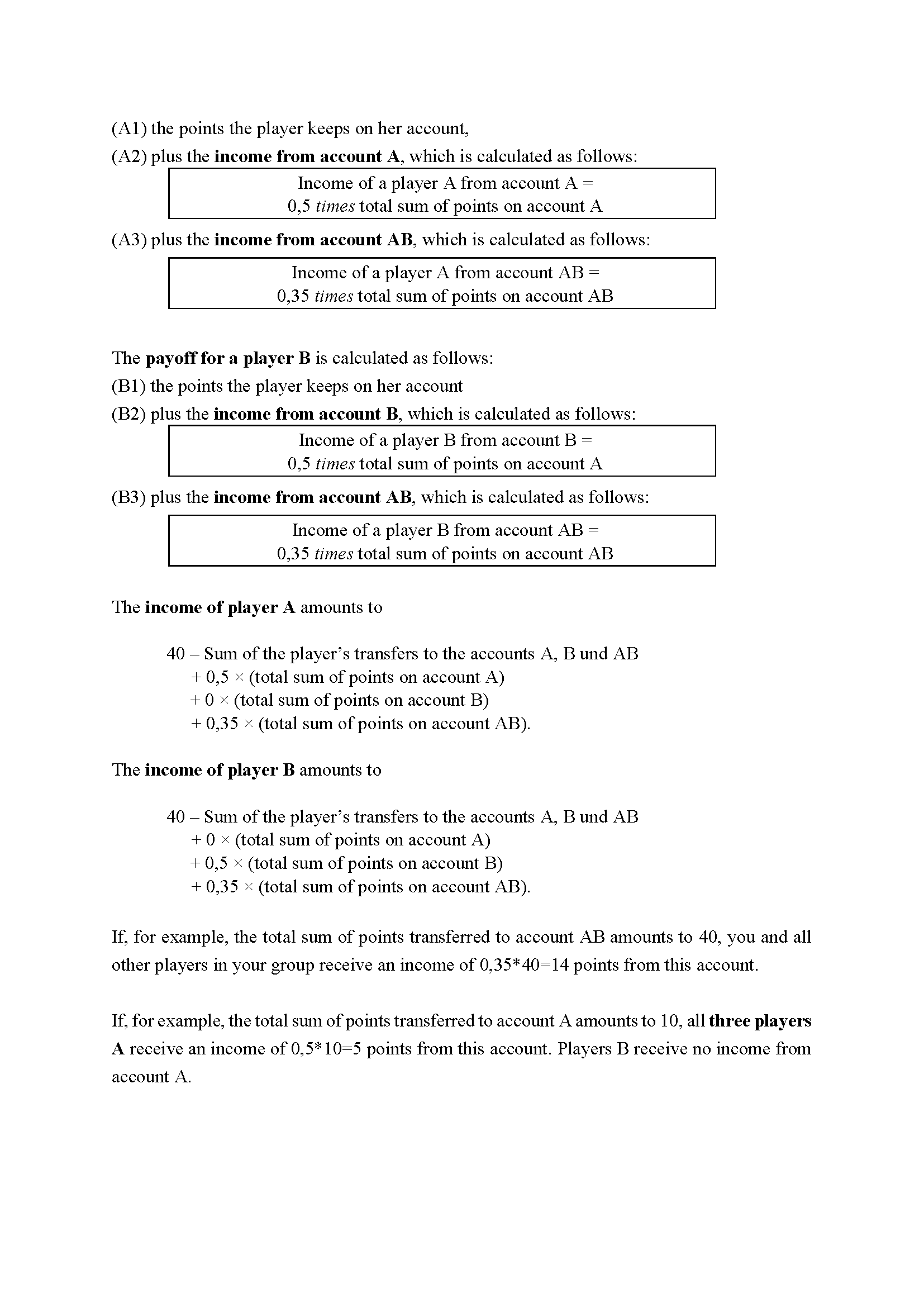

Supplement: Supplementary file 1 — Electronic supplementary material 1 (ZIP 3076 kb) [file 10683_2021_9705_MOESM1_ESM.zip › appendix section 5/Instructions for online appendix/Instructions_Part1_T1-2-4_Page_2.png]

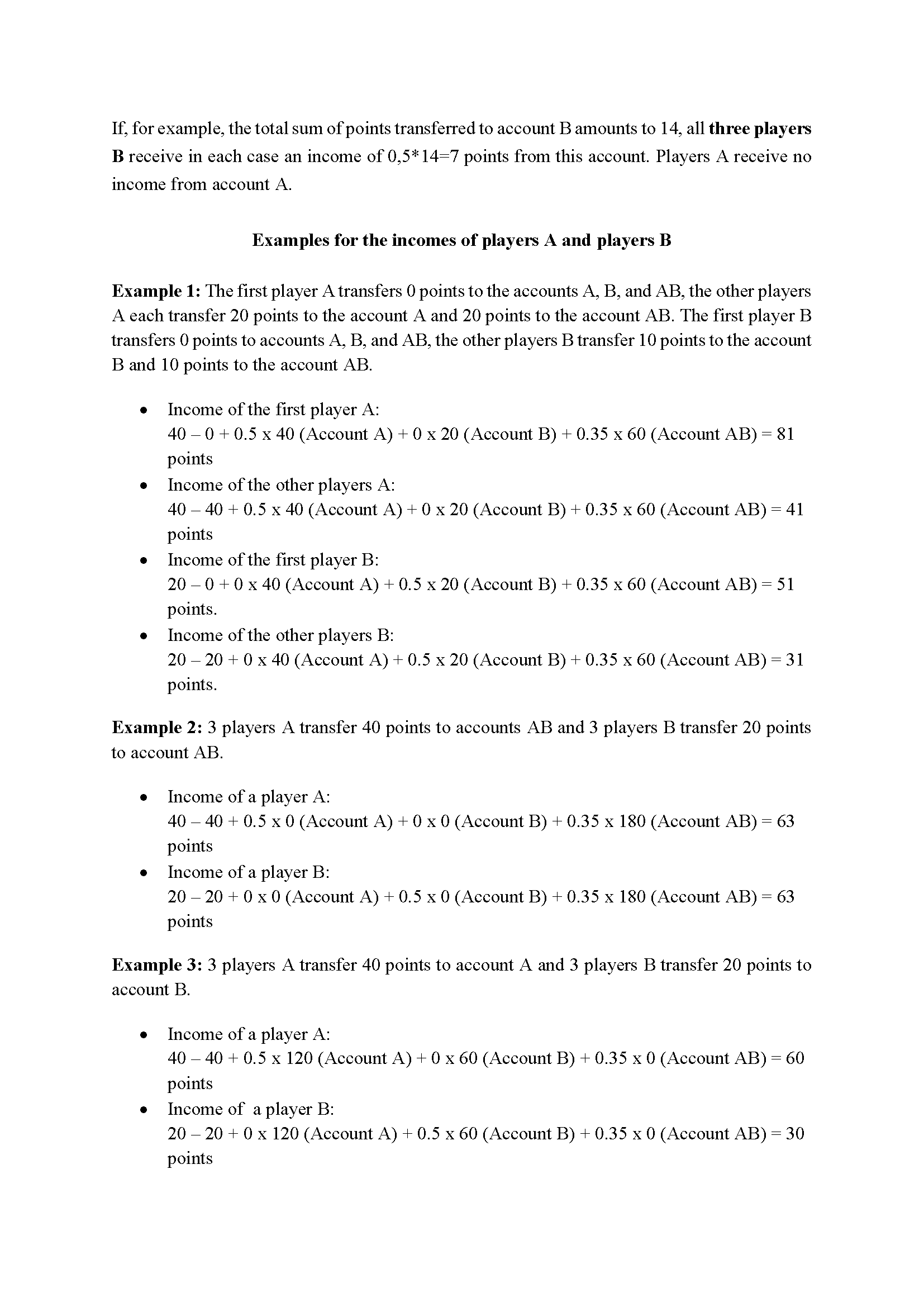

Supplement: Supplementary file 1 — Electronic supplementary material 1 (ZIP 3076 kb) [file 10683_2021_9705_MOESM1_ESM.zip › appendix section 5/Instructions for online appendix/Instructions_Part1_T1-2-4_Page_3.png]

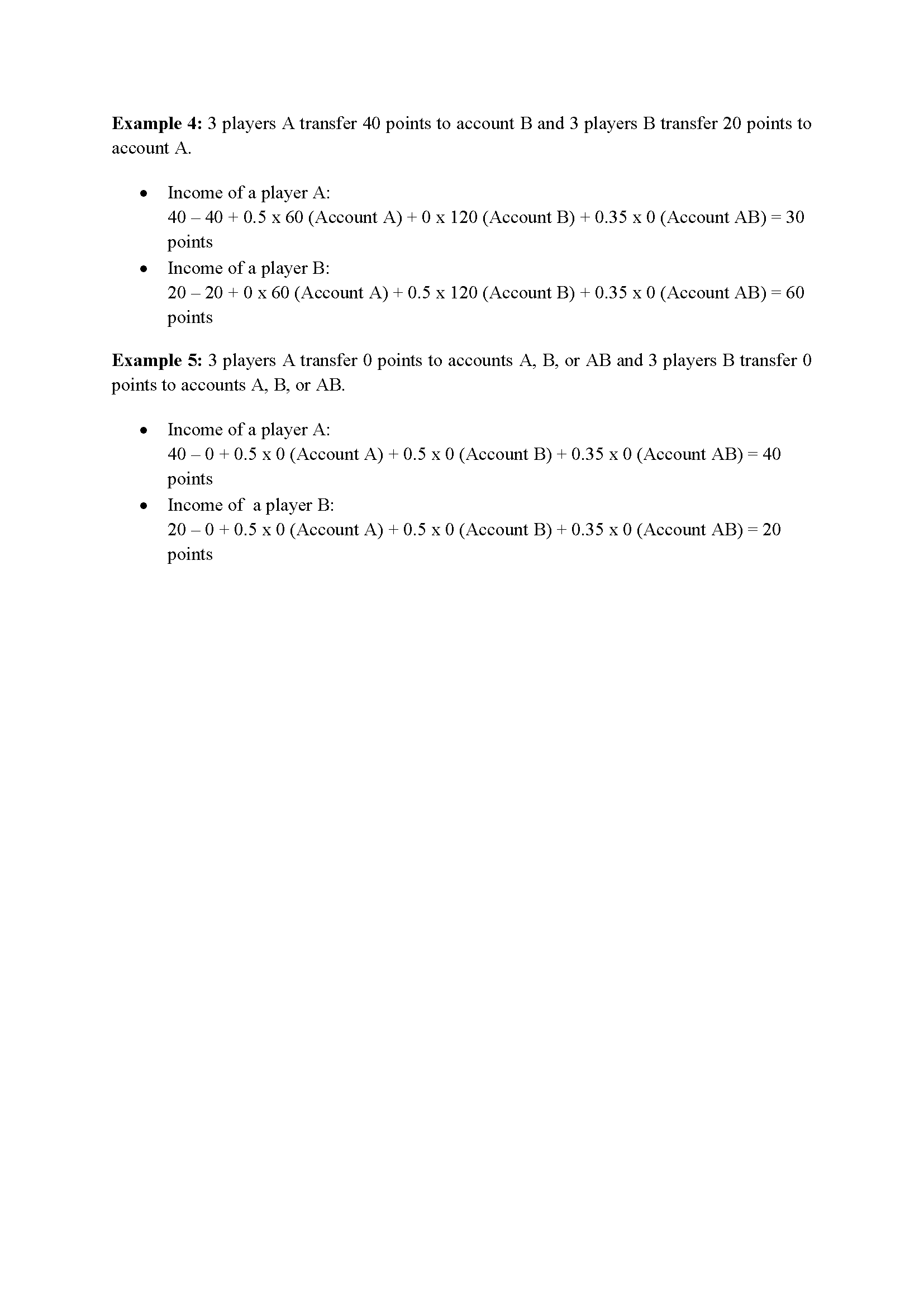

Supplement: Supplementary file 1 — Electronic supplementary material 1 (ZIP 3076 kb) [file 10683_2021_9705_MOESM1_ESM.zip › appendix section 5/Instructions for online appendix/Instructions_Part1_T1-2-4_Page_4.png]

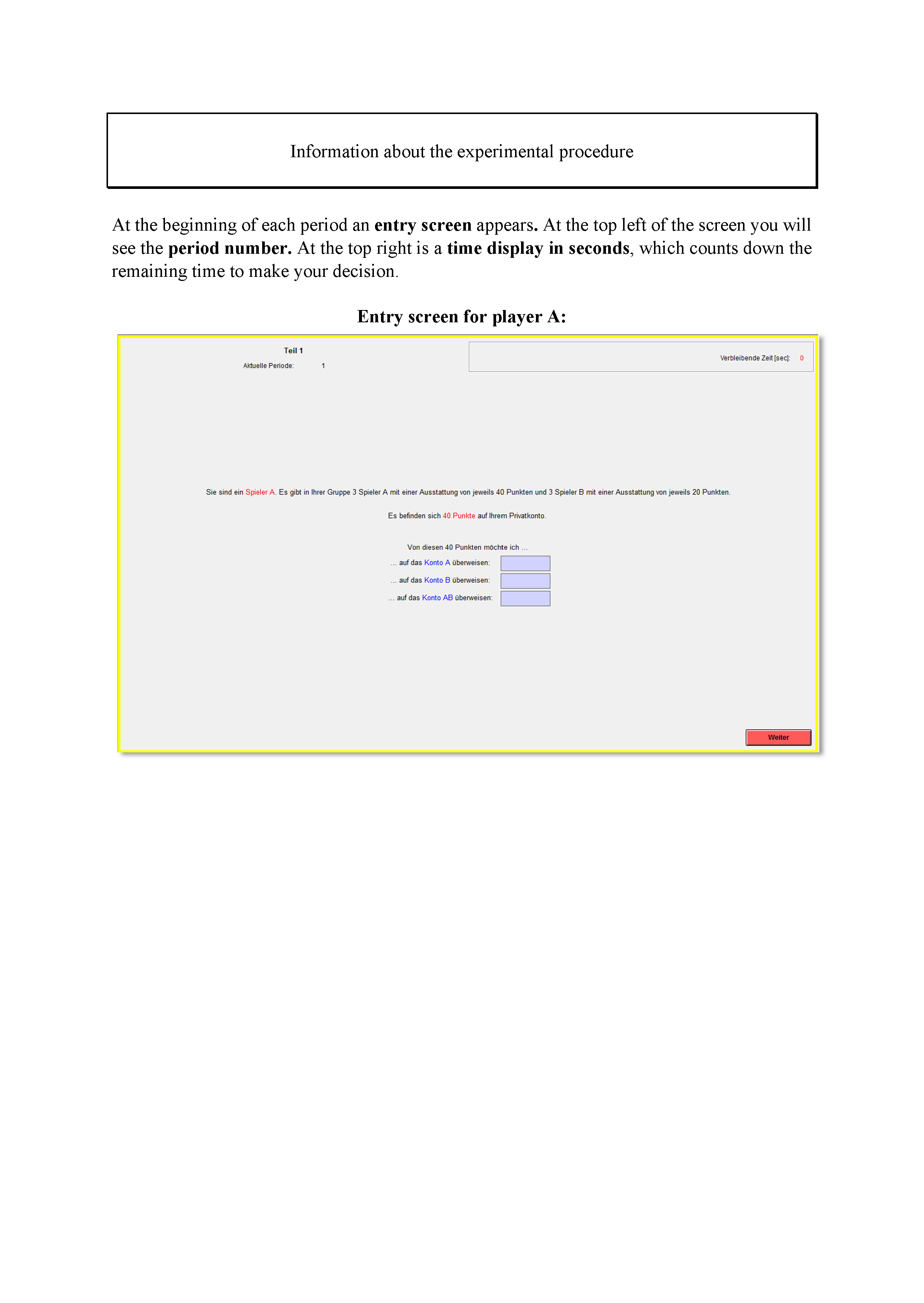

Supplement: Supplementary file 1 — Electronic supplementary material 1 (ZIP 3076 kb) [file 10683_2021_9705_MOESM1_ESM.zip › appendix section 5/Instructions for online appendix/Instructions_Part1_T1-2-4_Page_5.png]

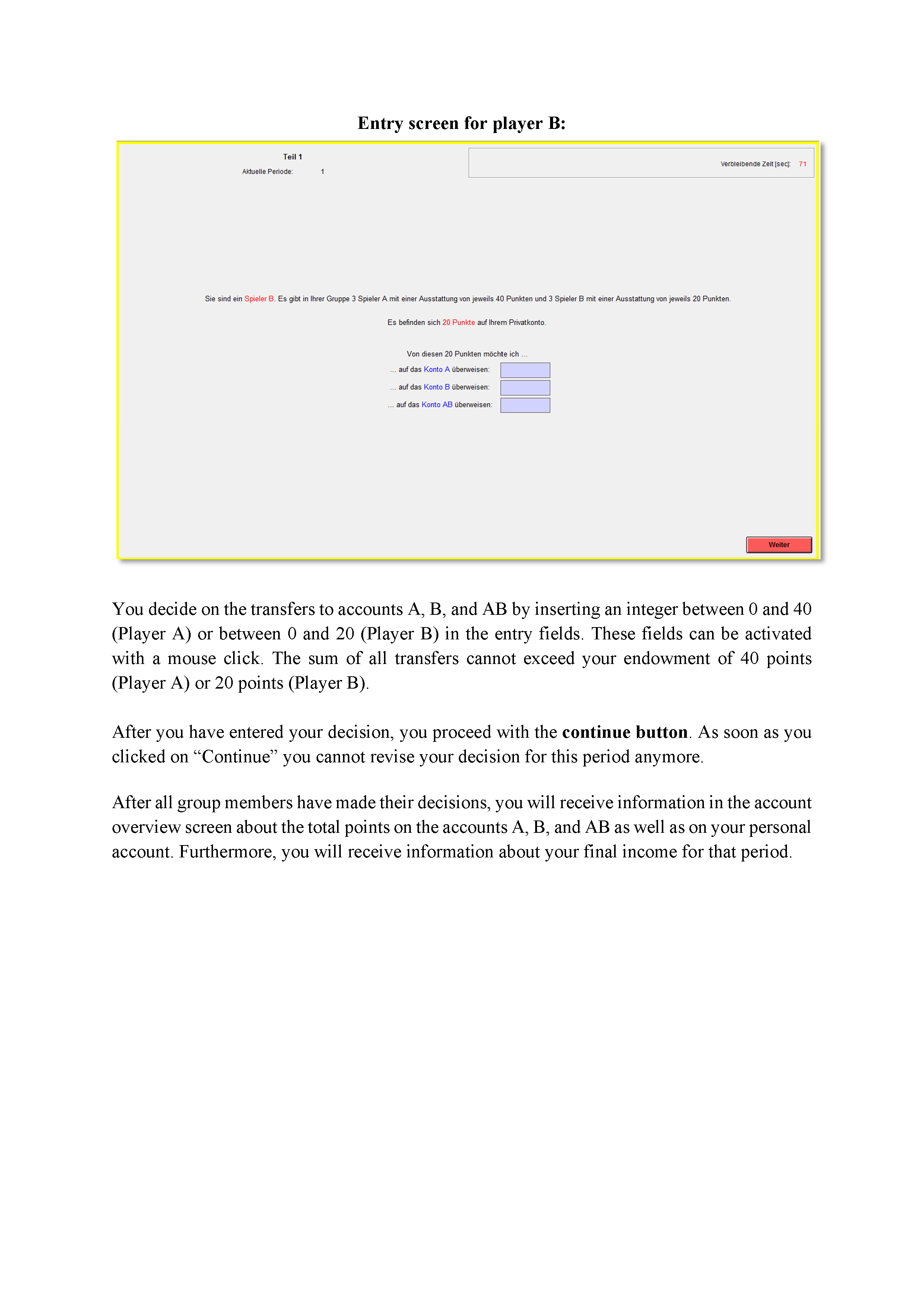

Supplement: Supplementary file 1 — Electronic supplementary material 1 (ZIP 3076 kb) [file 10683_2021_9705_MOESM1_ESM.zip › appendix section 5/Instructions for online appendix/Instructions_Part1_T1-2-4_Page_6.png]

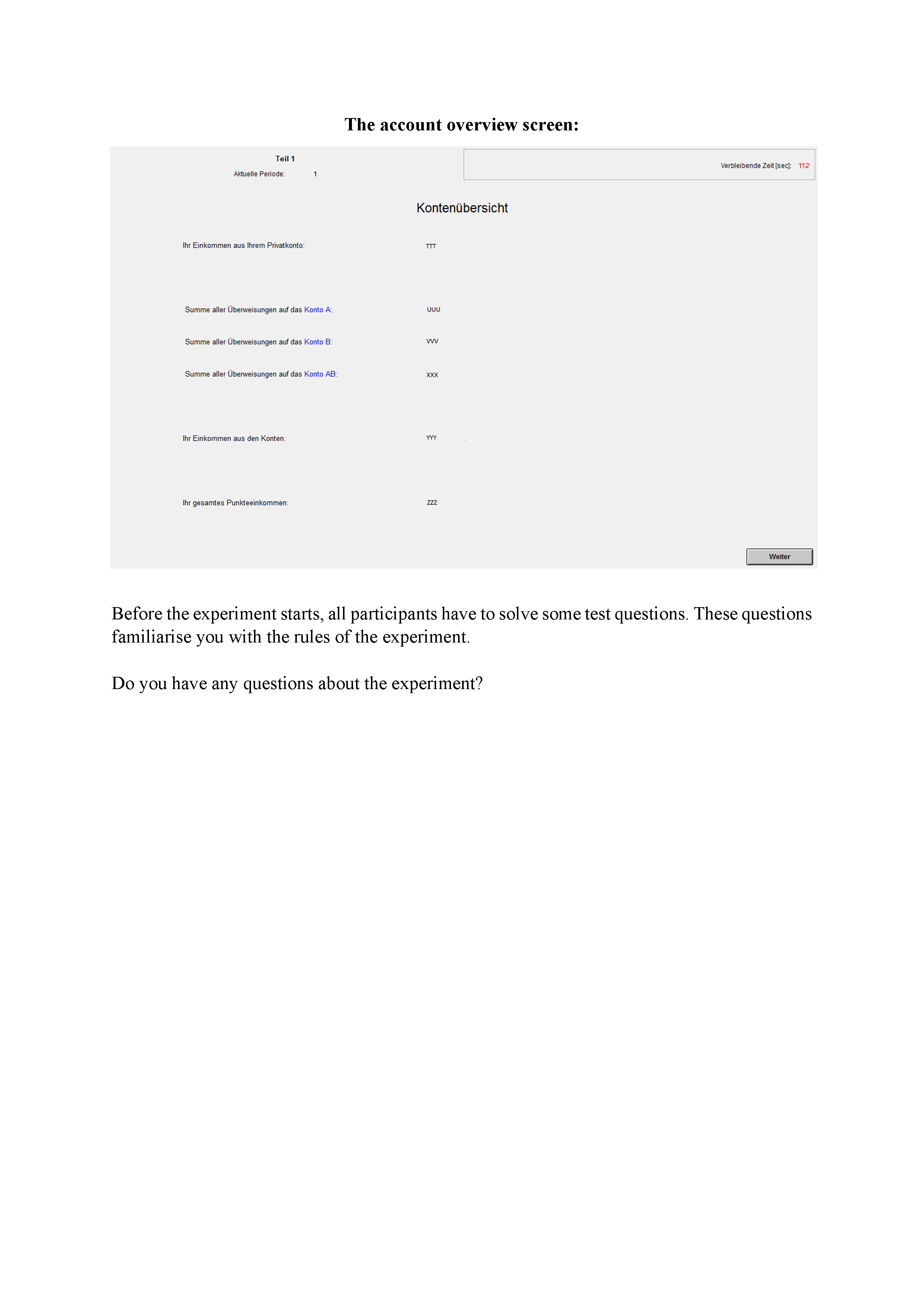

Supplement: Supplementary file 1 — Electronic supplementary material 1 (ZIP 3076 kb) [file 10683_2021_9705_MOESM1_ESM.zip › appendix section 5/Instructions for online appendix/Instructions_Part1_T1-2-4_Page_7.png]

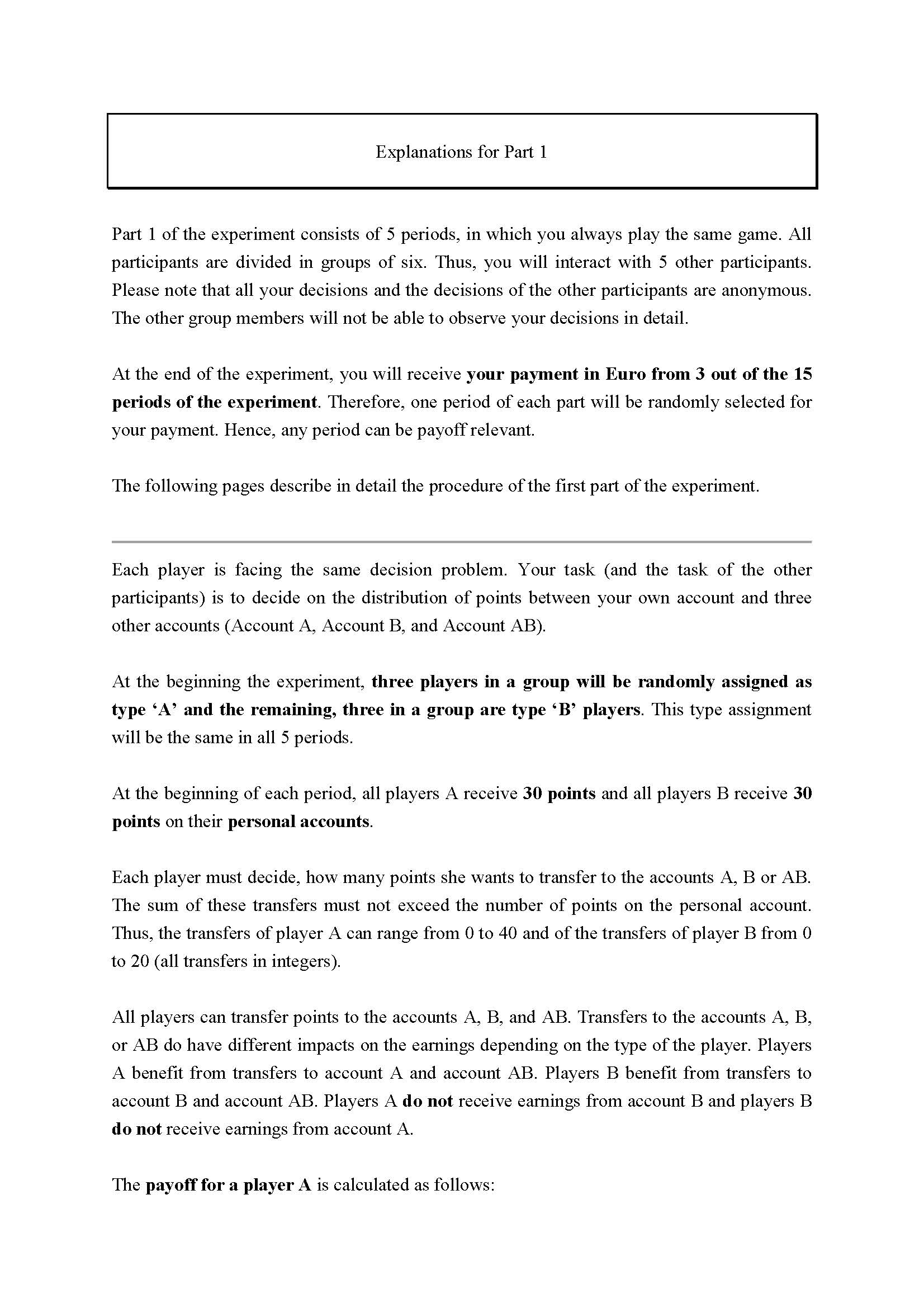

Supplement: Supplementary file 1 — Electronic supplementary material 1 (ZIP 3076 kb) [file 10683_2021_9705_MOESM1_ESM.zip › appendix section 5/Instructions for online appendix/Instructions_Part1_T3_Page_1.jpg]

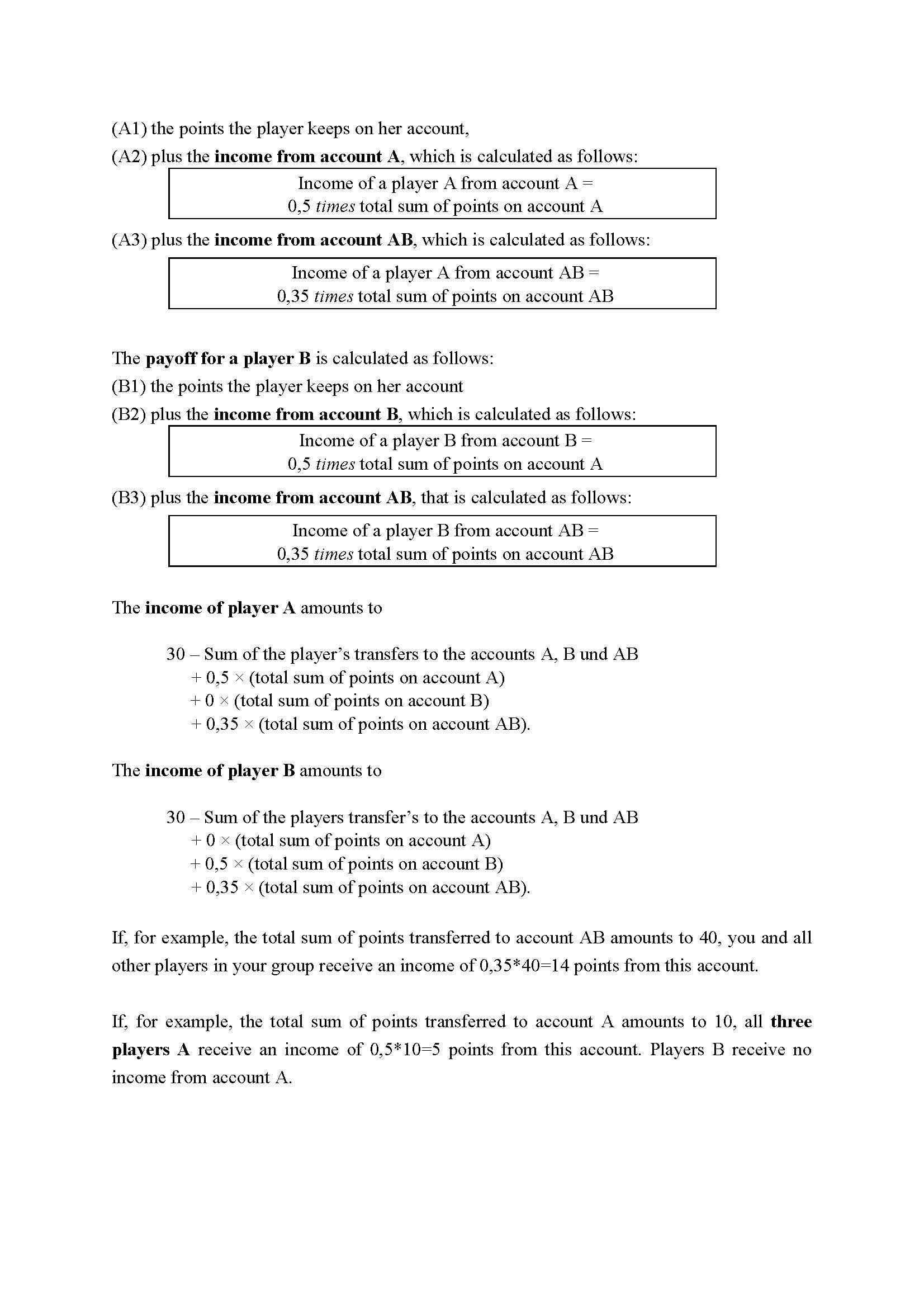

Supplement: Supplementary file 1 — Electronic supplementary material 1 (ZIP 3076 kb) [file 10683_2021_9705_MOESM1_ESM.zip › appendix section 5/Instructions for online appendix/Instructions_Part1_T3_Page_2.jpg]

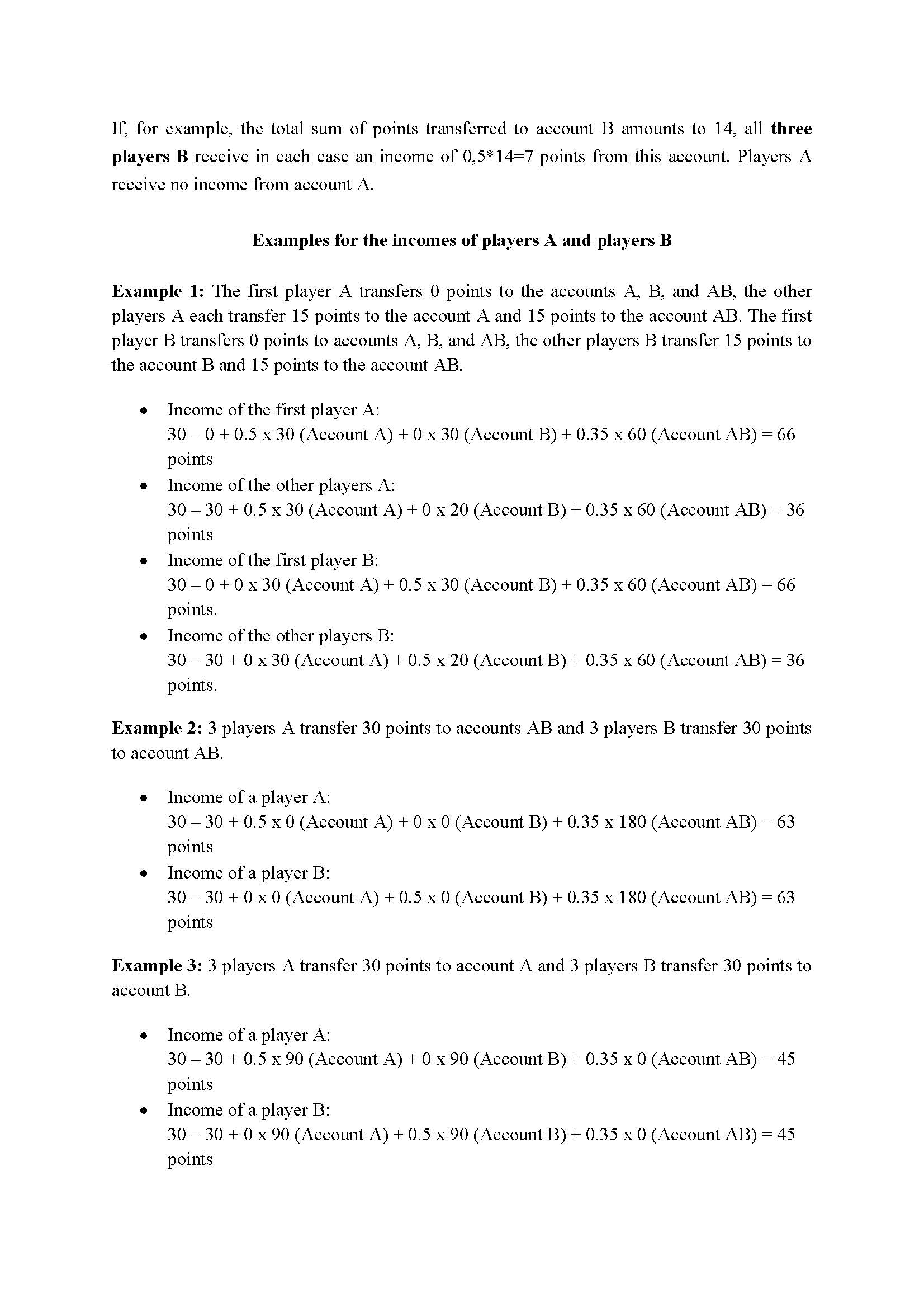

Supplement: Supplementary file 1 — Electronic supplementary material 1 (ZIP 3076 kb) [file 10683_2021_9705_MOESM1_ESM.zip › appendix section 5/Instructions for online appendix/Instructions_Part1_T3_Page_3.jpg]

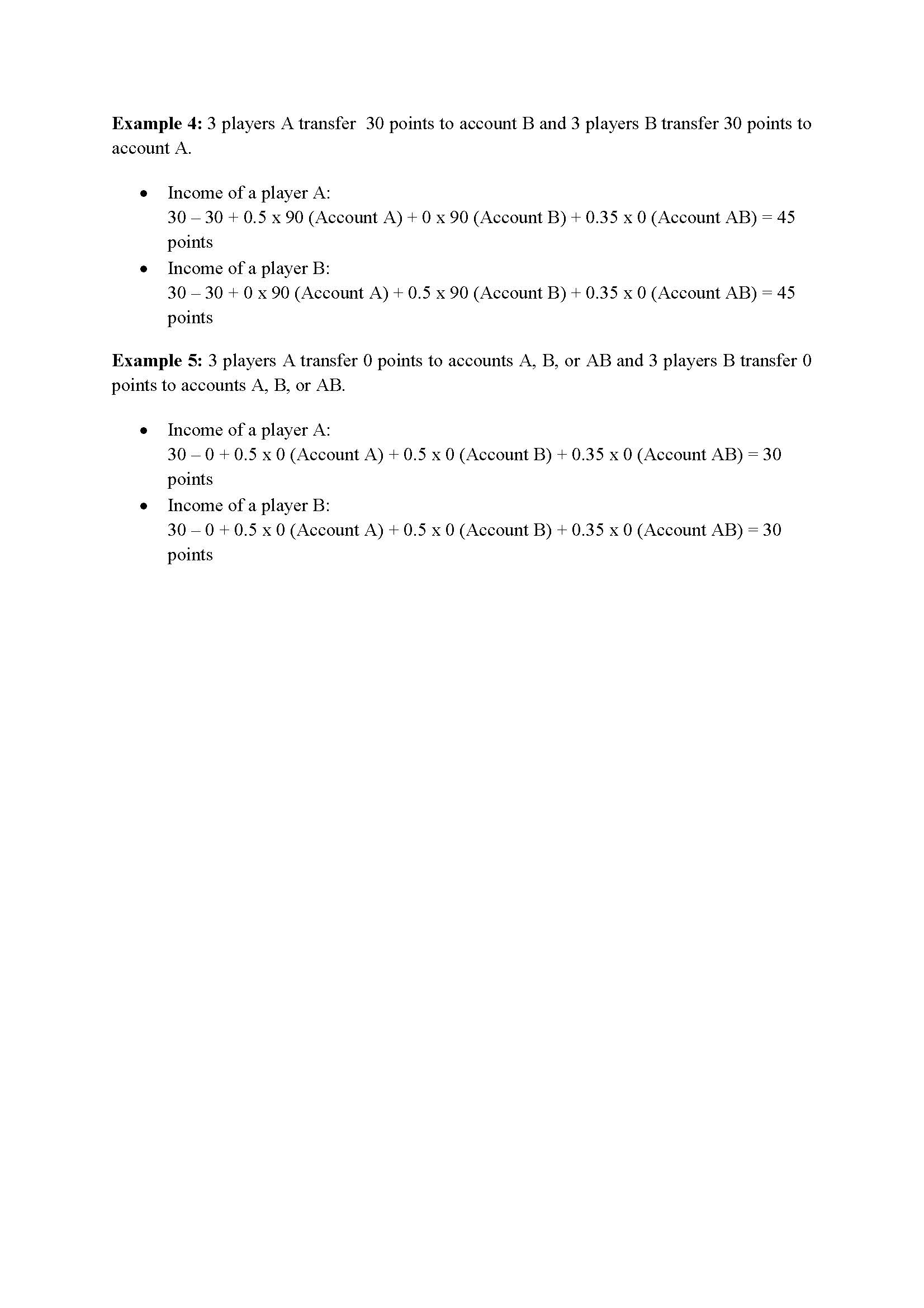

Supplement: Supplementary file 1 — Electronic supplementary material 1 (ZIP 3076 kb) [file 10683_2021_9705_MOESM1_ESM.zip › appendix section 5/Instructions for online appendix/Instructions_Part1_T3_Page_4.jpg]

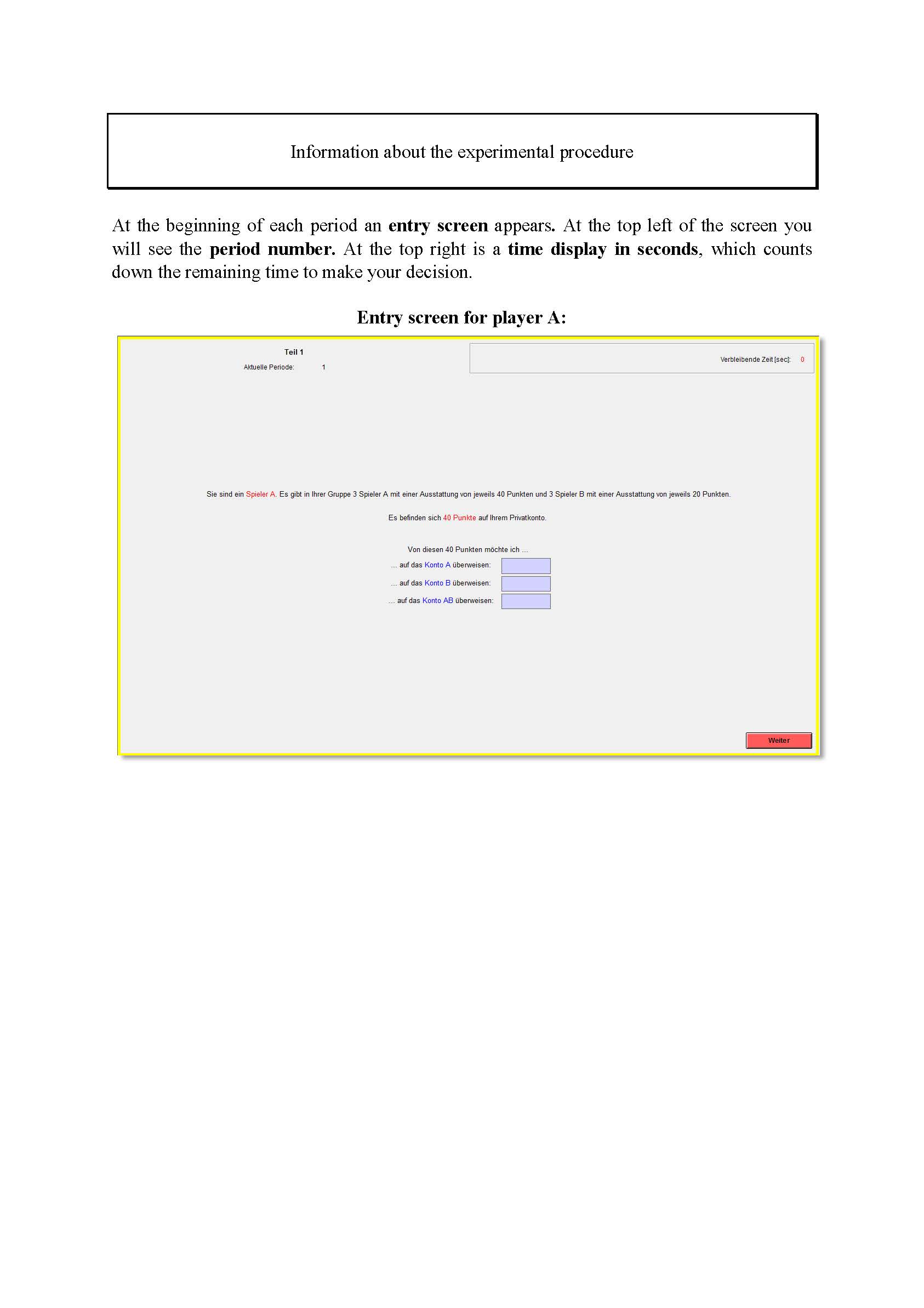

Supplement: Supplementary file 1 — Electronic supplementary material 1 (ZIP 3076 kb) [file 10683_2021_9705_MOESM1_ESM.zip › appendix section 5/Instructions for online appendix/Instructions_Part1_T3_Page_5.jpg]

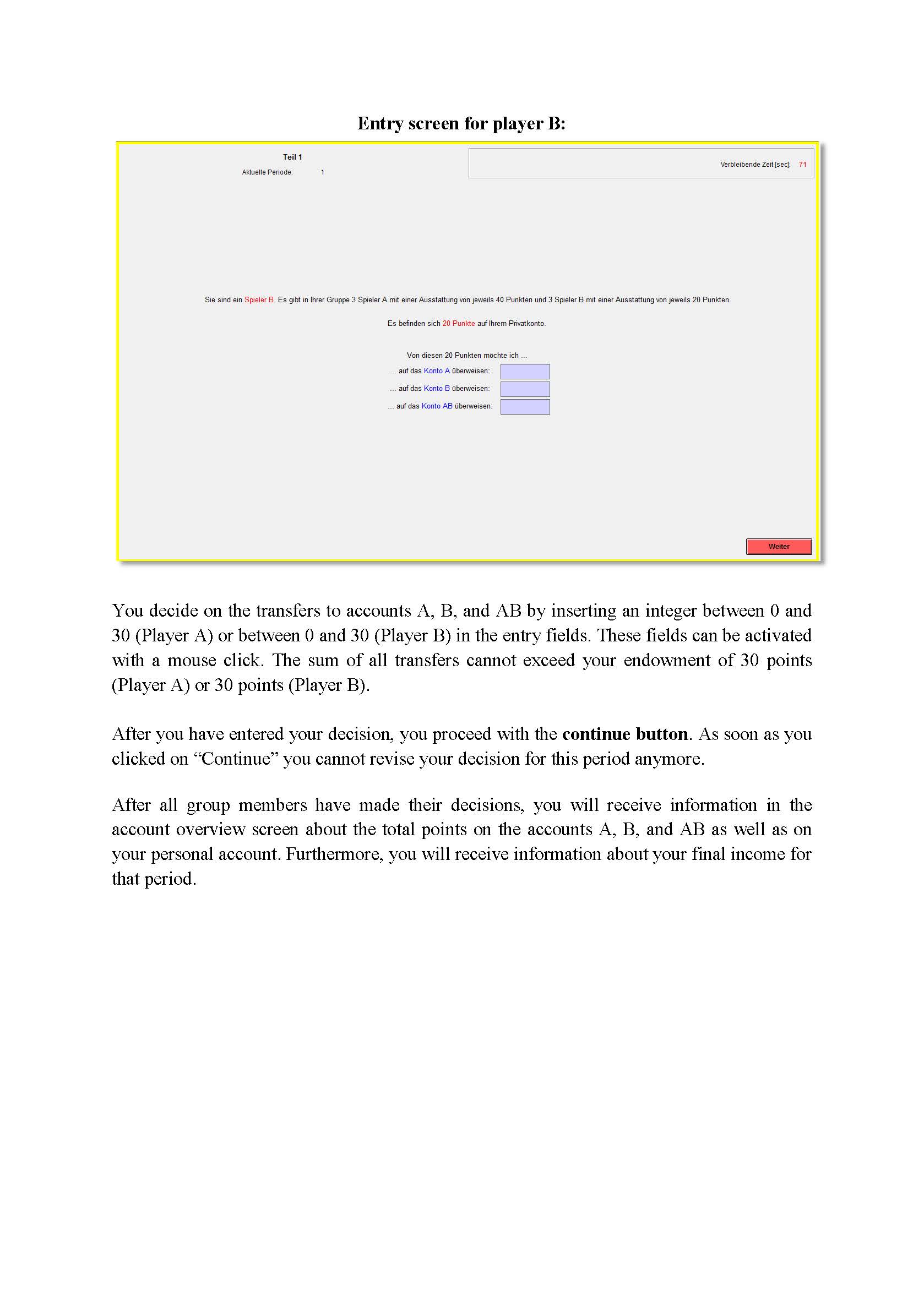

Supplement: Supplementary file 1 — Electronic supplementary material 1 (ZIP 3076 kb) [file 10683_2021_9705_MOESM1_ESM.zip › appendix section 5/Instructions for online appendix/Instructions_Part1_T3_Page_6.jpg]

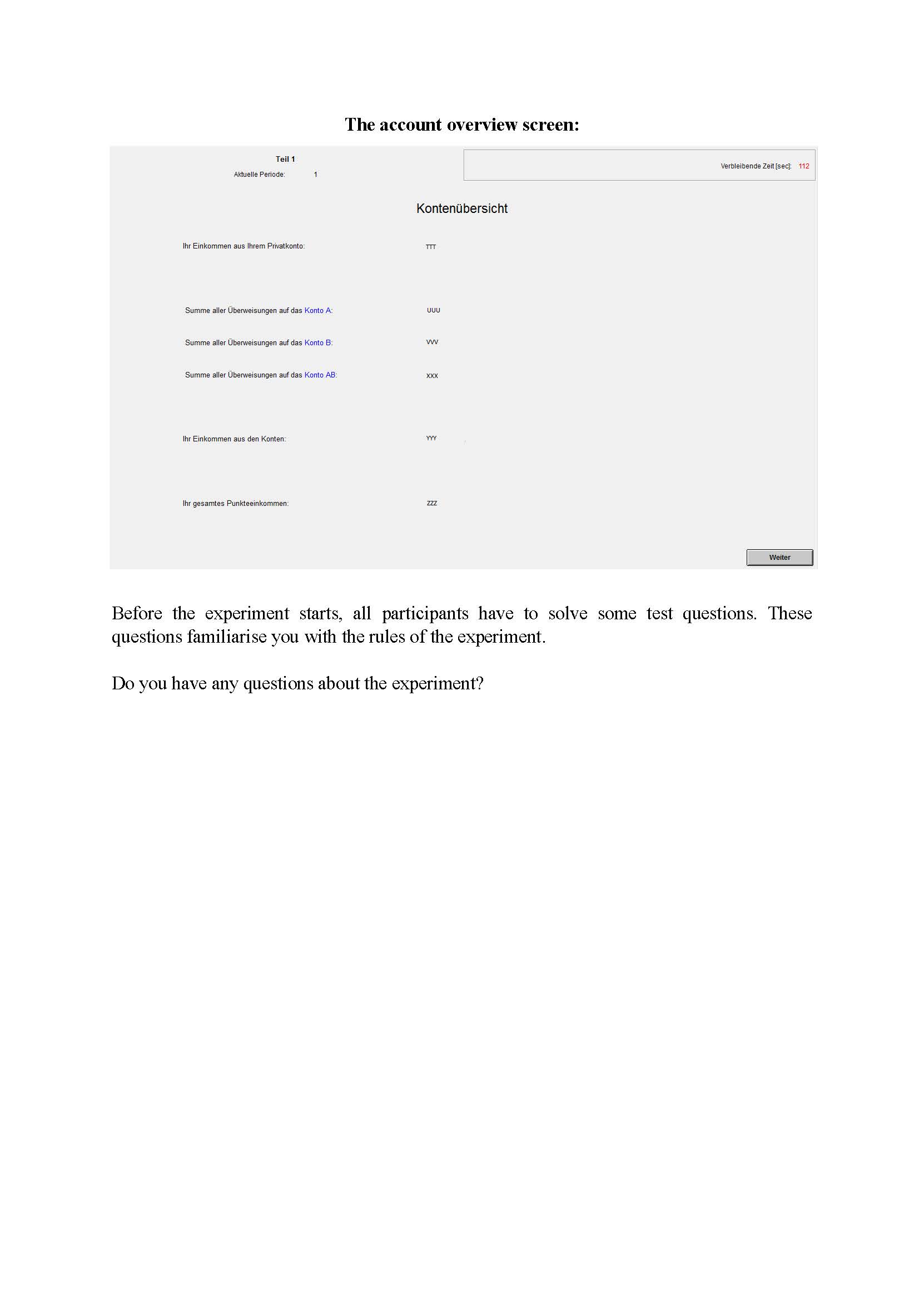

Supplement: Supplementary file 1 — Electronic supplementary material 1 (ZIP 3076 kb) [file 10683_2021_9705_MOESM1_ESM.zip › appendix section 5/Instructions for online appendix/Instructions_Part1_T3_Page_7.jpg]

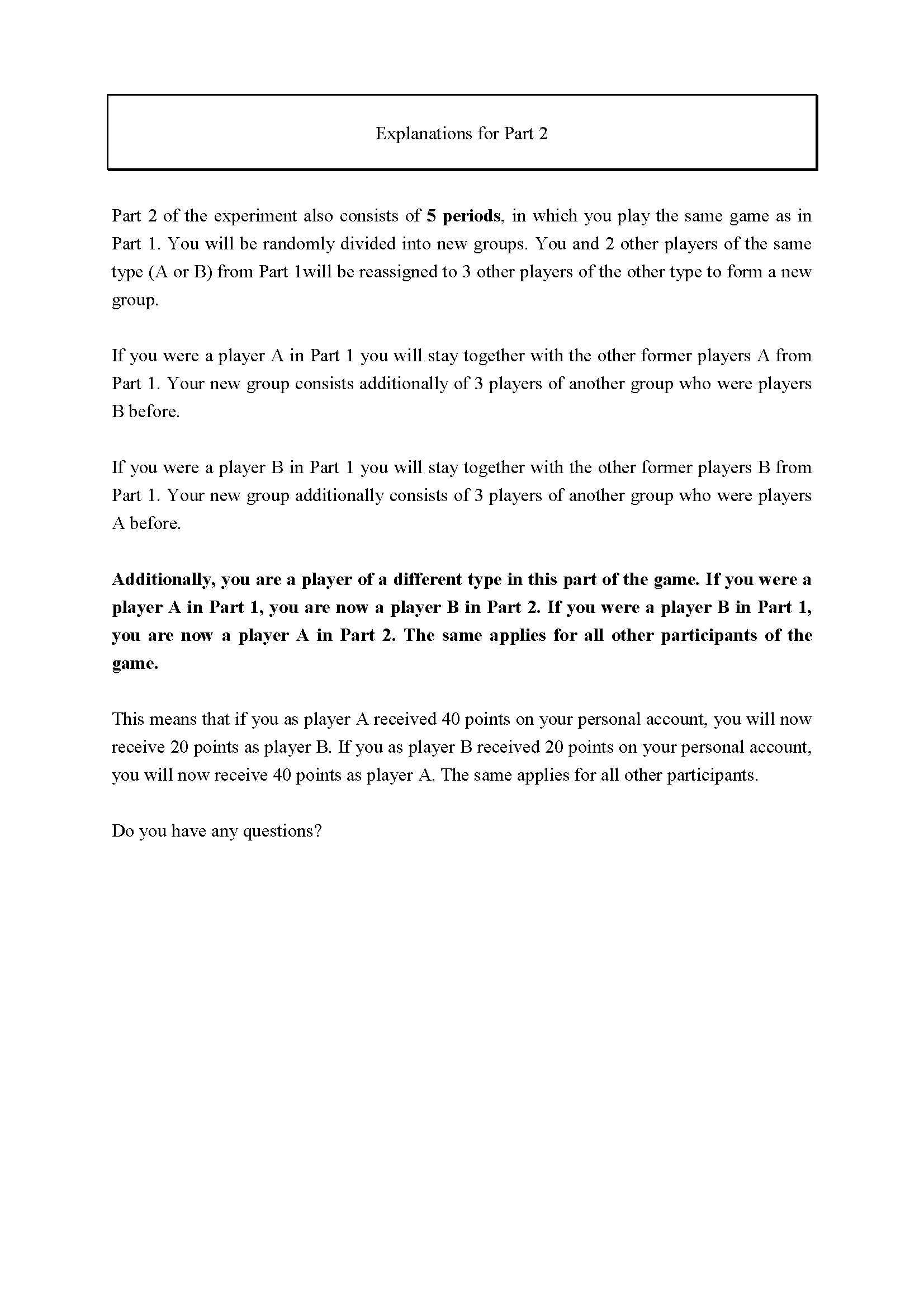

Supplement: Supplementary file 1 — Electronic supplementary material 1 (ZIP 3076 kb) [file 10683_2021_9705_MOESM1_ESM.zip › appendix section 5/Instructions for online appendix/Instructions_Part2_T1.png]

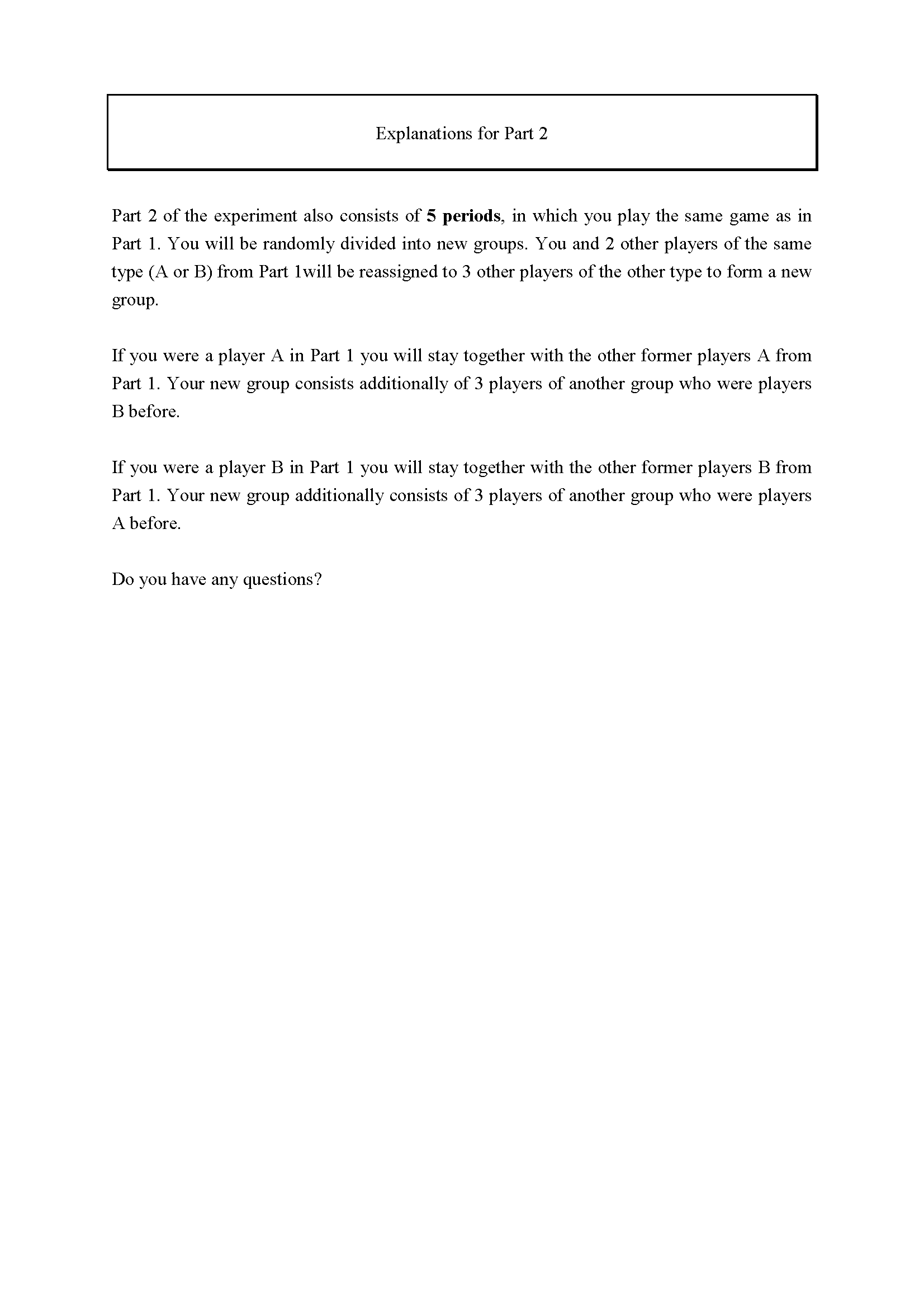

Supplement: Supplementary file 1 — Electronic supplementary material 1 (ZIP 3076 kb) [file 10683_2021_9705_MOESM1_ESM.zip › appendix section 5/Instructions for online appendix/Instructions_Part2_T2-3.png]

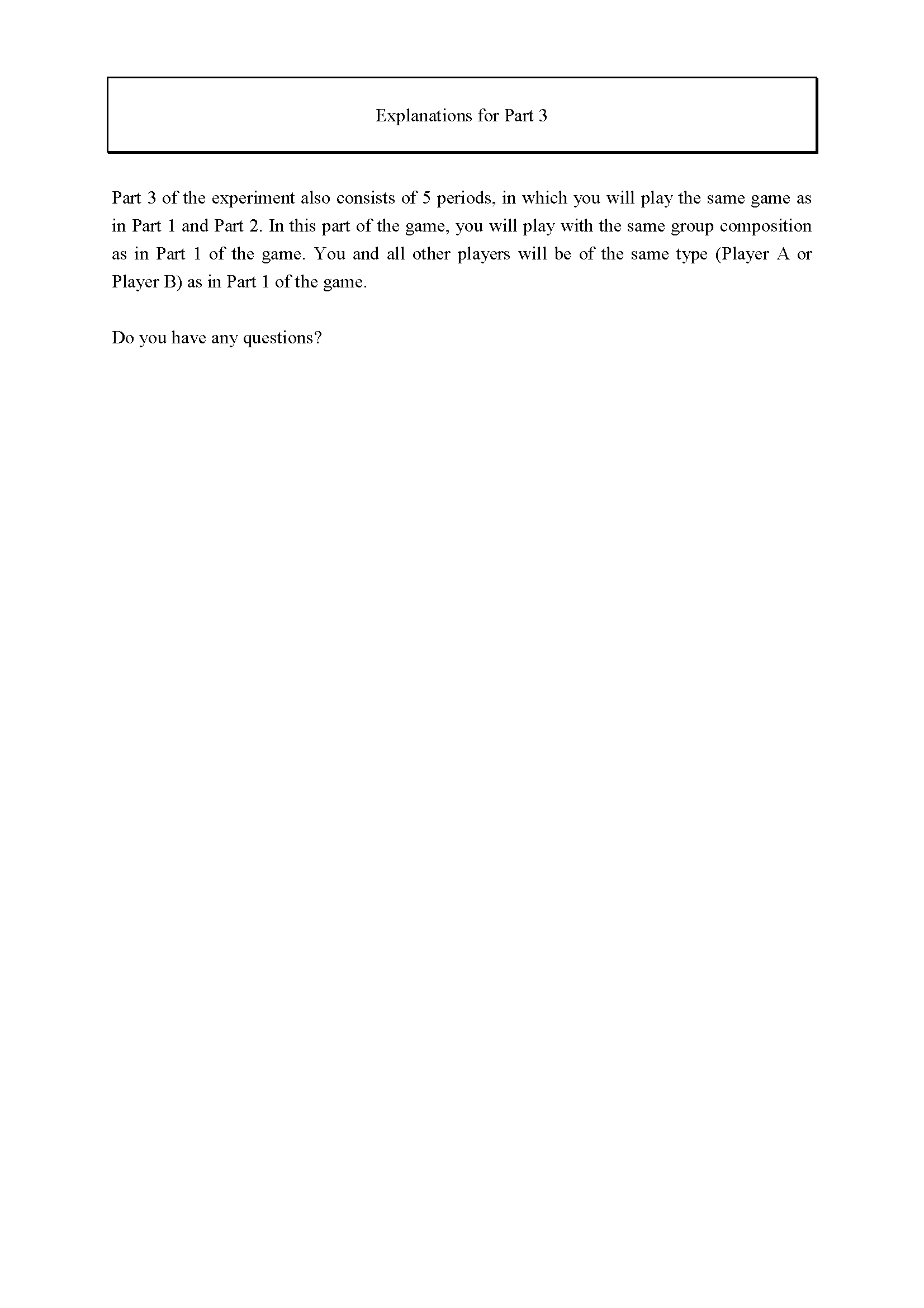

Supplement: Supplementary file 1 — Electronic supplementary material 1 (ZIP 3076 kb) [file 10683_2021_9705_MOESM1_ESM.zip › appendix section 5/Instructions for online appendix/Instructions_Part3_T1.png]
